# Phylogeographic epidemiology of *Dabie bandavirus* in East Asia: divergent transmission networks and genotype‑linked clinical severity

**DOI:** 10.1186/s40249-026-01500-2

**Published:** 2026-09-11

**Authors:** Letian Fang, Jiluo Liu, Jimin Sun, Ping Li, Zhongfa Wang, Hongxia Ni, Ling Ye, Yiwei Shi, Xin Yang, Hanzheng Mu, Peng Li, Yibo Ding, Xiaojie Tan, Jianhua Yin, Wei Liu, Guangwen Cao

**Affiliations:** 1 Department of Epidemiology, Second Military Medical University, Shanghai, 200433 China; 2 Key Laboratory of Biological Defense, Ministry of Education, Shanghai, 200433 China; 3 Shanghai Key Laboratory of Medical Bioprotection, Shanghai, 200433 China; 4 Zhejiang Provincial Center for Disease Control and Prevention, Hangzhou, 310051 Zhejiang China; 5 Health Monitoring Department, Dinghai Center for Disease Control and Prevention of Zhoushan City, Zhoushan, 316000 Zhejiang China; 6 Ningbo Municipal Center for Disease Control and Prevention, Ningbo, 315010 Zhejiang China; 7 Department of Infectious Disease Control and Prevention, Daishan Center for Disease Control and Prevention, Zhoushan, 316200 Zhejiang China; 8 State Key Laboratory of Pathogen and Biosecurity, Academy of Military Medical Sciences, Beijing, 100071 China

**Keywords:** *Dabie bandavirus*, Severe fever with thrombocytopenia syndrome, Phylogeographic analysis, Transmission, Fatality

## Abstract

**Background:**

Severe fever with thrombocytopenia syndrome (SFTS), caused by *Dabie bandavirus* (SFTSV), exhibits geographically decoupled incidence and fatality patterns across East Asia. We aimed to elucidate the distinct ecological drivers and phylogeographic dynamics underlying this inland-coastal epidemiological divergence.

**Methods:**

Integrating 1820 high-quality global genomes of SFTSV with well-characterized clinical cohorts (936 patients) and nationwide surveillance data (27,457 cases) from China, we constructed a comprehensive analytical framework. Ecological modeling, Bayesian phylogeography, and genotype–phenotype association analyses were employed to trace the evolutionary trajectories and clinical implications of the virus.

**Results:**

A pronounced “inland-high-incidence vs. coastal-high-fatality” pattern of SFTS was identified. The incidence of SFTS exhibited divergent sensitivities to meteorological factors; inland transmission was sensitive to thermal fluctuations, whereas coastal dynamics were constrained by a sunshine threshold (> 200 h/month). In contrast, spatial divergence in clinical severity correlated with the distribution of regional viral genetic structures. Inland regions mainly co-circulated genotypes A, C, and D, while coastal regions were dominated by genotype B. Zhejiang province was identified as a genetic hub with significantly higher recombination frequencies than inland regions (11.0% vs. 3.5%, *P* < 0.001). Bayesian phylogeographic inference indicated frequent lineage exchange of Zhejiang province in China with the Republic of Korea and Japan. Clinically, genotypes B and D were associated with elevated mortality in coastal and inland regions, respectively, suggesting that the severe coastal phenotype is shaped by its genotype B-dominated structure. Additionally, the RdRp-N828S mutation emerged as a robust molecular correlate of fatal outcomes, warranting further functional validation.

**Conclusions:**

Divergent meteorological factors and plausible maritime transmission networks may underlie the geographically decoupled epidemiology of SFTS. These findings highlight that risk assessment must extend beyond incidence alone and provide a phylogeographically informed framework for targeted surveillance and genotype-specific interventions in high-risk hotspots.

**Graphical Abstract:**

**Figure:**
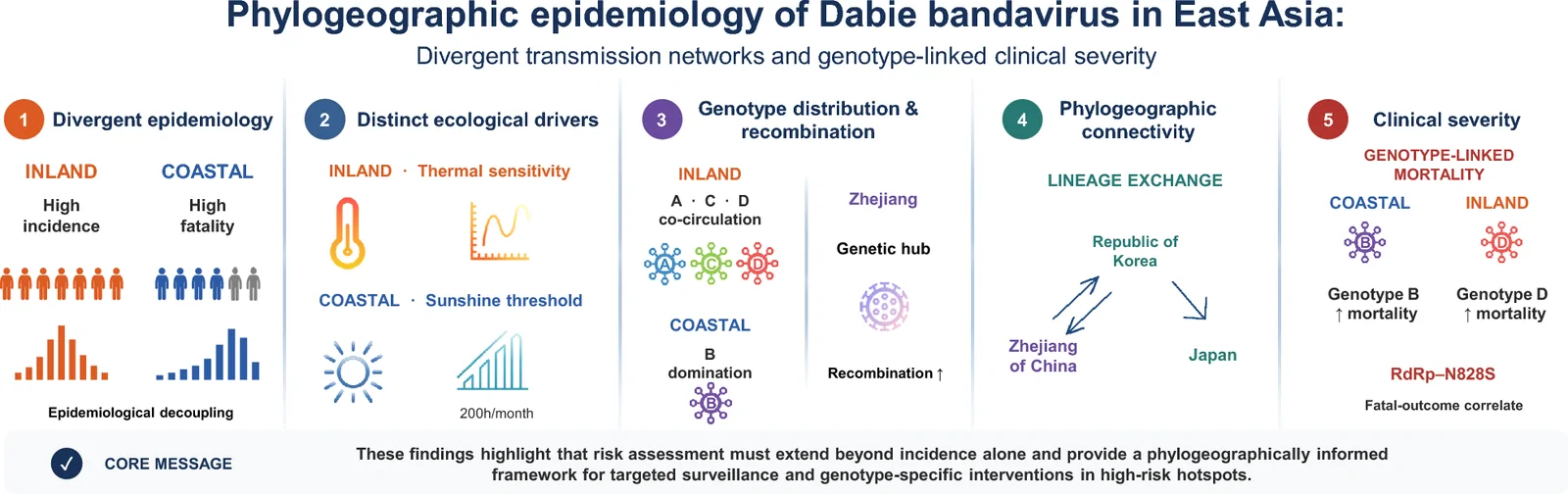

**Supplementary Information:**

The online version contains supplementary material available at https://doi.org/10.1186/s40249-026-01500-2.

## Background

Severe fever with thrombocytopenia syndrome (SFTS) is an emerging tick-borne zoonosis caused by *Dabie bandavirus*, commonly referred to as severe fever with thrombocytopenia syndrome virus (SFTSV). Since its initial identification in central China in 2009 [1], SFTSV has been increasingly reported across multiple provinces in China and neighboring countries, including Japan, the Republic of Korea, and Thailand, posing a significant and expanding threat to regional public health [2–5]. The virus is maintained in an enzootic cycle primarily involving a range of wild and domestic animal reservoirs, as well as hard ticks, most notably *Haemaphysalis longicornis*, alongside other species such as *Amblyomma testudinarium* and *H. hystricis* [6–8]. Human SFTS occurs primarily through tick bites [9], with documented cases of nosocomial and human-to-human transmission [10, 11], particularly through contact with blood or bloody fluids in cluster outbreaks [12], complicating the epidemiology and elevating the mortality risk of SFTS. Furthermore, potential SFTSV re-infections suggest waning immunity, which further challenges disease management [13]. The SFTSV genome comprises three single-stranded, negative-sense RNA segments: the L segment, which encodes the RNA-dependent RNA polymerase (RdRp); the M segment, which encodes the envelope glycoproteins N (Gn) and C (Gc); and the S segment, which encodes the nucleoprotein (NP) and the non-structural proteins (NSs). Genetic variations across these segments can influence viral replication efficiency, host cell entry, and immune evasion, thereby contributing to differences in disease severity and clinical outcomes.

Despite accumulating evidence of substantial genetic diversity, a consensus on the SFTSV genotype classification remains lacking. The proposed frameworks range from a two-lineage model (Chinese vs. Japanese–Korean) to more complex systems involving up to fourteen lineages [14–19]. Recent high-resolution analyses suggest that SFTSV diversity may be more accurately captured by geographically structured clades rather than numerous genotypes [19]. This genomic diversification is actively driven by distinct evolutionary pressures, such as strong purifying selection in the L segment and episodic positive selection in the M and S segments, coupled with frequent segment-specific recombination and reassortment [16, 19]. The origin and dispersal of these lineages across East Asia remain heavily debated. Phylogeographic studies have proposed multiple possible origins, including Dabie Mountain area, Zhejiang province of China, the Republic of Korea, and Japan [15, 16, 20–22]. Recent studies have highlighted the accelerated cross-border dispersal of specific lineages, particularly the L2 lineage, which appears to be driven by its superior adaptation to the invasive *H. longicornis* tick [14]. Notably, spatial reconstruction of viral migration has shown considerable overlap between SFTSV dissemination routes and flyways of migratory birds [16, 22]. This spatial congruence suggests that migratory birds might act as vehicles for trans-oceanic viral dissemination, although the mechanisms warrant further ecological validation.

A critical and unresolved aspect of this complex evolutionary landscape is the pronounced geographic heterogeneity in clinical severity. The overall case fatality rate (CFR) in China is approximately 7%, whereas rates in the Republic of Korea and Japan reach 18%–40% [6, 23]*.* Accumulating evidence suggests that specific viral genotypes are associated with disease severity. For instance, genotype B in the Republic of Korea and Clade IV in China were independently associated with high fatality rates [17, 18]. These observations imply that genotype- or clade-specific virulence may drive the regional differences in clinical outcomes [20, 24]. However, broad geographic genotype distributions alone cannot fully explain the epidemiological contrasts observed across East Asia. A notable example is the geographic decoupling of SFTS epidemiology. Inland hyperendemic regions in central China (e.g., the Dabie Mountain area) exhibit high incidence but relatively lower fatality, whereas coastal regions (e.g., Zhejiang province) report lower incidence yet disproportionately higher fatality [25]. This distinct inland-coastal dichotomy suggests that SFTSV transmission and pathogenesis might be shaped by a confluence of region-specific ecological factors and divergent phylogeographic trajectories, which have rarely been examined using integrated analytical frameworks.

Moving beyond descriptive accounts of geographic variation toward a mechanistic understanding of these regional disparities requires an approach that simultaneously incorporates the clinical, genomic, and ecological dimensions. We previously identified Zhejiang province of China within a dual terrestrial–maritime transmission network connecting China, Japan, and the Republic of Korea, where distinct viral populations circulate in the inland and coastal regions [19]. Building on this foundation, the present study integrates clinical data from laboratory-confirmed SFTS patients in Henan (representing an inland region) and Zhejiang (an eastern coastal hub) provinces, coupled with an expanded spatiotemporal phylogenetic framework comprising 1820 SFTSV genomes. Specifically, our objectives were to: (i) delineate the divergent meteorological drivers of regional incidence and compare the clinical profiles for fatal SFTS outcomes in distinct ecological settings; (ii) characterize the genetic diversity, evolutionary dynamics, and plausible cross-sea lineage exchange within and beyond the coastal Zhejiang province of China; and (iii) determine the observational association between specific viral genetic clusters/lineages and clinical severity. This study aims to provide a phylogeographically informed framework for genotype-oriented precision surveillance and guide targeted intervention strategies for SFTS in high-risk hotspots.

## Methods

### Human SFTS case reports

SFTS is classified as a Category B notifiable infectious disease in Chinese mainland. All healthcare facilities are required to report suspected and confirmed cases of SFTS within 24 h of detection to the National Notifiable Infectious Diseases Surveillance System. For this study, we obtained data on SFTS cases reported between 2010 and 2023 from the China Information System for Disease Control and Prevention. The regional and provincial population sizes used as the denominators for incidence calculations were derived from the Seventh National Population Census of China conducted in 2020. The cumulative incidence of SFTS was calculated by dividing the total number of incident cases reported from 2010 to 2023 by the corresponding population size. The CFR was determined by dividing the number of fatal cases by the total number of reported cases.

### SFTS clinical cohorts and SFTSV detection

Two clinical cohorts were established for this study, comprising a total of 936 laboratory-confirmed SFTS cases from Xinyang city (Henan province) and the coastal regions of Zhoushan city and Ningbo city (Zhejiang province). Between 2011 and 2021, patients were enrolled from 154 hospitals in Xinyang city (*n* = 805) [18] and multiple facilities in Zhejiang province (*n* = 131), including Daishan County People’s Hospital and the Centers for Disease Control and Prevention in both Daishan county and Ningbo city. Based on the National Guideline for SFTS Prevention and Control [26], laboratory-confirmed cases were defined as patients who presented with acute fever (≥ 38.0 °C), clinical symptoms (e.g., gastrointestinal distress or hemorrhage), and laboratory findings of thrombocytopenia and leukocytopenia combined with positive detection of SFTSV RNA or isolation of the virus in cell culture. Demographic, clinical, and laboratory data were collected at the time of admission and during hospitalization. Patient outcomes were determined via follow-up phone calls or home visits within 14 days of discharge. Whole blood samples were obtained at admission and during hospitalization and serum was isolated and used for sequencing analysis. Briefly, viral RNA was extracted from 200 μl of serum using the QIAsymphony Virus/Bacteria Mini Kit (Qiagen, Hilden, Germany), according to the manufacturer’s instructions. Quality control was performed using a commercial SFTSV reverse transcription-polymerase chain reaction (RT-PCR) kit (ZJ Bio-Tech, Shanghai, China) which included primers, probes, an internal control, and positive and negative standards. Real-time PCR was performed using an ABI 7500 Real-Time PCR System (Applied Biosystems). A cycle threshold value of ≤ 37 was defined as positive.

### SFTSV genome sequencing and phylogenetic inference

SFTSV-positive samples were subjected to nested RT-PCR amplification using primer pairs as previously described [18] (Table S1). Nested RT-PCR was performed using the PrimeScript One Step RT-PCR Kit (Cat. No. RR055A) for the first round and the Premix Taq^™^ Kit (Cat. No. RR902A) for the second round (both from TaKaRa Bio Inc., Kusatsu, Japan). Following the respective initial steps (50 °C for 30 min and 94 °C for 2 min for round one; 94 °C for 5 min for round two), both rounds utilized 30 cycles of 94 °C for 45 s, 56 °C for 45 s, and 72 °C for 90 s, concluding with a 10-min extension at 72 °C. All amplification reactions were carried out using a Biometra TOne PCR System (Analytik Jena, Jena, Germany). Amplicons were purified using a QIAquick PCR Purification Kit (Qiagen, Hilden, Germany) and sequenced bidirectionally on an ABI 3730xl DNA Analyzer (Applied Biosystems, Foster City, CA, USA). The contigs were assembled using SeqMan Pro v7.1.0 (DNASTAR, Inc., Madison, WI, USA). Among the 131 patients in the Zhejiang cohort, 80 were successfully sequenced. Statistical analysis confirmed that this sequenced subset showed no significant demographic or clinical deviation from the overall cohort (*P* > 0.05; Table S2). The sequences have been deposited in GenBank [19].

For this analysis, a global dataset of SFTSV genomes was retrieved from GenBank on November 10, 2025. Sequences were filtered to exclude patent- or laboratory- derived samples, those with > 5% ambiguous bases, or those showing < 90% coverage relative to reference lengths (L: 6368 bp; M: 3378 bp; S: 1744 bp). This pipeline yielded a final curated dataset of 1820 high-quality genomes. Multiple sequence alignments were performed for each genomic segment using MAFFT v7.407 (Osaka University, Osaka, Japan) [27], followed by trimming of poorly aligned regions using TrimAl v1.4 (Centre for Genomic Regulation, Barcelona, Spain) [28]. Maximum-likelihood phylogenetic trees were constructed using IQ-TREE v1.6.12 (Medical University of Vienna, Vienna, Austria) [29] under best-fit substitution models (GTR+F+R for the L and M segments and TVMe+R for the S segment). Branch support was assessed using 1000 ultrafast bootstrap replicates. The resulting trees were visualized using iTOL (European Molecular Biology Laboratory, Heidelberg, Germany; https://itol.embl.de/) [30]. Reference sequences were retrieved from the National Center for Biotechnology Information database (GenBank accession numbers: NC_018136–NC_018138). The S-segment phylogeny served as a reference for genotyping, with major clades designated as genotypes A–E [16]. Multidimensional scaling (MDS) was employed to examine genomic clustering patterns and validate the robustness of this genotype classification [31, 32]. Hierarchical clustering was subsequently performed on the relative proportions of genotypes across different regions using the Euclidean distance metric and Ward's minimum variance method to identify regions with similar genotype compositions. Population differentiation was assessed using pairwise fixation index (*F*_*ST*_) via Weir & Cockerham’s method. Isolation-by-distance (IBD) was evaluated using the Mantel test by regressing Rousset's genetic distance *F*_*ST*_/(1*－F*_*ST*_) against the Haversine geographic distance matrix. Stratified linear regression and Mantel tests were performed using the R packages *hierfstat* and *vegan* to evaluate the spatial dynamics within Zhejiang province.

### Detection of reassortment and recombination

The potential reassortant strains were identified by comparing the genotypes assigned to the L, M, and S segments of each strain. Strains in which the three segments were assigned to different genotypes were flagged as putative reassortants. Recombination events within the SFTSV genomes were detected using the RDP4 software suite (University of Cape Town, Cape Town, South Africa) [33], which implements seven distinct algorithms (RDP, GENECONV, Bootscan, Maxchi, Chimaera, Siscan, and 3Seq). A recombination event was considered credible only if it was independently detected by at least four of the seven methods, with a statistical threshold of *P* < 10^−6^.

### Spatiotemporal phylogeographic inference

To elucidate the role of Zhejiang province of China in maritime transmission, we performed Bayesian phylogenetic analyses using BEAST v1.10.4 (University of Edinburgh, Edinburgh, UK) [34]. Given its structural stability and minimal recombination, the S segment was selected for discrete phylogeographic inference [16]. The recombinant sequences were removed to ensure reliable estimates. To mitigate sampling bias, we downsampled the 496 S-segment sequences from Japan, the Republic of Korea, and Zhejiang province of China to a representative subset of 207 sequences based on collection year, location, and genotype [35–37] (Fig. S1). Sequences from Zhejiang province were further partitioned by city (Hangzhou, Huzhou, Taizhou, and Ningbo) and islands (Zhoushan, Daishan, and Shengsi) to capture fine-scale spatiotemporal dynamics. Root-to-tip regression analysis was performed using TempEst v1.5.3 [38] (University of Edinburgh, Edinburgh, UK) to detect temporal signals (Fig. S2). The evolutionary model employed a GTR + Γ + I substitution model and a log-normal relaxed molecular clock. An asymmetric substitution model with the Bayesian Stochastic Search Variable Selection option was used in BEAST to infer the significant diffusion pathways between locations [39]. Bayes factor (BF > 3) and posterior probability (*PP* > 0.50) were used to quantify the support for these transitions [40]. Five independent Markov chain Monte Carlo runs of 100 million generations were performed by sampling every 10,000 steps. After discarding the 10% burn-in, the runs were combined using LogCombiner v1.10.4 in the BEAST package. Convergence and effective sample sizes (ESSs > 200) were assessed using Tracer v1.7.4 [41]. A maximum clade credibility (MCC) tree was generated using TreeAnnotator v1.10.4 in BEAST package, with internal nodes and the root annotated with their most probable ancestral geographic locations and corresponding marginal posterior probabilities. The annotated tree was visualized using iTOL and spatiotemporal diffusion patterns were reconstructed using SpreaD3 (KU Leuven, Leuven, Belgium) [42].

### Construction of host-location interaction network

We developed two complementary network models by integrating the host data from a comprehensive meta-analysis by Cui et al. [6] and metadata from GenBank. Host data were defined by the presence of SFTSV RNA or antibodies. First, a radial hierarchical network was developed to visualize the taxonomic relationships of SFTSV hosts, with nodes organized in concentric layers representing the virus (central node), class, order, and family taxa. Edges in this model represent hierarchical parent–child relationships, with node sizes scaled according to taxonomic rank. Second, a bipartite host-location interaction network was constructed to assess the regional breadth of SFTSV associations. This network consisted of two distinct node sets representing the host species (circles) and geographical sampling locations (triangles), with undirected edges indicating documented associations in which the SFTSV genomes were identified in a specific host at a given location. The bipartite architecture was visualized using the R package *igraph*.

### Mutation-phenotype association and evolutionary pressure

We identified genotype B-specific markers as residues present in > 90% of genotype B sequences but < 10% of sequences from other genotypes. We performed a genetic association study using a curated set of 774 SFTSV genomes with annotated patient outcomes [18]. Multivariable logistic regression was used to estimate the odds ratios (*OR*) and 95% confidence intervals (*CI*) for the association between individual mutations and fatal outcomes, adjusted for age, sex, and symptom onset-to-admission interval. The Benjamini–Hochberg false discovery rate correction was applied to each gene to control for multiple testing.

For evolutionary pressure analysis, we first generated codon-aware multiple sequence alignments for each major viral protein (RdRp, Gn, Gc, NSs, and NP) from 774 outcome-annotated genomes, using the Codon-MSA pipeline (Temple University, Philadelphia, PA, USA; https://github.com/veg/hyphy-analyses/blob/master/codon-msa). Recombinant strains and sequences containing premature stop codons were excluded from the analysis. The redundant nucleotide sequences linked to identical clinical outcomes collapsed, yielding the final analytical datasets of 728 (RdRp), 615 (Gn), 596 (Gc), 509 (NSs), and 393 (NP) sequences. Positive selection was assessed using four complementary algorithms (FEL, MEME, SLAC, and FUBAR) implemented in HyPhy v2.5.24 (Temple University, Philadelphia, PA, USA) [43]. A site was defined as under robust positive selection if it was identified by at least two methods (*P* < 0.05 or posterior probability > 0.90). The ratio of non-synonymous (*dN*) to synonymous (*dS*) substitution rates was calculated to estimate selective pressure. Differential selection pressure between genotype B (foreground) and other genotypes (background) was evaluated using BUSTED-PH [44, 45], and site-specific differences were identified using contrast-FEL [46]. Finally, positively selected sites, genotype-specific markers, and mortality-associated mutations were mapped onto established SFTSV protein domain architectures [47, 48].

### Meteorological data source and statistical analysis

Xinyang city of Henan province (inland region) and Zhoushan city of Zhejiang province (coastal region) were selected as representative sites to assess the potential impact of meteorological factors on SFTS incidence in China. Monthly average atmospheric temperature (°C), precipitation (mm), and sunshine duration (h) between 2012 and 2018 were derived from the China Meteorological Data Service Center (http://data.cma.cn). A one-month time lag was incorporated into the analysis to account for the observed synchronization between climatic shifts and disease outbreaks. This was based on the preliminary finding that the incidence of SFTS consistently lagged one month after the initial rise in ambient temperature, reflecting the ecological interval required for vector activation and viral transmission. We employed generalized additive models (GAM) to capture the potential nonlinear and threshold-dependent relationships between environmental drivers and SFTS risk [49, 50]. The model was structured as log(E[Y*ₜ*]) = *α* + s_1_(Temperature*ₜ*₋₁) + s_2_(Precipitation*ₜ*₋₁) + s_3_(Sunshine*ₜ*₋₁) + *γ* · Year*ₜ*, where E[Y*ₜ*] represents the expected monthly SFTS incidence at month *t*, *α* represents the baseline log-transformed incidence rate of SFTS across the study period, *t*−1 represents a one-month time lag, and *γ* denotes the vector of coefficients for the categorical variable “Year”. Thin-plate regression splines, denoted as s_*i*_, were used to apply smoothing functions to the meteorological predictors. The variable “Year” was included as a categorical factor to control long-term interannual trends and unobserved secular variations. The adequacy of the basis dimension (*k*) for each smoothing term was verified through diagnostic checking. To visualize the exposure–response relationships, smoothing estimates (s_*i*_) were exponentiated to calculate the relative risks (*RR*). Model performance was evaluated using the percentage of the total deviance explained.

All statistical analyses were performed using R v4.3.3 (R Foundation for Statistical Computing, Vienna, Austria) and statistical significance was defined as two-sided *P* < 0.05.

### Ethics approval and consent to participate

This study was approved by the Ethics Committee of the Second Military Medical University, and written informed consent was obtained from all patients or their guardians.

## Results

### Geographically decoupled epidemiology: inland-high-incidence vs. coastal-high-fatality

Between 2010 and 2023, 27,457 SFTS cases and 1326 deaths were reported across 27 provinces and municipalities in Chinese mainland. Significant spatial heterogeneity was observed, with 99.05% (27,197/27,457) of incident cases and 99.62% (1321/1326) of deaths concentrated within seven provinces: Henan, Hubei, and Anhui, Zhejiang, Jiangsu, Shandong, and Liaoning. A comparison between inland regions (Henan, Hubei, and Anhui) and coastal regions (Zhejiang, Jiangsu, Shandong, and Liaoning) revealed a geographically decoupled pattern of SFTS incidence and fatality (Table 1). The inland provinces demonstrated a significantly higher cumulative incidence rate compared to the coastal provinces (7.31 vs. 3.84 per 100,000). Using the inland region as the reference, the *RR* for incidence in the coastal region was 0.52 (95% *CI:* 0.51–0.54, *P* < 0.001). In contrast, the CFR showed the opposite trend, being significantly lower in the inland regions than in the coastal regions (2.61% vs. 8.04%), with coastal regions showing a 3.08-fold higher risk of mortality (95% *CI:* 2.75–3.45, *P* < 0.001). At the provincial level, this pronounced divergence was further illustrated by comparing Henan (as a representative inland province) and Zhejiang (as a representative coastal province). The cumulative incidence of SFTS was significantly lower in Zhejiang than in Henan (1.53 vs. 6.33 per 100,000; *RR* = 0.24, 95% *CI:* 0.23–0.26, *P* < 0.001). Conversely, the CFR was significantly higher in Zhejiang than in Henan (12.53% vs. 2.04%), representing a significantly elevated fatality risk in Zhejiang (*RR* = 6.15, 95% *CI:* 4.85–7.80, *P* < 0.001).

**Table 1:** Comparison of cumulative incidence and case fatality rate between regions and provinces

Characteristic	Total population	Cumulative incidence				CFR			
		Cases	Rate (per 100,000)	*RR* (95% *CI*)	*P* value	Deaths	Rate (%)	*RR* (95% *CI*)	*P* value
Region									
Inland (Ref)	218,145,247	15,942	7.31	1.00 (Reference)	–	416	2.61	1.00 (Reference)	–
Coastal	293,434,464	11,255	3.84	0.52 (0.51–0.54)	< 0.001	905	8.04	3.08 (2.75–3.45)	< 0.001
Province									
Henan (Ref)	99,365,519	6286	6.33	1.00 (Reference)	–	128	2.04	1.00 (Reference)	–
Zhejiang	64,567,588	990	1.53	0.24 (0.23–0.26)	< 0.001	124	12.53	6.15 (4.85–7.80)	< 0.001

Inland regions included Henan, Hubei, and Anhui provinces. Coastal regions included Zhejiang, Jiangsu, Shandong, and Liaoning provinces. *P* values were calculated using Pearson’s Chi-square test. *CFR* case fatality rate, *RR* relative risk, *CI* confidence interval, *Ref* reference group

**Fig. 1:**
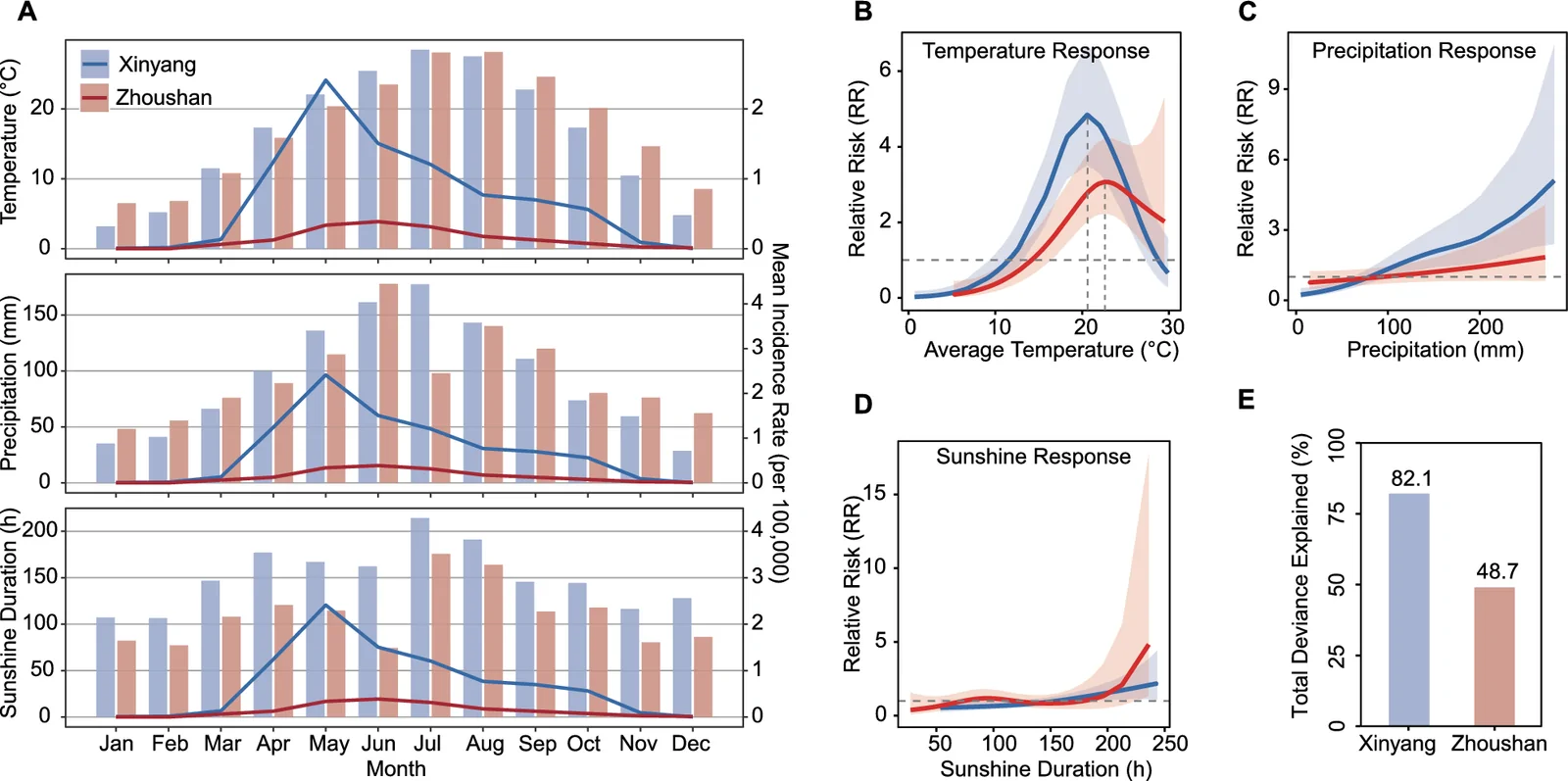
Spatiotemporal distribution of SFTS incidence and its association with meteorological factors in Xinyang (inland) and Zhoushan (coastal). **A** Monthly distribution of meteorological factors and severe fever with thrombocytopenia syndrome (SFTS) incidence. Bar charts represent the monthly mean temperature (°C, top), precipitation (mm, middle), and sunshine duration (h, bottom) for Xinyang (blue) and Zhoushan (red). The overlaid line plots show the corresponding mean incidence rate per 100,000 population (right y-axis) in each city. **B**–**D** Exposure–response curves of meteorological drivers. The non-linear associations between SFTS relative risk (*RR*) and B average temperature, C precipitation, and D sunshine duration. Solid lines indicate the estimated *RR*, and shaded areas represent the 95% confidence intervals. The horizontal dashed lines denote an *RR* of 1 (reference value). **E** Total deviance explained. The bar chart indicates the percentage of total deviance in SFTS incidence explained by the combined meteorological driving factors in Xinyang and Zhoushan

We employed an integrated analysis of the case incidence and meteorological factors to explore the drivers associated with the incidence of SFTS (Fig. 1A and Fig. S3). In Xinyang city of Henan province, the incidence of SFTS began in March, rose sharply, peaked in May, and consistently lagged the initial temperature rise by one month. In Zhoushan city of Zhejiang province, the peak was attenuated and occurred slightly later, reflecting the potential moderating influence of differing climate on the activity window of the vector. GAM analysis with a one-month lag identified potent nonlinear associations between environmental drivers and SFTS incidence (Fig. 1B–D, Fig. S4 and Table S3). Temperature emerged as the primary determinant, with the *RR* escalating significantly once the temperature exceeded the 10 °C threshold (Fig. 1B). Notably, Xinyang exhibited a sharper response and higher peak sensitivity (*RR* = 4.85; 95% *CI:* 3.52–6.69 at 20.6 °C) compared to Zhoushan (*RR* = 3.07; 95% *CI:* 2.23–4.22 at 22.6 °C), indicating increased climatic vulnerability in the inland region. Precipitation was positively associated with the risk of SFTS and was markedly more pronounced in inland Xinyang than in coastal Zhoushan (Fig. 1C). Furthermore, the sunshine duration displayed a distinctive threshold effect unique to the coastal region, where the SFTSV transmission risk remained stable until monthly sunshine exposure exceeded 200 h/month, after which the *RR* increased precipitously (Fig. 1D). The model explained 82.1% and 48.7% of the total deviance in Xinyang and Zhoushan, respectively (Fig. 1E).

### Distinct geographic distribution of viral lineages in inland and coastal regions

**Fig. 2:**
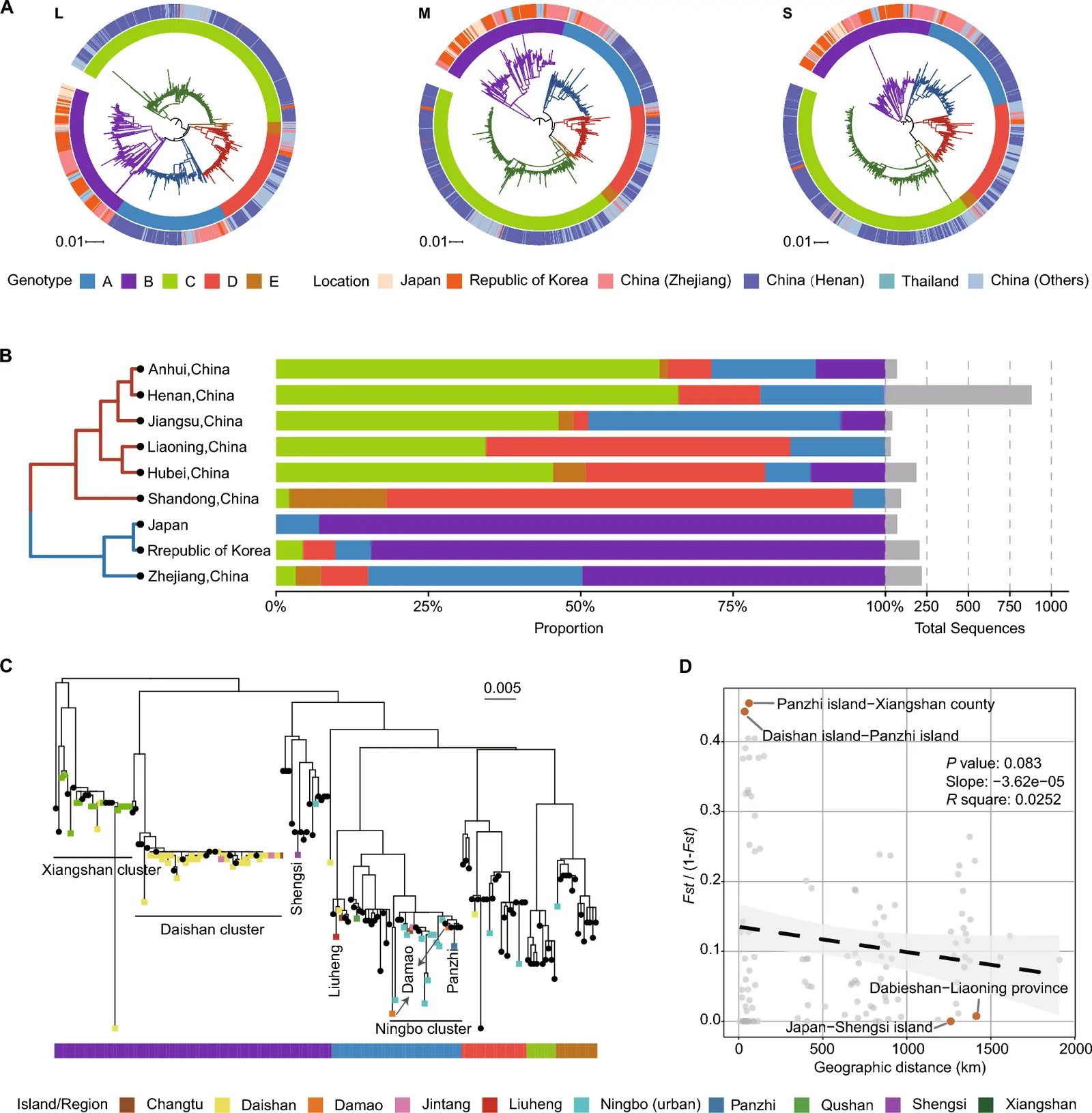
Phylogeny and geographic distribution of SFTSV genotypes. **A** Maximum-likelihood phylogenetic inference of the Large (L), Medium (M), and Small (S) segments based on 1820 severe fever with thrombocytopenia syndrome virus (SFTSV) genomes. Branch colors and the inner rings denote genotypes A–E, while the outer rings represent sampling locations across East Asia (Japan, the Republic of Korea, Thailand, and various Chinese provinces). **B** Hierarchical clustering of regions based on the compositional similarity of genotypes (left), aligned with the relative proportion of genotypes A–E (middle) and the absolute number of total sequences (right). Dendrogram branches are colored to indicate the two major clusters (red and blue clades) identified by the analysis. **C** Detailed evolutionary structure within the Zhejiang coastal-island network. The maximum-likelihood tree reveals significant inter-island genetic heterogeneity, highlighting major localized clusters (e.g., Xiangshan, Daishan, and Ningbo clusters). Colored terminal nodes and the bottom annotation bar indicate specific island or regional origins. **D** Isolation-by-distance (IBD) analysis demonstrating the decoupling of genetic differentiation and geographic distance. The scatter plot illustrates the relationship between pairwise genetic differentiation *F*_*ST*_/(1-*F*_*ST*_) and geographic distance (km). The dashed line represents the linear regression fit with the shaded area indicating the 95% confidence interval, while annotated orange dots highlight specific location pairs

A total of 1820 SFTSV genomes from China, Japan, the Republic of Korea, and Thailand were curated in this study (sequences are listed in Table S4). Phylogenetic trees reconstructed from the L, M, and S segments were largely concordant with strains segregating into genotypes A–E (Fig. 2A). MDS analysis consistently resolved five well-separated clusters corresponding to genotypes A–E, thereby supporting the robustness of this classification (Fig. S5).

The spatial distribution of viral genotypes exhibited distinct regional patterns. Multiple genotypes co-circulated in China, whereas Japan, the Republic of Korea, and Thailand were dominated by genotype B (Fig. S6). Hierarchical clustering based on genotype composition categorized the nine regions into two major clades (Fig. 2B). The first clade (highlighted in red) primarily comprised central and eastern Chinese provinces, including Anhui, Henan, and Hubei provinces. This clade was predominantly characterized by a high prevalence of genotype C, alongside varying proportions of genotypes A and D. In contrast, the second clade (highlighted in blue) consisted of Japan, the Republic of Korea, and China's Zhejiang province, where genotypes B was the dominant circulating lineage.

Focusing on fine-scale dynamics within Zhejiang province, the phylogenetic reconstruction revealed striking spatial heterogeneity across coastal and archipelagic environments (Fig. 2C). Despite administrative proximity, Xiangshan county and Ningbo urban center of Zhejiang province exhibited distinct evolutionary trajectories. Constrained by its peninsular geography and seaport status, Xiangshan county harbored a tightly confined genotype B subclade. In contrast, the Ningbo urban area displayed greater genetic diversity (genotypes A, B, and C), showing a closer affinity to inland lineages. This complex genetic mosaic extends to the Zhoushan archipelago. Shengsi island harbored a genotype B sublineage genetically closer to Japanese strains (e.g., LC536543) than to those on neighboring islands (Fig. S7). Conversely, the adjacent Qushan island was dominated by genotype A strains aligned with the Shandong lineages (e.g., MT114292). IBD analysis confirmed the nonlinear nature of these dynamics, yielding a negligible spatial correlation (*R*^2^ = 0.0252, *P* = 0.083) and rejecting the traditional stepping-stone diffusion model (Fig. 2D). For example, the Panzhi-Xiangshan pair exhibited extreme genetic differentiation despite geographic proximity (< 100 km), whereas the Japan-Shengsi (China) island pair maintained a similar genetic distance across > 1200 km.

### Zhejiang province exhibits higher recombination frequencies than inland regions

**Fig. 3:**
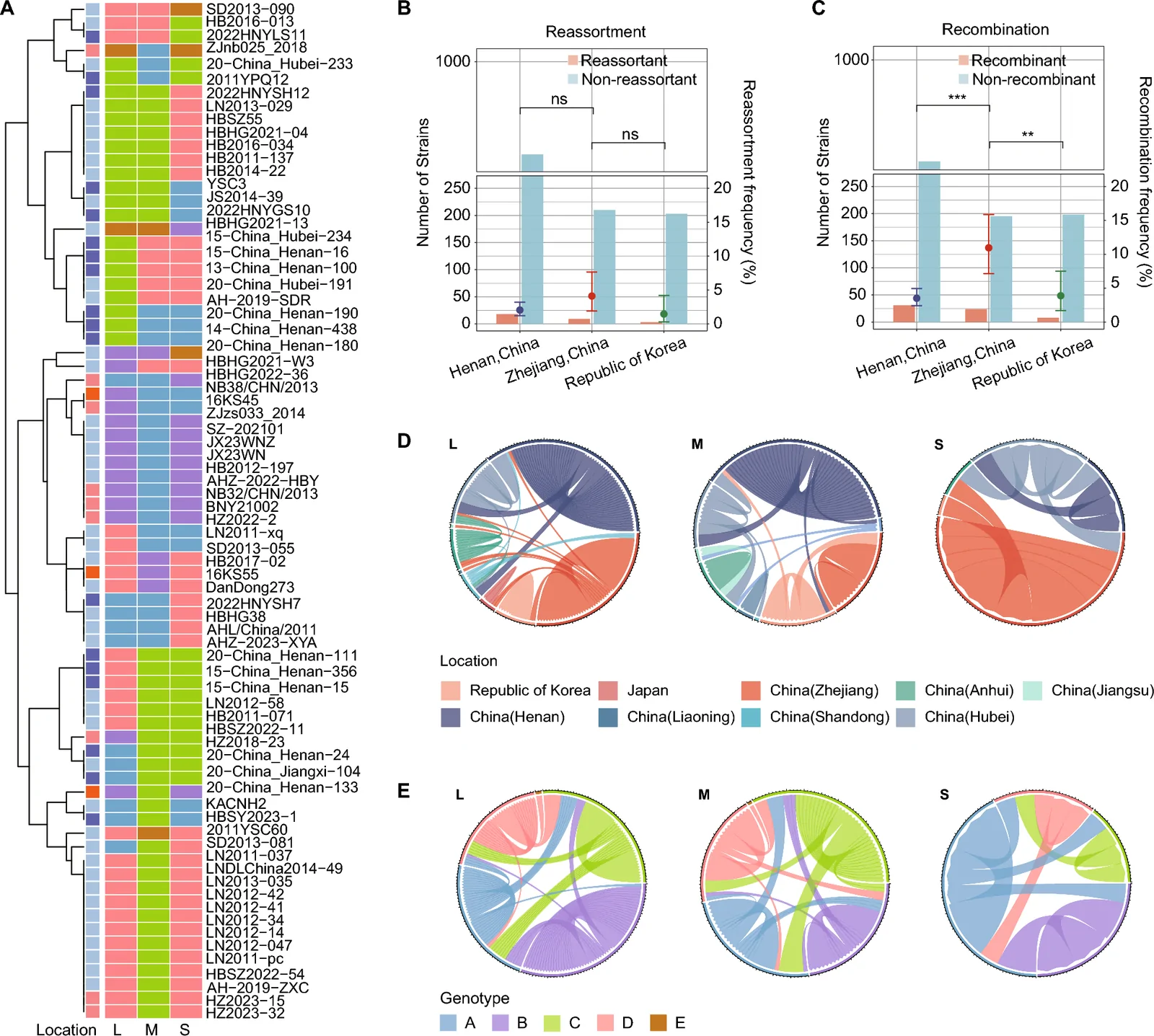
Genomic reassortment and recombination dynamics of SFTSV. **A** Genotypic constellations and reassortment patterns. Each row in the heatmap represents an individual viral strain, clustered by genetic similarity (left tree). Columns denote the sampling location and the respective genotypes (A–E) of the Large (L), Medium (M), and Small (S) segments. **B** and **C** Genomic reassortment (B) and recombination (C) frequencies across key regions. Bar plots (left y-axis) display the absolute number of strains. Colored dots with error bars (right y-axis) indicate the estimated frequency (%) of reassortment or recombination. (*ns* non-significant; **, *P* < 0.01; ***, *P* < 0.001). **D** and **E** Directional genetic exchanges via recombination. Chord diagrams map the parental origins of recombinant strains across the three genomic segments, categorized by sampling location (D) and genotype (E). Link thickness reflects the frequency of recombination events, with arrows indicating the directionality of genetic flow from parental lineages to descendants. In D, warm colors (e.g., red, pink) highlight coastal and maritime regions (Zhejiang province of China, the Republic of Korea, Japan), whereas cool colors (e.g., blue, green) represent inland Chinese provinces

Based on the phylogenetic incongruence across genomic segments, we identified 74 reassortant strains, accounting for 4.1% of the 1820 isolates (Fig. 3A and Table S5). A higher reassortment frequency was observed in China’s Zhejiang province (4.1%, 9/219) than in China’s Henan province (2.0%, 18/888) and the Republic of Korea (1.5%, 3/206), although the differences were not statistically significant (Fig. 3B). The reassortment patterns largely reflected the locally dominant genotypes. In Zhejiang province, the predominant reassortment configurations across the L, M, and S segments were B-A-B (3/9) and D-C-D (2/9). In Henan province, the reassortment strains exhibited a mixture of genotypes A, C, and D, with patterns D-C-C, C-A-A, and C-D-D each accounting for 3 out of the 18 strains. Reassortants from the Republic of Korea exhibited patterns D-B-D, B-C-B, and B-A-A. (Fig. 3A).

Recombination events were detected in 48 (2.6%), 33 (1.8%), and 7 (0.4%) isolates in the L, M, and S segments, respectively (Table S6). A significantly higher recombination frequency was observed in China’s Zhejiang province (24/219, 11.0%) than in China’s Henan province (31/881, 3.5%) and the Republic of Korea (8/206, 3.9%) (both *P* < 0.01; Fig. 3C). Chord diagrams summarizing the parental origins of the recombinant isolates per segment revealed distinct patterns (Fig. 3D). For the L segment, recombinants from Zhejiang province were derived from local and distant parental sequences, including strains from the Republic of Korea, Japan, and multiple Chinese provinces, highlighting the role of this region as a hub for genetic exchange. The parental strains of the M segment were predominantly of local and Republic of Korea origin, whereas those of the S segment included local, Republic of Korea, and Anhui province of China strains. Extensive inter‑genotype exchanges were observed across all segments (Fig. 3E).

### Phylogeographic inference suggests plausible maritime transmission networks

**Fig. 4:**
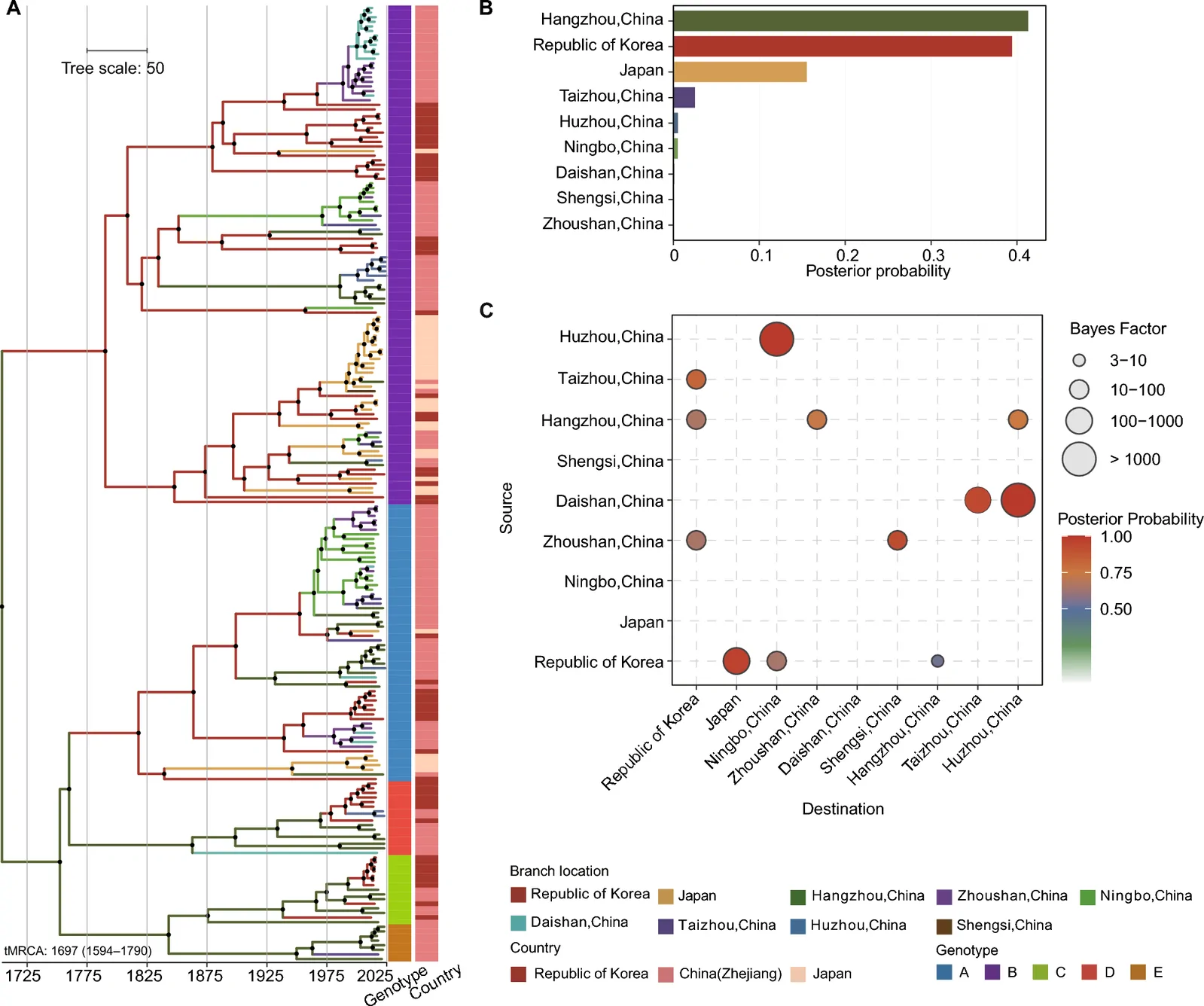
Discrete phylogeographic analysis of SFTSV across Zhejiang province of China, the Republic of Korea, and Japan. **A** Time-scaled maximum clade credibility (MCC) tree. The phylogeny was reconstructed using the Small (S) segment of 207 severe fever with thrombocytopenia syndrome virus (SFTSV) strains. Branch colors indicate inferred ancestral locations. The right-side annotation bars represent the genotypes (A–E) and country of origin for each terminal node. The bottom axis represents calendar years. **B** Root ancestral state probabilities. The horizontal bar chart illustrates the posterior probabilities for the most likely geographic origin of the tree root, with China (Hangzhou), the Republic of Korea, and Japan showing the highest statistical support. **C** Inferred viral migration pathways. The bubble matrix illustrates significant spatial transitions between locations. The y-axis and x-axis represent the source and destination locations, respectively. Bubble sizes indicate the Bayes factor support for the transition (categorized into intervals: 3–10, 10–100, 100–1000, and >1000), while the color gradient represents the posterior probability (ranging from 0.50 to 1.00). Only pathways with substantial statistical support (Bayes factor ≥ 3 and posterior probability ≥ 0.5) are displayed

To reconstruct the spatiotemporal dynamics of SFTSV in Zhejiang province of China, the Republic of Korea, and Japan, we performed a discrete Bayesian phylogeographic analysis incorporating sequences from these locations (sequences are listed in Table S7). The resulting Maximum Clade Credibility (MCC) tree estimated the time to the most recent common ancestor (tMRCA) to be approximately 1697 (95% highest posterior density [*HPD*]: 1594–1790; Fig. 4A). The mean evolutionary rate for the S segment was estimated at 9.37 × 10⁻^5^ substitutions/site/year (95% *HPD*: 8.23 × 10⁻^5^–1.00 × 10⁻^4^). Ancestral state reconstruction suggested polyphyletic origins, with Hangzhou city in Zhejiang province, China (*PP* = 0.41), the Republic of Korea (*PP* = 0.39), and Japan (*PP* = 0.16) inferred as the most probable ancestral locations (Fig. 4B). Spatial transition matrix revealed a highly interconnected regional network (Fig. 4C). At the international level, we identified strongly supported unidirectional dispersal pathways from the Republic of Korea to Japan as well as to coastal and inland cities in Zhejiang province, China (Ningbo and Hangzhou). Concurrently, multiple locations within Zhejiang province (Taizhou, Hangzhou, and Zhoushan island) were identified as source populations seeding viral lineages to Republic of Korea. At the sub-regional level within Zhejiang province, we identified frequent viral exchanges across distinct ecological zones (Fig. 4C). Specifically, six significant viral transmission pathways were identified: dispersal from the offshore island of Daishan island to the coastal city of Taizhou and the inland city of Huzhou; intra-archipelago transmission from Zhoushan island to Shengsi island; and outward dispersal from the inland city of Hangzhou to both Zhoushan island and Huzhou city. Additionally, a transmission route was observed from Huzhou to the coastal city of Ningbo. These bidirectional and cross-regional pathways indicate a complex transmission network among offshore islands, coastal areas, and further-inland regions.

### Host-location networks and the ecological context of regional transmission

**Fig. 5:**
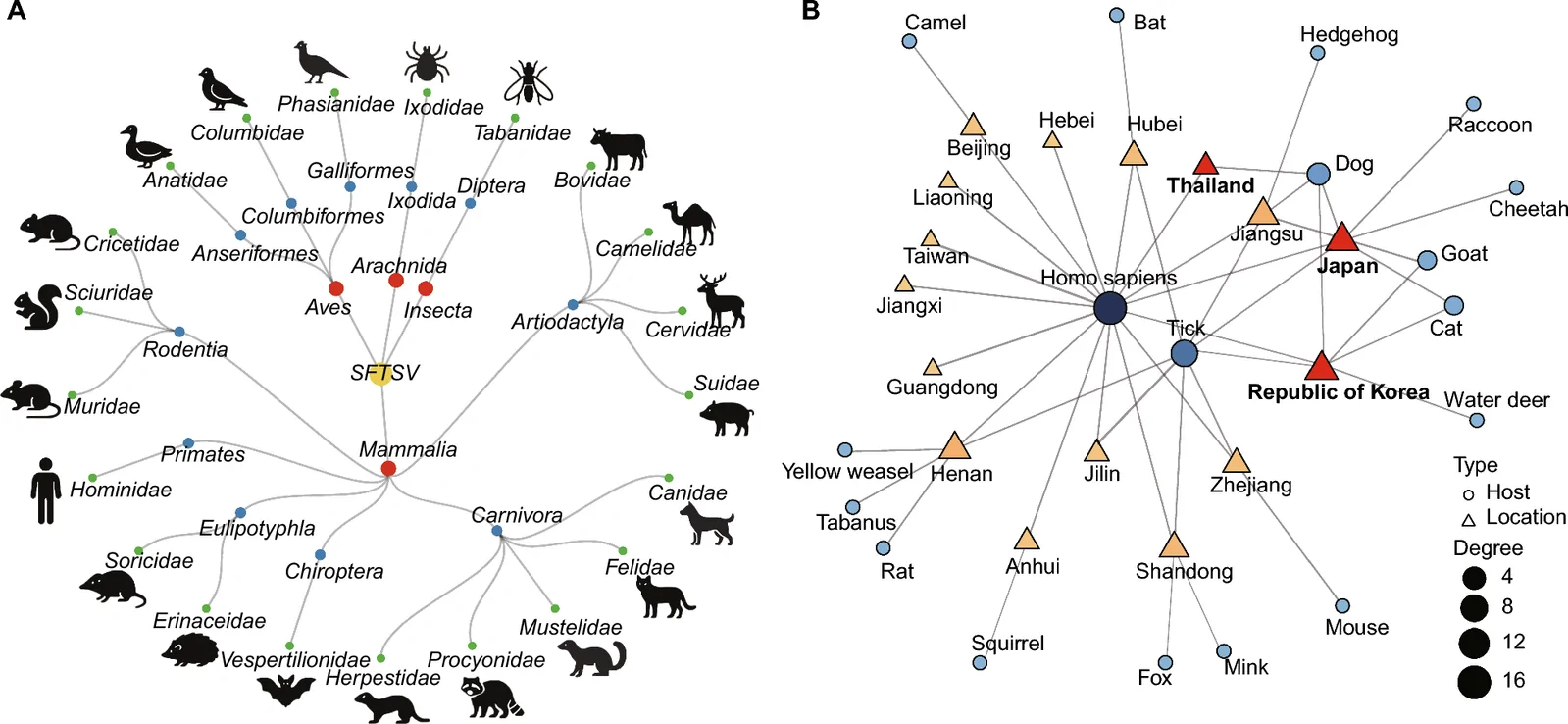
Taxonomic diversity and geographic distribution of SFTSV hosts. **A** Radial taxonomic hierarchy of severe fever with thrombocytopenia syndrome virus (SFTSV) reservoirs. The network illustrates the phylogenetic organization of documented hosts. The central node represents SFTSV (yellow), with subsequent layers radiating outward to denote host Classes (red), Orders (blue), and Families (green). Edges define the hierarchical linkages from the virus to terminal host families, which are annotated with representative animal silhouettes. Taxonomic data were curated from Cui et al. [6] and GenBank metadata. **B** Geographic-host association network. The bipartite network depicts documented connections between sampling locations (triangles) and host species (circles) associated with SFTSV genomic records. Orange triangles represent Chinese provinces, while red triangles denote countries. Edges indicate confirmed viral detection within a specific host at a given location. Node size is directly proportional to the degree centrality (number of connections), prominently highlighting *Homo sapiens* and ticks as the central nodes

A radial hierarchical network illustrated the broad phylogenetic breadth of SFTSV hosts spanning four classes (*Arachnida*, *Insecta*, *Mammalia*, and *Aves*), along with their constituent orders and families (Fig. 5A, Table S8). This underscores the capacity of the virus to infect both invertebrate vectors (e.g., ticks and insects) and diverse arrays of vertebrate hosts. A bipartite host-location interaction network (Fig. 5B) identified *Homo sapiens* (*n* = 16) and ticks (*n* = 8) as the most highly connected nodes. Numerous other mammals (dogs, goats, cats, and wildlife) have also been documented. Marked geographic heterogeneity was evident in the documented host diversity. Despite contributing fewer sequences overall, Japan (comprising eight host types) and the Republic of Korea (comprising six host types) exhibited the highest host diversity. In contrast, most Chinese provinces showed limited documented host diversity (e.g., Jiangsu and Henan, each *n* = 5; Shandong, *n* = 4). Notably, Zhejiang province, a key bridging region, exhibited relatively low documented host diversity (*n* = 3), which may reflect surveillance bias, differential sampling intensity, or genuine regional ecological variation.

### The high-fatality coastal phenotype is associated with the dominance of genotype B

**Fig. 6:**
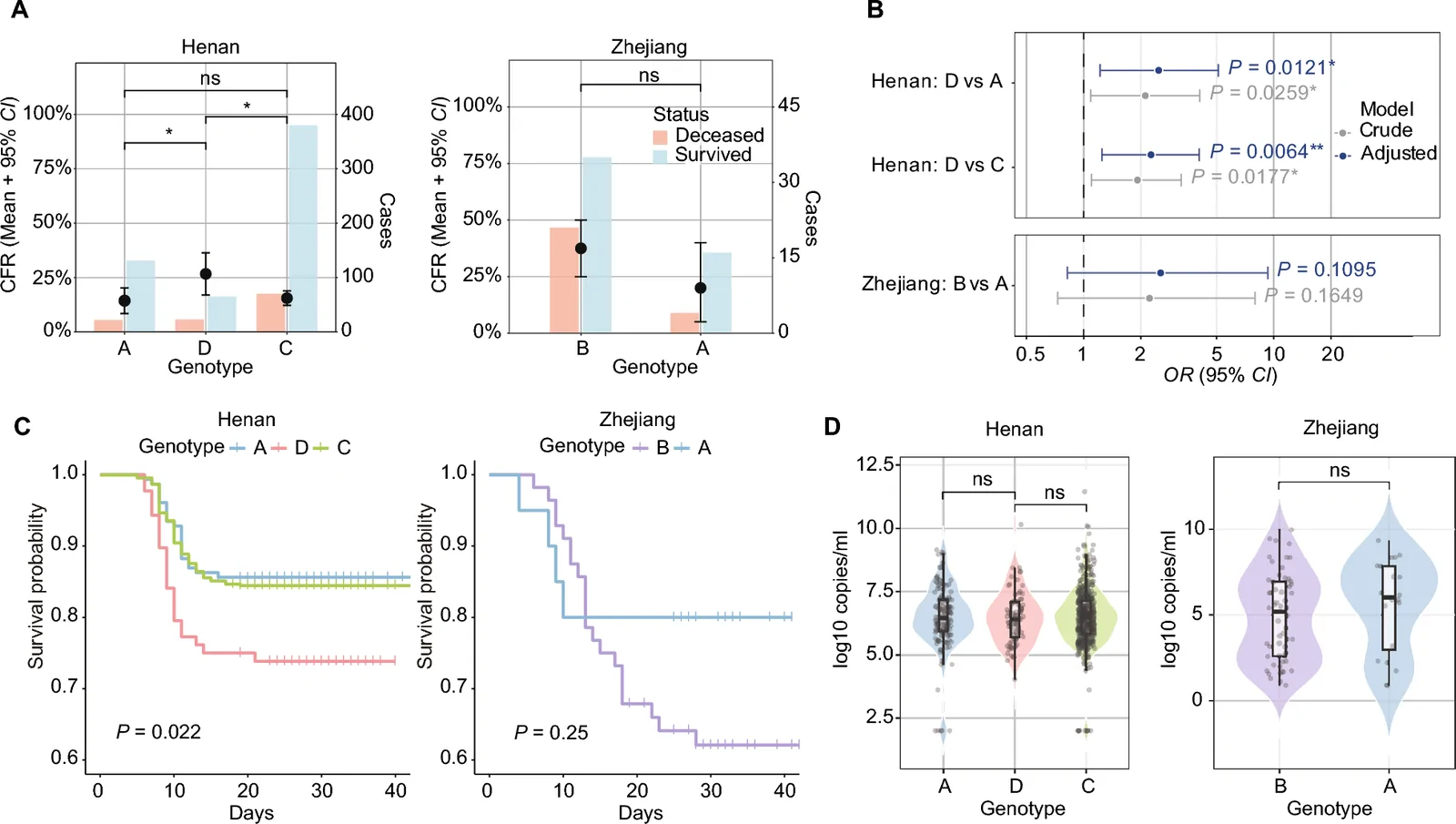
Genotype-specific clinical outcomes of SFTSV infection. **A** Case fatality rate (CFR) and genotype distribution. Mortality is compared across genotypes in the Henan (left) and Zhejiang (right) cohorts. Points and error bars indicate the mean CFR with 95% confidence intervals (*CI*). Statistical significance between groups was assessed using Fisher’s exact test (*, *P* < 0.05; ns, non-significant). **B** Risk assessment of genotype-linked mortality. Forest plots display the odds ratios (*OR*) for mortality risk comparing specific genotypes. Both crude (grey) and adjusted (blue) models are presented, with error bars denoting 95% *CI*s. *P* values are indicated alongside each model. **C** Kaplan–Meier survival analysis. Cumulative survival probabilities are stratified by genotype for the Henan and Zhejiang provinces. *P* values were derived from log-rank tests. **D** Comparison of viral loads. Violin plots superimposed with box plots illustrate the distribution of serum viral loads (${\mathrm{l}\mathrm{o}\mathrm{g}}_{10}$ copies/ml) across genotypes

A comparison of inland case cohorts from Henan (*n* = 805) and Zhejiang (*n* = 131) provinces revealed comparable age and sex distributions; however, there was a significantly longer symptom onset-hospitalization interval and a higher CFR in the Zhejiang cohort than in the Henan cohort (Zhejiang vs. Henan: 25.8% vs. 17.3%, *P* = 0.029; Table 2). In the Henan cohort, genotype D was associated with a significantly higher CFR (26.1%) than genotypes A (14.4%) and C (15.6%, both* P* < 0.05). In the Zhejiang cohort, genotype B showed a higher CFR (37.5%) than genotype A (20.0%), although this difference was not statistically significant, likely because of the limited sample size within this subgroup (Fig. 6A). Multivariate logistic regression confirmed that genotype D was an independent risk factor for CFR in the Henan cohort, whereas genotype B was associated with higher CFR in the Zhejiang cohort (Fig. 6B). Kaplan–Meier survival analysis revealed the lowest survival probability for genotype D in the Henan cohort (log-rank test, *P* = 0.022) and genotype B in the Zhejiang cohort (log-rank test, *P* = 0.25; Fig. 6C). No significant differences were observed in viral load (Fig. 6D) or laboratory indicators (Fig. S8) among the genotypes in the Henan and Zhejiang cohorts.

**Table 2:** Characteristics of the SFTS patients from Henan and Zhejiang provinces

Characteristics	Overall (*n* = 936)	Henan (*n* = 805)	Zhejiang (*n* = 131)	*P* value
Age, years (median [IQR])	65.00 (55.00, 72.00)	65.00 (54.00, 72.00)	65.00 (55.00, 74.00)	0.441
Male (%)	373 (39.9)	317 (39.4)	56 (42.7)	0.526
CFR (%)	172 (18.4)	139 (17.3)	33 (25.8)	0.029
Delay from onset to admission, days (median [IQR])	5.00 (4.00, 7.00)	5.00 (4.00, 7.00)	5.00 (4.00, 8.00)	0.042
Fever (%)	931 (99.5)	804 (99.9)	127 (96.9)	< 0.001
Chill (%)	134 (14.3)	95 (11.8)	39 (29.8)	< 0.001
Headache (%)	157 (16.8)	112 (13.9)	45 (34.4)	< 0.001
Fatigue (%)	877 (93.7)	759 (94.3)	118 (90.1)	0.100
Myalgia (%)	716 (76.5)	625 (77.6)	91 (69.5)	0.053
Anorexia (%)	716 (76.5)	654 (81.2)	62 (47.3)	< 0.001
Nausea (%)	611 (65.3)	609 (75.7)	2 (1.5)	< 0.001
Vomiting (%)	426 (45.5)	349 (43.4)	77 (58.8)	0.001
Abdominal pain (%)	169 (18.1)	90 (11.2)	79 (60.3)	< 0.001
Diarrhea (%)	307 (32.8)	255 (31.7)	52 (39.7)	0.087
Hemorrhage (%)	354 (37.8)	339 (42.1)	15 (11.5)	< 0.001
Lymphadenopathy (%)	504 (53.8)	458 (56.9)	46 (35.1)	< 0.001
ALT, U/L (median [IQR])	43.00 (26.00, 88.00)	41.00 (26.00, 84.25)	60.00 (33.50, 98.00)	0.050
AST, U/L (median [IQR])	100.00 (53.00, 228.75)	95.00 (53.00, 227.00)	122.00 (59.50, 239.00)	0.242
CK, U/L (median [IQR])	316.00 (145.00, 724.00)	325.50 (149.00, 723.75)	276.00 (121.50, 709.00)	0.135
creatinine, μmol/L (median [IQR])	78.00 (64.00, 97.00)	78.00 (64.00, 97.00)	77.00 (66.50, 96.50)	0.994
LDH, U/L (median [IQR])	429.10 (276.00, 740.00)	420.50 (277.25, 729.00)	476.00 (263.50, 747.50)	0.951
PLT, 10^9^/L (median [IQR])	63.00 (43.00, 84.00)	62.00 (43.00, 84.00)	63.00 (43.60, 85.50)	0.978
WBC, 10^9^/L (median [IQR])	2.10 (1.50, 3.30)	2.10 (1.50, 3.30)	2.10 (1.40, 3.38)	0.688
PWR (median [IQR])	29.11 (16.33, 44.44)	29.05 (16.28, 44.17)	30.00 (17.95, 45.51)	0.724
Viral load, logged, copies/ml (median [IQR])	6.40 (5.69, 7.14)	6.43 (5.80, 7.10)	6.19 (3.27, 7.29)	0.014

Data are presented as median (IQR) and *n* (%), and *P* values were compared using the chi-square test, Fisher’s exact test, or Kruskal–Wallis test. IQR, interquartile range. *SFTS* severe fever with thrombocytopenia syndrome, *IQR* interquartile range, *ALT* alanine aminotransferase, *AST* aspartate aminotransferase, *CK* creatine kinase, *Cr* creatinine, *LDH* lactate dehydrogenase, *PLT* platelet, *WBC* white blood cell, *PWR* PLT-WBC ratio

### Selection pressure and candidate molecular markers of SFTSV genotypes

**Fig. 7:**
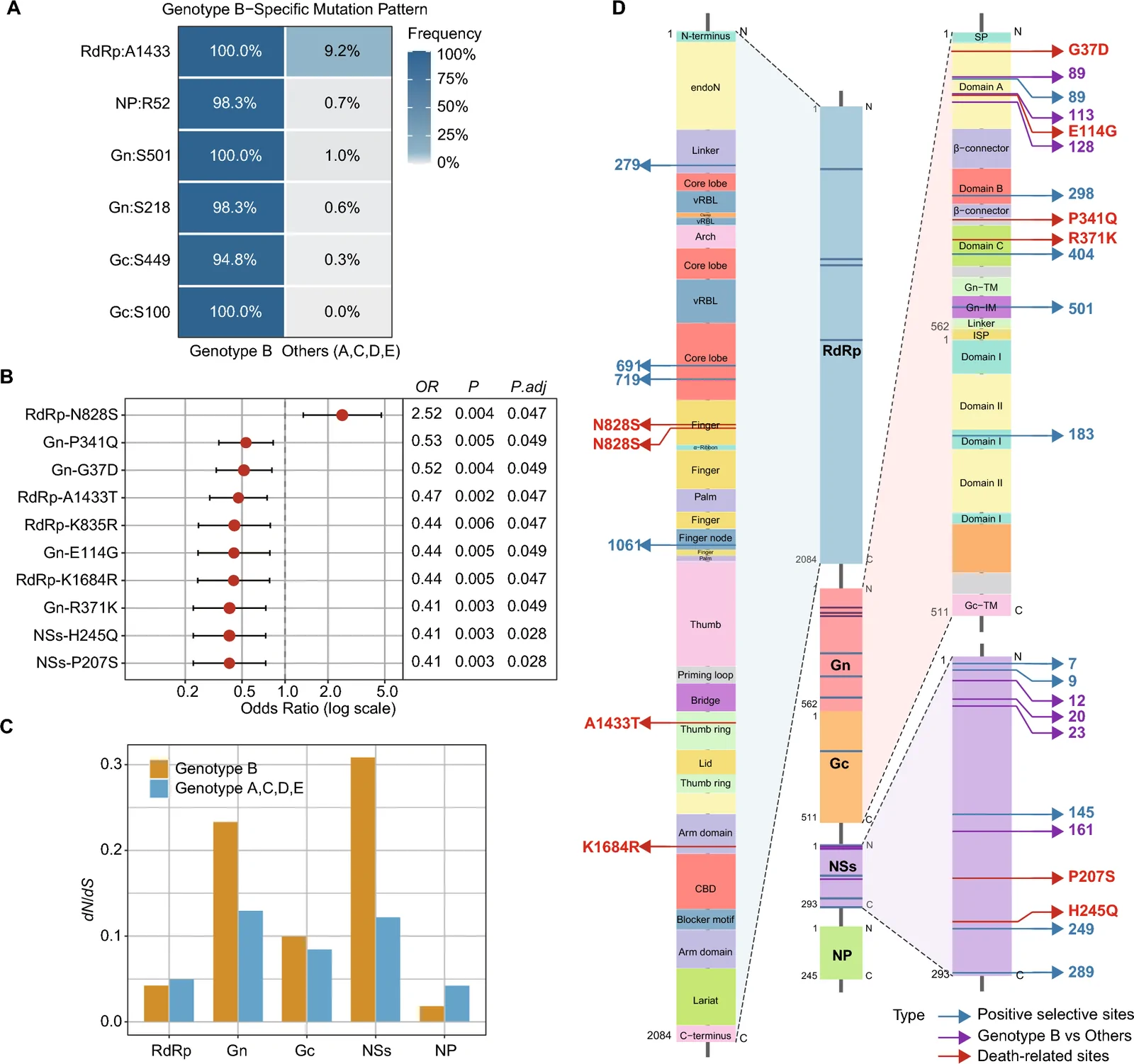
Evolutionary selection and molecular determinants of SFTSV clinical severity. **A** Genotype B-specific mutational signature. The heatmap displays the frequency of amino acid substitutions that distinguish genotype B from other genotypes (A, C, D, and E). **B** Association between amino acid substitutions and fatal outcomes. The forest plot illustrates the odds ratios (*OR*, plotted on a log scale) for substitutions significantly correlated with clinical mortality. Error bars represent 95% confidence intervals. **C** Comparative selection pressure analysis. Selection pressure, measured by *dN*/*dS* ratios, is shown across the five primary SFTSV proteins (RdRp, Gn, Gc, NSs, and NP). **D** Spatial mapping of critical molecular markers on viral proteins. Labeled markers include positively selected sites (blue arrows), genotype B-discriminative sites (purple arrows), and mortality-associated sites (red arrows)

We further analyzed the selection pressure and mutational landscapes across the SFTSV genotypes to identify evolutionary drivers and molecular markers. Several genotype B-specific sites were identified, including RdRp-A1433, NP-R52, Gn-S501, Gn-S218, Gc-S449, and Gc-S100, each with a frequency > 90%, supporting their utility as reliable markers for genotype B (Fig. 7A). After adjusting for age, sex, and hospitalization delay, multivariate logistic regression revealed that RdRp-N828S was independently associated with an increased risk of fatal outcomes (*OR* = 2.52, adjusted *P* < 0.05) and that RdRp-A1433T (genotype B-specific) was significantly associated with a reduced risk of fatal outcomes (*OR* = 0.47, adjusted *P* < 0.05) (Fig. 7B, Fig. 7D, and Fig. S9). BUSTED-PH analysis revealed higher *dN*/*dS* ratios in the NSs and Gn proteins of genotype B than in those of other genotypes (Fig. 7C), suggesting relaxed purifying selection or adaptive evolution in these regions.

## Discussion

In this study, we proposed a unified analytical framework to elucidate the geographically decoupled epidemiology of SFTS across East Asia, a phenomenon we characterized as the “inland high-incidence vs. coastal high-fatality” paradox. By integrating nationwide surveillance, well-characterized clinical cohorts, and large-scale genomic datasets, we demonstrated that this decoupling was likely shaped by dual underlying dynamics: regional incidence patterns were strongly associated with divergent climatic sensitivities, whereas clinical severity was aligned with distinct viral evolutionary trajectories and phylogeographic networks. These findings deepen our understanding of the ecology and evolution of SFTSV and provide an evidence-based foundation for precision surveillance.

To our knowledge, this is the first study to systematically characterize this inland-coastal dichotomy in China through integrated epidemiological, ecological, and genetic profiling. Inland regions, such as Henan province, exhibited higher incidence but comparatively lower CFRs, whereas eastern coastal Zhejiang province showed lower incidence but a significantly higher CFR, a pattern that has been previously noted but not mechanistically explained. In eastern coastal Zhejiang province, the dominance of genotype B associated with a higher CFR, contrasts with the diverse viral community in inland Henan province. In Henan province, genotype C prevails and the CFR is significantly lower. This genotype-specific clinical severity hypothesis is supported by recent animal model data [17], and provides a parsimonious explanation for the observed CFR disparity. Furthermore, the localized association of genotype D with higher mortality in Henan province reinforces this genetic link to clinical severity. Together, these findings illustrate that while genotype-outcome relationships may be context-dependent, they consistently align with regional fatality patterns.

The observed decoupling of SFTS incidence and viral population structures between inland and coastal regions may be attributable to different transmission ecologies. Climate modeling revealed that SFTSV transmission in inland Henan province was predominantly associated with macro-meteorological factors (specifically, temperature), accounting for over 80% of the variation in incidence. This finding underpins a climate-sensitive transmission cycle, in which tick activity is tightly regulated by seasonal conditions. In contrast, the maritime climate of coastal Zhejiang province attenuates these effects, resulting in a prolonged but less predictable transmission season, with models accounting for less than 50% of the incidence variation. Such decoupling implies a multifaceted transmission system in coastal and island settings, where stable microhabitats, diverse host communities, and anthropogenic factors modulate baseline climate-vector relationships. Therefore, region-specific prevention strategies are required. In inland zones, public health alerts and vector controls can be synchronized with seasonal temperature thresholds. Conversely, in coastal regions, where transmission is less predictably linked to climate, interventions should prioritize perennial vector management, personal protection, and monitoring of stable ecological niches that sustain tick populations.

Evolutionary analysis of the predominant coastal genotype B revealed distinct adaptation signals that might underlie its elevated fatality rate. Elevated *dN*/*dS* ratios in NSs (an immune antagonist) and Gn (an entry glycoprotein) suggest relaxed purifying or episodic positive selection, likely reflecting viral adaptation to diverse host environments or immunological pressures. Notably, we identified RdRp-N828S as a mutation significantly associated with clinical mortality. Located within the polymerase finger subdomain, RdRp-N828S may increase virulence by influencing replication fidelity and processivity [51, 52]. Consequently, these genotype B-specific mutations, alongside markers such as RdRp-A1433, might be high-priority targets for the genomic surveillance of highly virulent strains.

Beyond its role as a geographical bottleneck, our findings identify Zhejiang province as a highly active hub for SFTSV evolution and cross-regional transmission. Recombination frequencies in Zhejiang province were significantly elevated compared to those in inland China and the Republic of Korea, with recombinant strains frequently derived from parental sequences originating from the Republic of Korea, Japan, and multiple Chinese provinces. This pattern of cross-regional genetic exchange, particularly within the RdRp (L) and glycoprotein (M) segments, highlights the role of Zhejiang province as a genetic hotspot. Although the direct effect of recombination on virulence requires experimental validation, the concentration of such events in a high-fatality region is epidemiologically significant. Such frequent genetic recombination provides a substrate for adaptive evolution, potentially facilitating the emergence or persistence of variants with increased fitness or pathogenicity.

Bayesian phylogeographic reconstruction positions Zhejiang province of China as a critical intermediary node within the sampled East Asian transmission network. Rather than a linear spatial expansion, the spatiotemporal dynamics are characterized by complex, cross-regional viral exchanges. Internationally, the network is shaped by multidirectional spillovers linking the Republic of Korea, Japan, and eastern China. Domestically, offshore archipelagos and non-coastal centers function as highly active hubs that sustain the regional circulation of SFTSV. Notably, this intricate transmission network coincides precisely with a critical migratory bottleneck along the East Asian-Australasian Flyway. Ecological surveys have documented that this region, together with the Korean Peninsula, funnels approximately two million shorebirds during northward migration alone, with over 90% of the global breeding populations of several species passing through this narrow route [53]. Empirical studies at major stopover sites in the Republic of Korea and remote Japanese islands have directly confirmed that *H. longicornis* frequently parasitizes trans-oceanic avian hosts crossing these waters [54–57]. These observations provide a compelling ecological mechanism for our phylogeographic inferences. Genotype B, dominant in coastal Zhejiang province of China, Japan, and the Republic of Korea but relatively rare in non-coastal regions of China, was likely introduced by cross-oceanic transmission from the Republic of Korea to the Zhejiang province of China and Japan in the late twentieth century [20]. Broad-scale spatial analyses have consistently implicated eastern migratory flyways as the primary conduit for viral dispersal across the sea [16, 22]. Together, our findings corroborate and substantially extend prior hypotheses regarding international viral movement, while uncovering the previously uncharacterized domestic hubs driving regional maintenance [14, 20, 22]. Nevertheless, the precise dynamics of bird–tick–virus dissemination require direct ecological validation, including targeted field studies of tick infestation rates in migratory species along this corridor and longitudinal viral surveillance at stopover sites.

Our analysis of viral diffusion in coastal island environments challenges the traditional stepping-stone model. Instead, our findings corroborate recent phylogeographic evidence indicating that the L2 lineage (corresponding to genotype B in this study) possesses superior transmission velocity and greater spatial reach than the L1 lineage (comprising genotypes A, C, D, and E) [14]. We demonstrated that geographic proximity is a poor predictor of SFTSV genetic relatedness in coastal island environments. Unlike inland regions, where contiguous habitats facilitate gradual dispersal via small terrestrial mammals, the fragmented landscape of the East China Sea archipelago isolates viral transmission, thereby promoting stochastic genetic drift. An example of this isolation is the evolutionary divergence between Xiangshan county and Ningbo urban center of Zhejiang province. Despite their administrative proximity, Xiangshan’s tri-lateral marine boundary and its status as a major deepwater port have fostered a sequestered subclade within genotype B. This configuration exemplifies a “peninsular effect”, whereby geographic constriction and marine isolation act as selective filters, driving evolutionary divergence from the mainland-linked urban population. Such a pattern, characterized by highly localized subclades amidst long-distance genetic links, further supports the hypothesis that SFTSV dispersal is facilitated by anthropogenic activities, including maritime trade routes, rather than purely natural diffusion [58].

Our findings underscore the persistent risk of viral spillovers from diverse animal reservoirs. Although constrained by heterogeneous surveillance data, the host–location network revealed a broader potential host range for SFTSV beyond humans and ticks. The relatively higher documented host diversity in Japan and the Republic of Korea likely reflects more intensive animal surveillance, pointing to a significant gap in China, particularly in key bridging regions such as Zhejiang province of China. A systematic One Health approach integrating surveillance of ticks, livestock, companion animals, and wildlife is urgently needed to identify reservoirs, clarify enzootic maintenance mechanisms, and forecast spillover risks.

Our study has several limitations. First, the observed associations between viral genotypes and clinical outcomes, as well as the identification of the RdRp-N828S mutation, remain observational. Establishing definitive causality and determining whether these genotypes are intrinsically more virulent requires future functional validation using reverse genetics or established animal models. Second, our environmental analysis relied on macro-scale meteorological data. To fully resolve local transmission heterogeneity, future studies must incorporate fine-scale ecological variables such as tick density, land use, vegetation, and host abundance. Third, although our phylogeographic analyses suggest frequent lineage exchanges, the exact mechanisms underlying maritime transmission remain largely speculative. The potential roles of maritime connectivity and specific migratory bird flyways should be viewed as plausible hypotheses that require direct ecological validation. Finally, there was a substantial imbalance in the sample size between the Henan and Zhejiang clinical cohorts. The relatively small number of patients and viruses sequenced from Zhejiang province limited the statistical power of our genotype-specific analyses. Consequently, the increased mortality risk associated with genotype B in Zhejiang province was not statistically significant. Nevertheless, the high virulence of genotype B is well-documented in the Republic of Korea [17], which provides a testable hypothesis for potential genotype-associated clinical severity in China. Expanded longitudinal surveillance and larger, multicenter sequenced cohorts in coastal China are imperative to definitively delineate genotype-linked risks.

## Conclusions

Our study demonstrated that the geographically decoupled epidemiology of SFTS is synergistically driven by divergent ecological settings and distinct viral evolutionary trajectories. Coastal Zhejiang province emerged as a critical evolutionary hub where climate-buffered transmission dynamics and plausible maritime dispersal networks facilitate the sustained circulation of the high-CFR genotype B. In the future, integrating longitudinal genomic surveillance, high-resolution ecological research, and functional virology will be essential to validate these genotype-specific risks and transition from uniform control policies to genotype-oriented precision surveillance across East Asia.

## Supplementary Information

Supplementary Material 1

## Data Availability

All data are available in the main text or supplementary material. The sequence information used in the phylogenetic and phylogeographic analyses are provided in Tables S4 and S7, respectively.

## Abbreviations

BF: Bayes factor
CFR: Case fatality rate
*CI*: Confidence interval
*dN*/*dS*: Non-synonymous/synonymous substitution rate
ESS: Effective sample size
*F*_*ST*_: Fixation index
GAM: Generalized additive model
Gc: Glycoprotein c
Gn: Glycoprotein n
*HPD*: Highest posterior density
IBD: Isolation-by-distance
MCC: Maximum Clade Credibility
MDS: Multidimensional scaling
NP: Nucleocapsid protein
NSs: Non-structural proteins
*OR*: Odds ratio
*PP*: Posterior probability
RdRp: RNA-dependent RNA polymerase
*RR*: Relative risk
RT-PCR: Reverse transcription polymerase chain reaction
SFTS: Severe fever with thrombocytopenia syndrome
SFTSV: Severe fever with thrombocytopenia syndrome virus/*Dabie bandavirus*
tMRCA: The time to the most recent common ancestor
