# Supplementary material for: Phylogeographic epidemiology of Dabie bandavirus in East Asia: divergent transmission networks and genotype‑linked clinical severity
Source: Infect Dis Poverty. 2026 Sep 11;15:102. doi: 10.1186/s40249-026-01500-2 (PMC13563801; doi:10.1186/s40249-026-01500-2)
Supplement: Supplementary file 1 [file 40249_2026_1500_MOESM1_ESM.pdf]

Supplementary Materials

Phylogeographic epidemiology of *Dabie bandavirus* in East Asia: divergent transmission networks and genotype-linked clinical severity

Letian Fang[1,2,3#], Jiluo Liu[1,2,3#], Jimin Sun[4#], Ping Li[1,2,3#], Zhongfa Wang[5#], Hongxia Ni[6], Ling Ye[7], Yiwei Shi[1,2,3], Xin Yang[8], Hanzheng Mu[8], Peng Li[1,2,3], Yibo Ding[1,2,3], Xiaojie Tan[1,2,3], Jianhua Yin[1,2,3], Wei Liu[8*], Guangwen Cao[1,2,3*]

[1] Department of Epidemiology, Second Military Medical University, Shanghai, 200433, China

[2] Key Laboratory of Biological Defense, Ministry of Education, Shanghai, 200433, China.

[3] Shanghai Key Laboratory of Medical Bioprotection, Shanghai, 200433, China.

[4] Zhejiang Provincial Center for Disease Control and Prevention, Hangzhou, 310051, Zhejiang Province, China

[5] Health Monitoring Department, Dinghai Center for Disease Control and Prevention of Zhoushan City, Zhoushan, 316000, Zhejiang Province, China

[6] Ningbo Municipal Center for Disease Control and Prevention, Ningbo, 315010, Zhejiang Province, China.

[7] Department of Infectious Disease Control and Prevention, Daishan Center for Disease Control and Prevention, Zhoushan, 316200, Zhejiang Province, China

[8] State Key Laboratory of Pathogen and Biosecurity, Academy of Military Medical Sciences, Beijing, 100071, China

#These authors contributed equally.

*Correspondence to:

Prof. Guangwen Cao, Department of Epidemiology, Second Military Medical University, 800 Xiangyin Road, Shanghai 200433, China. E-mail: gcao@smmu.edu.cn

Prof. Wei Liu, State Key Laboratory of Pathogen and Biosecurity, Academy of Military Medical Sciences, 20 Dong-Da Street, Fengtai District, Beijing 100071, China. E-mail: lwbime@163.com

Contents

Fig. S1. Temporal distribution of SFTSV genotypes before and after data downsampling. 3

Fig. S2. Temporal signal assessment of SFTSV sequences via root-to-tip regression. 4

Fig. S3. Temporal dynamics of meteorological factors and SFTS incidence in Xinyang and Zhoushan, 2012–2018. 5

Fig. S4. Model diagnostics for generalized additive models of SFTS incidence. 6

Fig. S5. Multidimensional scaling analysis of SFTSV genomic segment. 7

Fig. S6. Temporal distribution SFTSV genotypes by country. 8

Fig. S7. Phylogenetic trees of the three SFTSV genomic segments in Zhejiang province. 9

Fig. S8. Laboratory parameters of patients infected with various SFTSV genotype. 10

Fig. S9. Positively selected amino acid sites detected by multiple methods. 11

Table S1. Primers used in nested PCR for amplifying SFTSV sequences. 12

Table S2. Baseline characteristics of the whole Zhejiang cohort and the subset with sequenced viral genomes. 13

Table S3. Basis dimension (*k*) checking results for generalized additive models. 14

Table S4. Sequences used in the phylogenetic analysis (*n* = 1820). 15

Table S5. Summary of viral reassortment events. 63

Table S6. Putative viral recombination events. 65

Table S7. S-segment sequences used in the phylogeographic analysis (*n* = 207). 68

Table S8. Host range and geographic distribution of SFTSV. 72

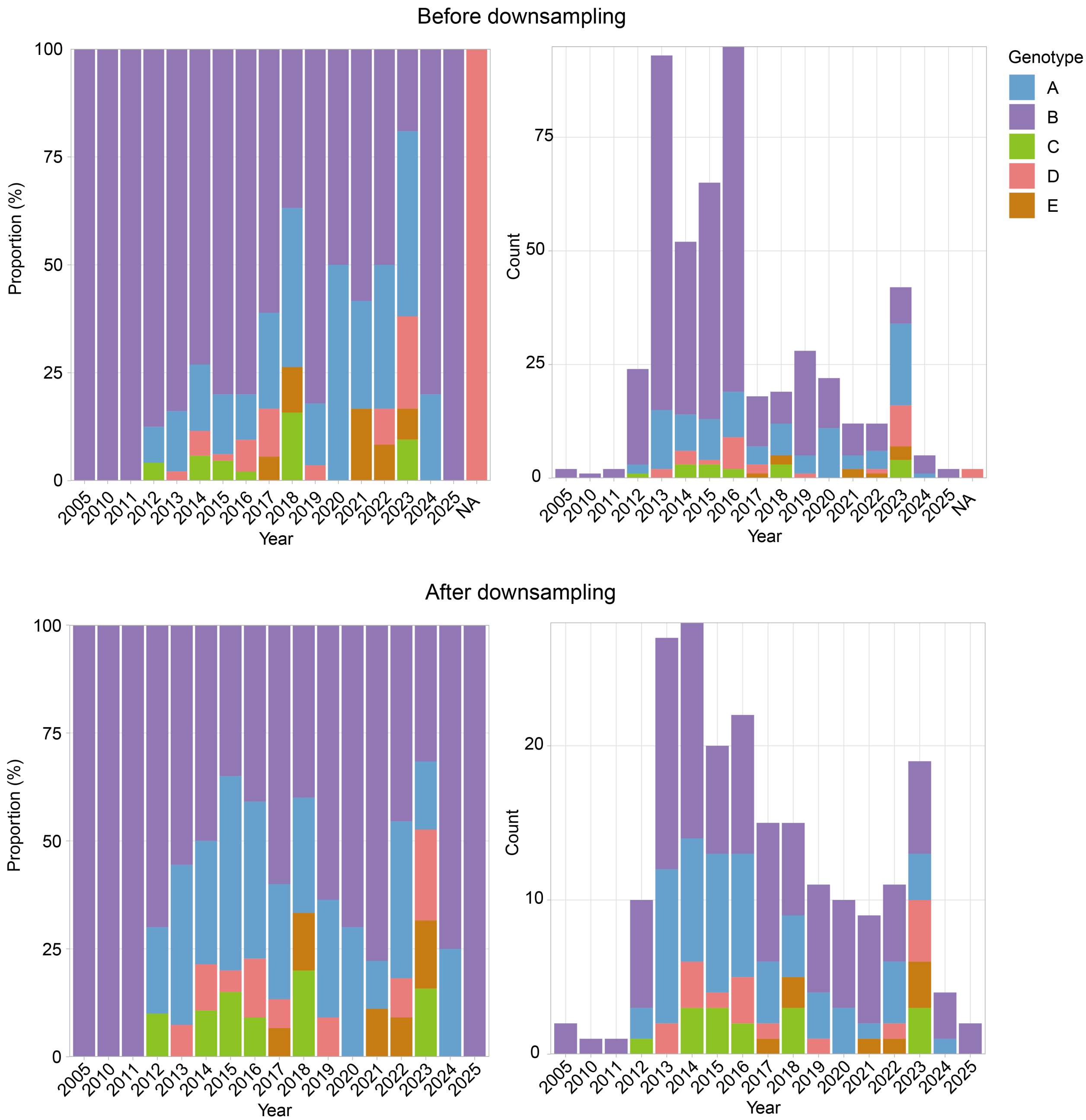

Fig. S1. Temporal distribution of SFTSV genotypes before and after data downsampling. Data from Zhejiang, Republic of Korea, and Japan are shown before (upper panels, $n = 497$) and after (lower panels, $n = 207$) downsampling. Left columns illustrate the relative proportion (%) of each genotype over time, while right columns show the absolute sequence counts. Sequences lacking explicit collection dates are categorized as "NA".

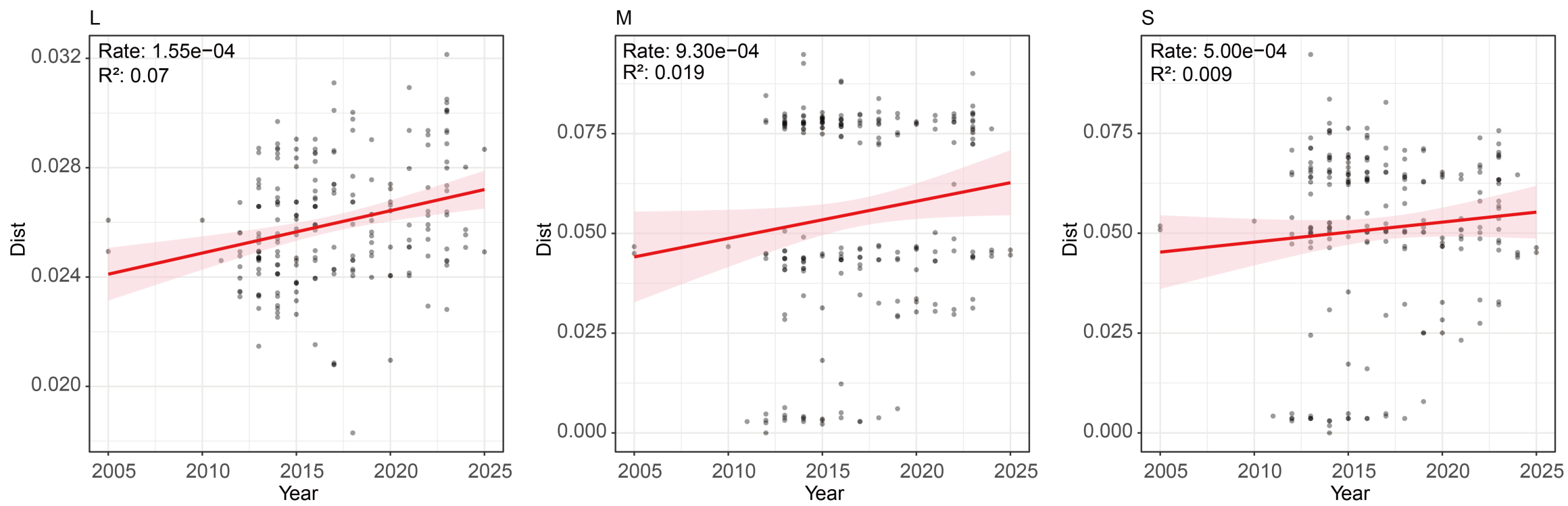

Fig. S2. Temporal signal assessment of SFTSV sequences via root-to-tip regression. Regressions are shown for the L (left), M (middle), and S (right) segments of 207 SFTSV sequences from Zhejiang, Japan, and Republic of Korea. Individual sequences (grey dots) are plotted by their sampling year (x-axis) and root-to-tip genetic distance (y-axis). Linear regression fits (solid red lines) are shown with 95% confidence intervals (pink shaded areas). The estimated substitution rate and regression coefficient of determination (R^2) are annotated in the upper-left corner of each panel.

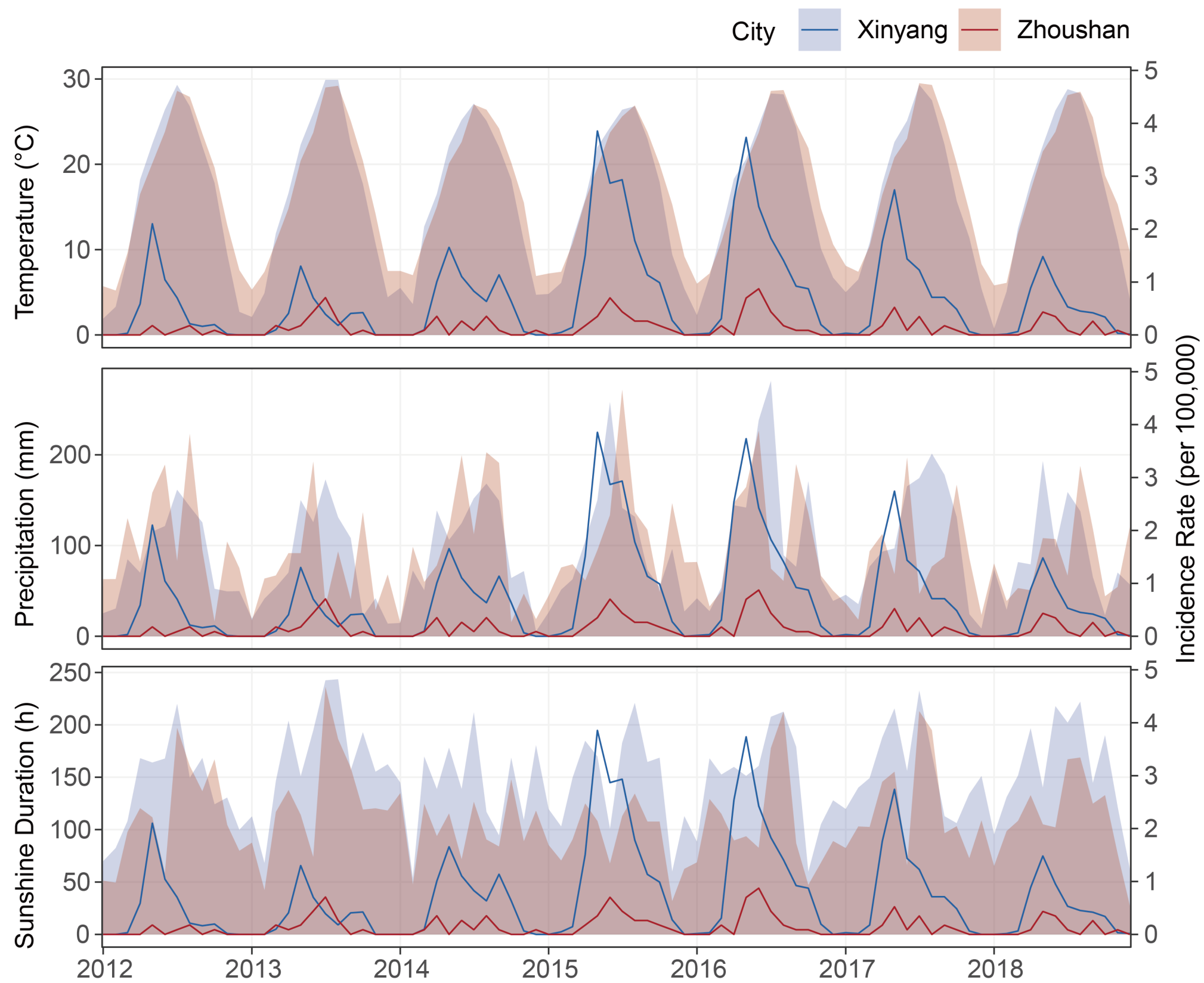

Fig. S3. Temporal dynamics of meteorological factors and SFTS incidence in Xinyang and Zhoushan, 2012–2018.

The panels show monthly data for temperature (°C, top), precipitation (mm, middle), and sunshine duration (h, bottom). Shaded areas indicate the specific meteorological variables (left y-axis), and solid lines illustrate the SFTS incidence rate per 100,000 populations (right y-axis). Blue indicates Xinyang city, and red indicates Zhoushan city.

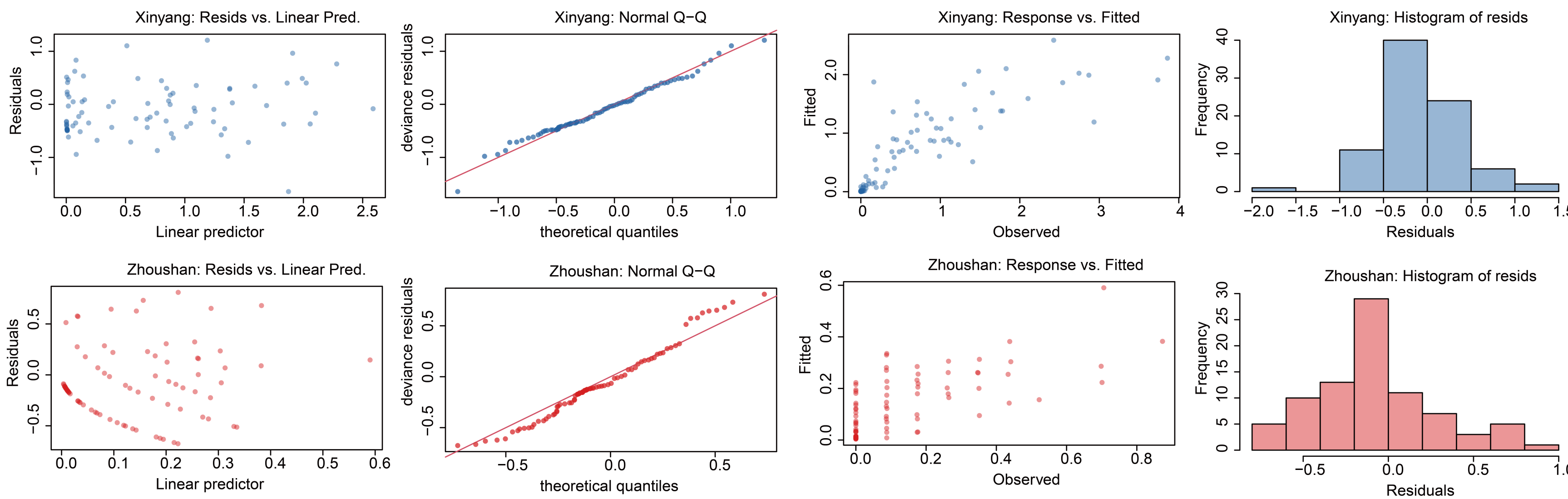

Fig. S4. Model diagnostics for generalized additive models of SFTS incidence.

Diagnostic panels are shown for Xinyang (top row, blue) and Zhoushan (bottom row, red). Columns from left to right display: (1) Residuals versus the linear predictor, verifying the mean-variance relationship; (2) Normal Q-Q plots of deviance residuals against theoretical quantiles, confirming the suitability of the Tweedie distribution; (3) Observed responses versus fitted values, assessing predictive accuracy; and (4) Histograms of residuals, showing approximately symmetric distributions centered around zero. These results collectively validate the model fit, confirming sufficient basis dimensions (P value > 0.05) and the absence of significant overfitting.

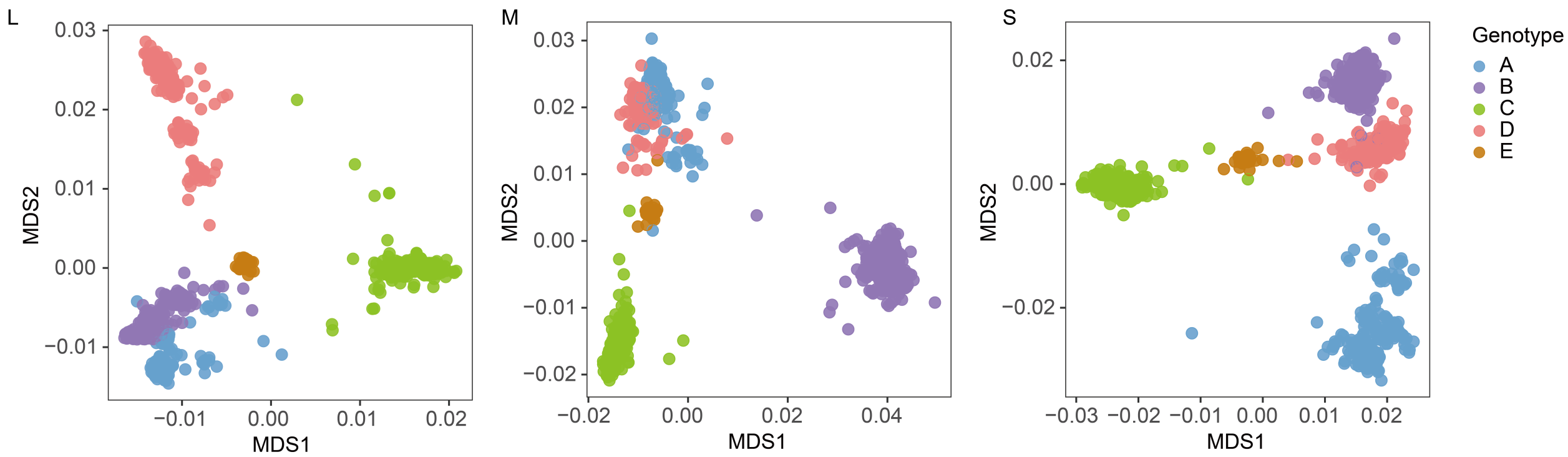

Fig. S5. Multidimensional scaling analysis of SFTSV genomic segment.

The panels display Multidimensional scaling (MDS) plots for the Large (L), Medium (M), and Small (S) segments. Individual sequences are color-coded by their respective genotypes (A–E). The distinct clustering along the first two dimensions (MDS1 and MDS2) demonstrates clear genetic differentiation and well-separated viral populations corresponding to the five genotypes.

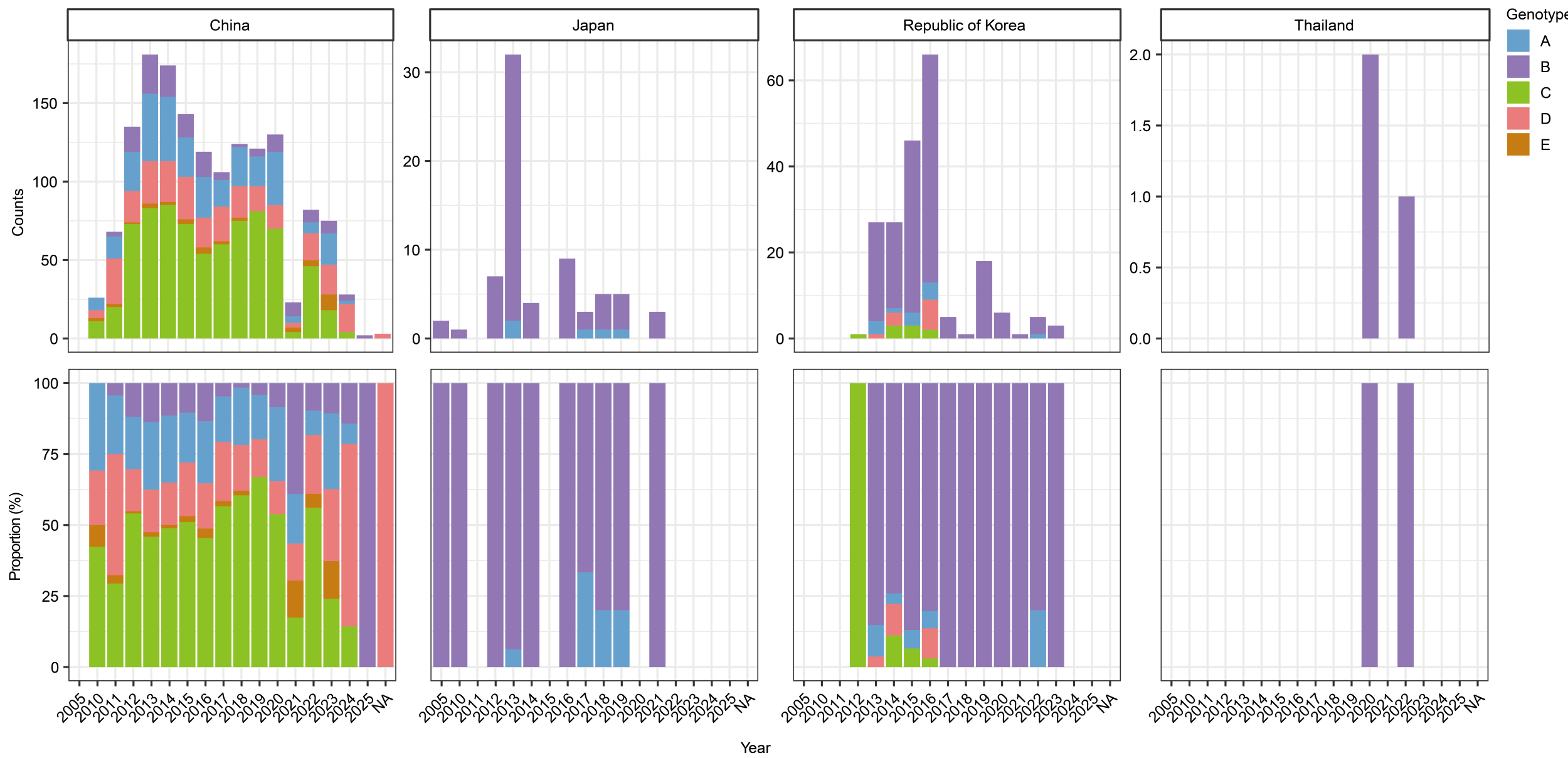

Fig. S6. Temporal distribution SFTSV genotypes by country.

Data are presented for China, Japan, the Republic of Korea, and Thailand (from left to right). The top panels show the absolute yearly counts of SFTSV genotypes, while the bottom panels display their relative proportions (%). Years of sample collection are plotted on the x-axis. The five distinct genotypes (A–E) are indicated by corresponding colors.

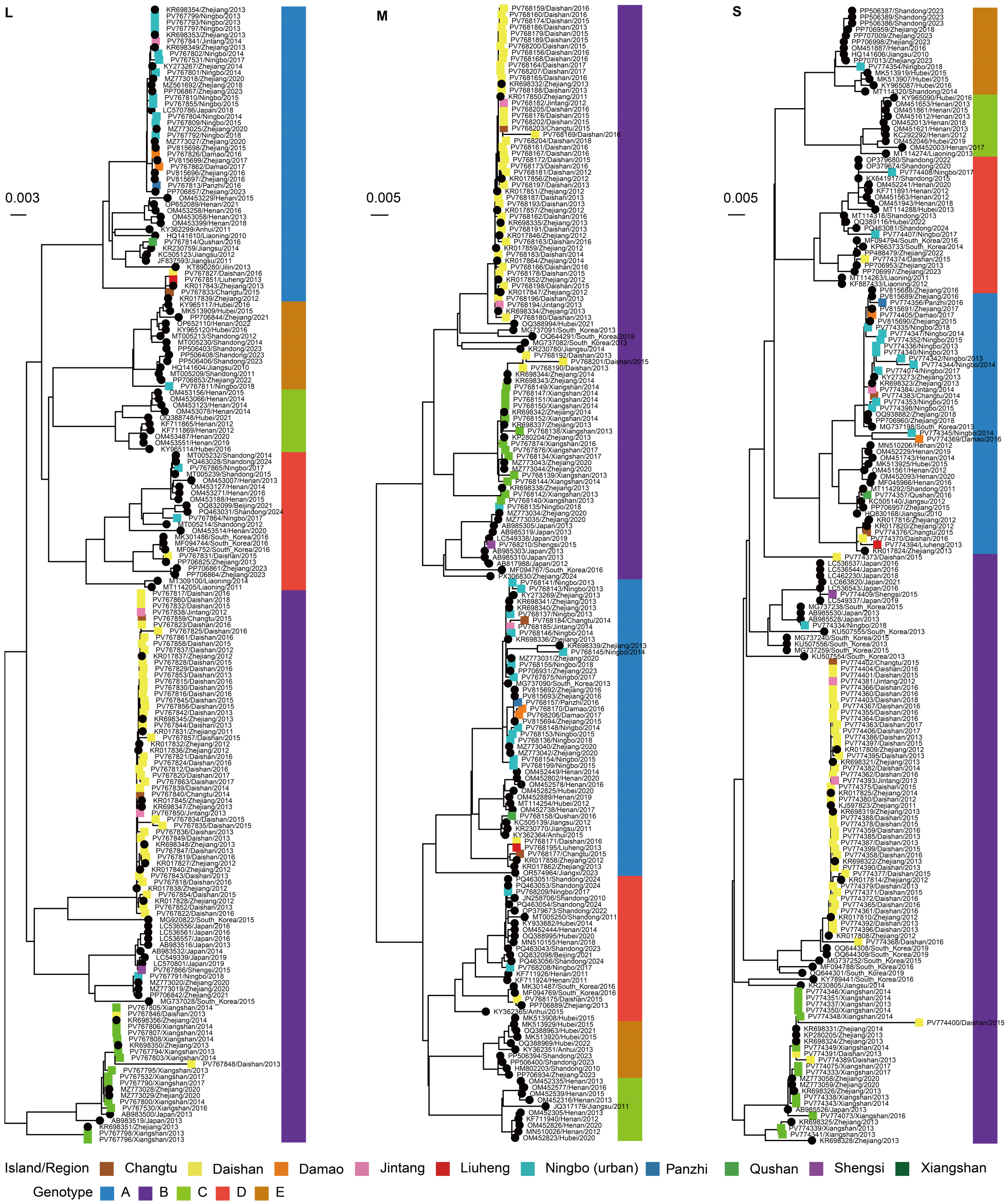

Fig. S7. Phylogenetic trees of the three SFTSV genomic segments in Zhejiang province. Maximum-likelihood phylogenies for the Large (L, left), Medium (M, middle), and Small (S, right) segments. The major genotypes (A–E) are indicated by vertical color bars adjacent to each tree. Colored symbols at the tree tips represent the specific island or region of isolation corresponding to the bottom legend. Scale bars indicate nucleotide substitutions per site.

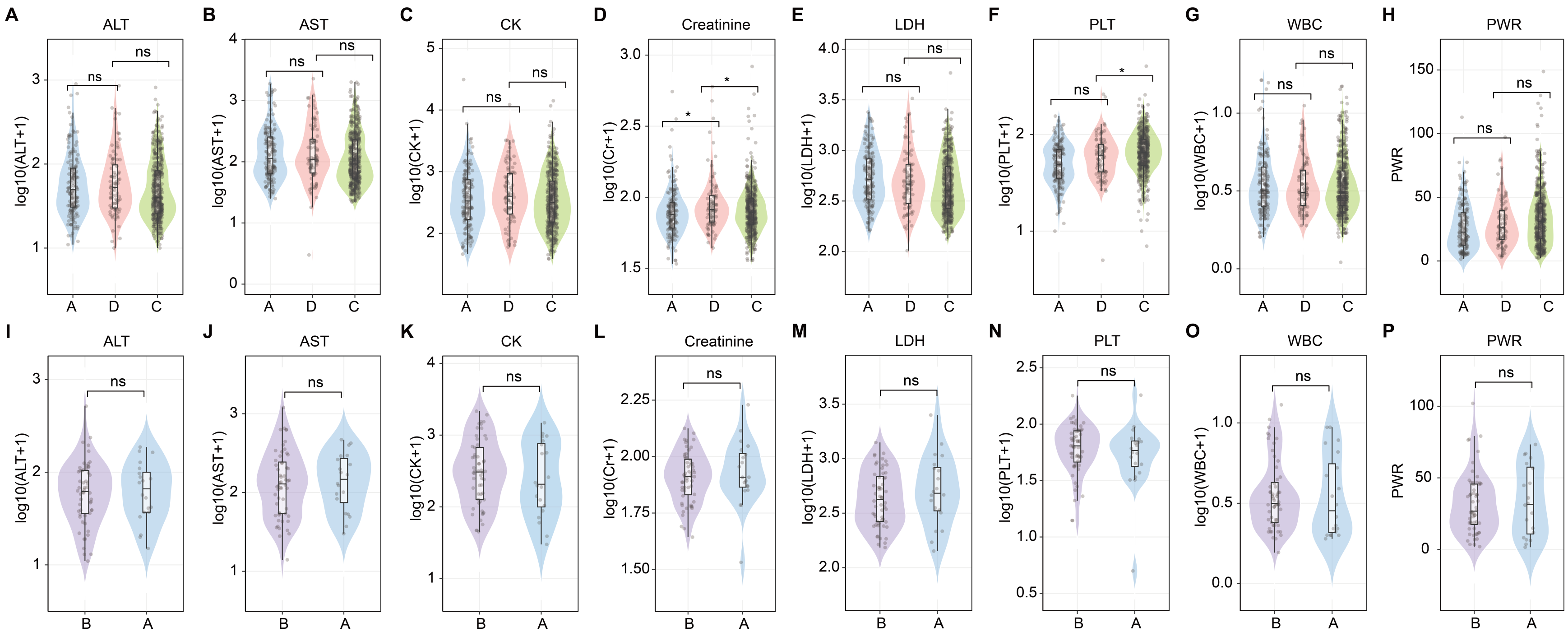

Fig. S8. Laboratory parameters of patients infected with various SFTSV genotype.

(A–H) Distributions of clinical laboratory indicators for patients with genotypes A, D, and C in Henan province. (**I–P**) Corresponding laboratory parameters for patients with genotypes B and A in Zhejiang province. Violin plots are overlaid with box plots (center line, median; box limits, IQR) and individual data points (grey dots). Except for PWR, all laboratory values were transformed via $\log_{10}(x+1)$ to normalize distributions. Differences between genotypes were evaluated using the Kruskal-Wallis test (ns, $P > 0.05$; *, $P < 0.05$). Abbreviations: ALT, alanine aminotransferase; AST, aspartate aminotransferase; CK, creatine kinase; LDH, lactate dehydrogenase; PLT, platelet count; WBC, white blood cell count; PWR, platelet-to-white blood cell ratio.

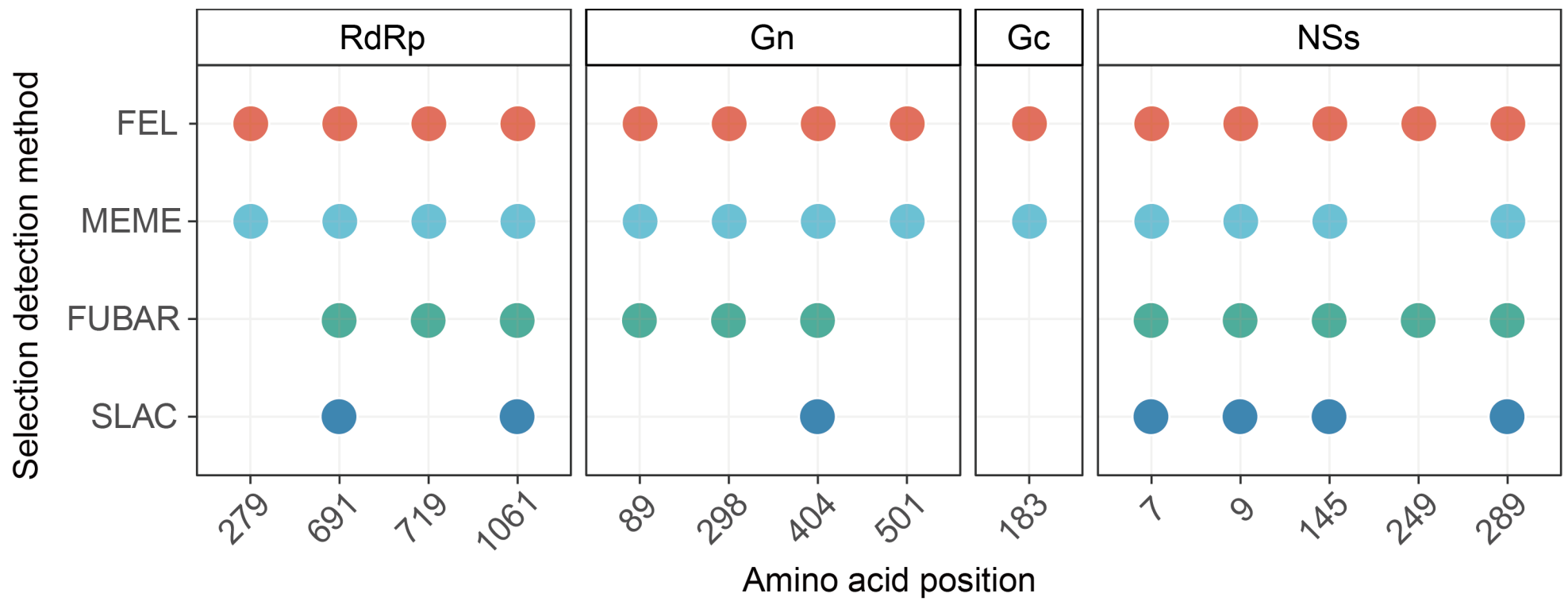

Fig. S9. Positively selected amino acid sites detected by multiple methods.
Panels from left to right display the positively selected sites for the RdRp, Gn, Gc, and NSs proteins of SFTSV. Specific amino acid positions (x-axis) identified as undergoing positive selection are marked with colored dots, categorized by the four detection algorithms used (y-axis: FEL, MEME, FUBAR, and SLAC). A selection event was considered statistically significant if it met the threshold of $P < 0.05$ or a posterior probability > 0.90.

Table S1. Primers used in nested PCR for amplifying SFTSV sequences..

Segment	First round		Second round	
	Primer name	Primer sequence (5'-3')	Primer name	Primer sequence (5'-3')
L	L1F	ACACAAAGACGCCCAGATGA	LN-1F	ACGCCCAGATGAACTTGGAA
	L1R	GACATCTAGAAGGCACTTTGC	LN-1R	AGCCTGAGTCGGTCTTGATGT
	L2F	AGGAGCAACAAGCAAACATCAT	LN-2F	GAATCGAGGGACAGTCAAAC
	L2R	CAATGTACCAGCTGTTCACCA	LN-2R	TTGTGGACCGGCCTGAGATG
	L3F	TGGATTGCATGGTGCGAATTG	LN-3F	GAACATTCCATGCCATCTCAG
	L3R	GATCAGATGACCTAGACTCAG	LN-3R	GTGAGCTAAAAACCTTAGGTC
	L4F	CATGCCAGCYAAATTCCACAG	LN-4F	TTGGGCTGCCATTTCCATGTT
	L4R	TTCTTGTTGGCAGCTCTCCTG	LN-4R	GGACTCCAGGATTCTCATCT
	L5F	GGAACTCTCAGCCACTCTGTT	LN-5F	TGAACAGGATGGGCCTTCCTG
	L5R	GTAGAGAAGGCCTCTATGATC	LN-5R	CTTGTACTCCTCAGTGTATGG
	L6F	TAGCCTAGAAGCTGAGAAGAG	LN-6F	TATCTCCATCCTCAAGCATGT
	L6R	CCTTGGGTCTTCCTATCATTT	LN-6R	AGGAGAACTGAGGCATGTGA
	L7F	ACACTGATATATCAGAGTCAGC	LN-7F	CAATCGAGACCTCTTCTCCT
	L7R	ACACAAAGACCGCCCAGATC	LN-7R	ACCGCCCAGATCTTAAGGAA
M	M1F	ACACAAAGACGGCCAACAATG	MN-1F	ACGGCCAACAATGATGAAAGTC
	M1R	GTCTGGGAATTCACTTTGGC	MN-1R	TCTTCAGCTCCAGAAATGTC
	M2F	GATAGGGTTCTCTGGATAGG	MN-2F	AGGTGATGTTGCTTGTCAGC
	M2R	ATCCCTCCATATGACACAACG	MN-2R	TGCACAAGTGAGCATCTACAC
	M3F	GTRCAAGAGAGCTCATCCAAG	MN-3F	TGTGCTAYAAGGAAGGGACTG
	M3R	TTCTTTGCAGGGTAGCACTG	MN-3R	GGATTTTTTAGAAACTCACGAC
	M4F	GAATTCACATTTGAGGGTAGTT	MN-4F	AGTTGCATGTTCCCAGATGG
	M4R	ACCGGGCATCAGGAACAAAAT	MN-4R	TGAGACATTTTGAGTCYGGAC
	M5F	TGTGATGAGATGGTCCATGCT	MN-5F	AAGGGAGCGGAAATATGAAGG
	M5R	ACCTCCATATCTGAGCCCAA	MN-5R	ACACTCACACCCTTGAAGAC
	M6F	AGAGCTAACAATGCCCTCAG	MN-6F	GAGGACATTCCACCCCATGA
	M6R	AGACTTTGGCGCCTGTCATGC	MN-6R	ACATTTGTCACYTCCCCTGTG
	M7F	CCCCCTGGACATCACAGCTAT	MN-7F	CTGTYAATTATAGAGGCCTTCG
	M7R	CCCATTGCCAAACAAGGCAT	MN-7R	CTTGGGATATTTGCCCCTGT
	M8F	ACTGAACTGTGGGGGACATG	MN-8F	TTGTGGATGGCAGCTACATG
	M8R	ACACAAAGACCGGCCAACACT	MN-8R	AACACTTCAAYRGAACCTCCAT
S	S1F	ACACAAAGAACCCCCAAAAAAGG	SN-1F	AACCCCCAAAAAAGGAAAGACG
	S1R	CCCTTGGCCTTCAGCCACTT	SN-1R	AAGACAGAGTTCACAGCAGC
	S2F	GCAAGATGCCTTCACCAAGA	SN-2F	GAGCCAGCAAGACAGAAGTT
	S2R	ACAGTGTCTTGGATGAGGATG	SN-2R	CCAYATCTGATGGCACACTAT
	S3F	GACAAAATTAGACCTCCTTCG	SN-3F	ACCAATGGCTGGCCAATCTCT
	S3R	TGTACTACAAGGACATGAGG	SN-3R	TCAAGAACAGCTGGGCAATG
	S4F	TATCATGTCCCCTTCAAAAAG	SN-4F	GGCCCCGCCAGTTCTCTCT
	S4R	ACACAAAGAACCCCCTTCATTT	SN-4R	AACCCCCTTCATTTGGAAACCA

Table S2. Baseline characteristics of the whole Zhejiang cohort and the subset with sequenced viral genomes.

Characteristics	Whole cohort (*n* = 131)	Cohort with genomes (*n* = 80)	*P* value
Age, years (median [IQR])	65.00 (55.00, 74.00)	65.00 (55.00, 73.25)	0.632
Male (%)	55 (42.0)	35 (43.8)	0.914
Death (%)	34 (26.6)	25 (31.2)	0.568
Delay from onset to admission, days (median [IQR])	5.00 (4.00, 8.00)	5.00 (4.00, 7.00)	0.414
Fever (%)	127 (96.9)	76 (95.0)	0.729
Chill (%)	39 (29.8)	23 (28.7)	0.998
Headache (%)	45 (34.4)	30 (37.5)	0.752
Fatigue (%)	118 (90.1)	70 (87.5)	0.723
Myalgia (%)	91 (69.5)	51 (63.7)	0.479
Anorexia (%)	62 (47.3)	39 (48.8)	0.953
Nausea (%)	2 (1.5)	1 (1.2)	1.000
Vomiting (%)	77 (58.8)	50 (62.5)	0.696
Abdominal pain (%)	79 (60.3)	42 (52.5)	0.333
Diarrhea (%)	52 (39.7)	29 (36.2)	0.724
Hemorrhage (%)	15 (11.5)	12 (15.0)	0.592
Lymphadenopathy (%)	46 (35.1)	30 (37.5)	0.84
ALT, U/L (median [IQR])	60.00 (33.50, 98.00)	63.00 (35.75, 103.00)	0.524
AST, U/L (median [IQR])	122.00 (59.50, 239.00)	132.50 (53.75, 262.00)	0.533
CK, U/L (median [IQR])	276.00 (121.50, 709.00)	297.50 (122.75, 736.00)	0.885
creatinine, µmol/L (median [IQR])	77.00 (66.50, 96.50)	80.60 (69.60, 98.00)	0.357
LDH, U/L (median [IQR])	476.00 (263.50, 747.50)	455.50 (275.75, 749.50)	0.919
PLT, 10^9/L (median [IQR])	63.00 (43.60, 85.50)	63.25 (43.80, 80.50)	0.863
PWR (median [IQR])	30.00 (17.95, 45.51)	30.17 (14.59, 46.28)	0.986
WBC, 10^9/L (median [IQR])	2.10 (1.40, 3.38)	2.10 (1.30, 3.62)	0.984
Viral load, logged, copies/mL (median [IQR])	6.19 (3.27, 7.29)	5.65 (2.60, 7.17)	0.301

Data are presented as the median (interquartile range [IQR]) for continuous variables, and as the number (percentage) for categorical variables. *P* values were calculated using the Mann-Whitney *U* test for continuous variables, and the Chi-square test or Fisher's exact test for categorical variables. Abbreviations: ALT, alanine aminotransferase; AST, aspartate aminotransferase; CFR, case fatality rate; CK, creatine kinase; LDH, lactate dehydrogenase; PLT, platelet count; PWR, platelet-to-white blood cell ratio; WBC, white blood cell count.

Table S3. Basis dimension (*k*) checking results for generalized additive models.

City	Smooth Term	*k'*	edf	*k*-index	*P* value
Xinyang	s(Temperature)	9.00	4.28	1.21	0.97
	s(Precipitation)	9.00	2.76	0.94	0.41
	s(Sunshine)	9.00	1.78	1.03	0.73
Zhoushan	s(Temperature)	9.00	2.83	0.94	0.41
	s(Precipitation)	9.00	1.00	1.01	0.62
	s(Sunshine)	9.00	3.73	1.14	0.96

The table presents the diagnostic checking results to assess the adequacy of the basis dimensions (*k*) for the non-linear smooth terms in the generalized additive models (GAMs) for Xinyang and Zhoushan. *k'*, maximum possible degrees of freedom for the smooth term; edf, estimated degrees of freedom; *k*-index, a measure of residual randomness. The *P* value evaluates whether the specified basis dimension is sufficiently large. A non-significant *P* value ($P > 0.05$) across all meteorological predictors confirms that the chosen basis dimensions were adequate and that the models effectively captured the non-linear relationships without significant loss of information.

Table S4. Sequences used in the phylogenetic analysis (*n* = 1820).

Isolate	Year	Location	L		M		S	
			Accession	Genotype	Accession	Genotype	Accession	Genotype
HN13/China/2010	2010	Henan	HQ141598	A	HQ141599	A	HQ141600	A
HN6/China/2010	2010	Henan	HQ141595	C	HQ141596	C	HQ141597	C
2011YGS5	2011	Henan	KF711884	D	KF711925	D	KF711893	D
2011YGS7	2011	Henan	KF711885	D	KF711922	D	KF711895	D
2011YPQ11	2011	Henan	KF711886	D	KF711926	D	KF711897	D
2011YPQ12	2011	Henan	KF711880	C	KF711923	D	KF711915	C
2011YPQ17	2011	Henan	KF711887	D	KF711927	D	KF711896	D
12-China_Hebei-24	2012	Hebei	OM452951	C	OM452253	C	OM451571	C
2011YSC22	2011	Henan	KF711888	D	KF711945	C	KF711894	D
2011YSC60	2011	Henan	KF711863	A	KF711924	D	KF711899	A
2011YXX9	2011	Henan	KF711889	D	JQ341189	A	KF711898	D
WCH/97/HN/China/2011	2011	Henan	JQ341188	A	KF356537	D	JQ341190	A
YGS1	2011	Henan	KF356549	D	KF356538	C	KF356525	D
YNY1	2011	Henan	KF356552	C	KF356530	C	KF356526	C
YPQ133	2011	Henan	KF356542	C	KF356531	C	KF356518	C
YPQ2	2011	Henan	KF356543	C	KF356532	C	KF356519	C
YPQ5	2011	Henan	KF356544	C	KF356539	C	KF356520	C
YPQX03	2011	Henan	KF356550	C	KF356536	D	KF356527	C
YSC19	2011	Henan	KF356548	D	KF356535	C	KF356524	D
YSC3	2011	Henan	KF356547	C	KF356529	C	KF356523	A
YSH39	2011	Henan	KF356541	C	KF356533	C	KF356517	C
YXX1	2011	Henan	KF356545	C	KF356534	A	KF356521	C
YXX2	2011	Henan	KF356546	A	KF711930	A	KF356522	A
2012YGS10	2012	Henan	KF711872	C	KF711937	C	KF711909	C
2012YGS4	2012	Henan	KF711875	C	KF711933	C	KF711904	C
2012YSC6	2012	Henan	KF711879	C	KF711931	C	KF711902	C
2012YSH10	2012	Henan	KF711868	C	KF711940	C	KF711911	C
2012YSH104	2012	Henan	KF711867	C	KF711943	C	KF711912	C
2012YSH105	2012	Henan	KF711869	C	KF711944	C	KF711914	C
2012YSH107	2012	Henan	KF711864	C	KF711942	C	KF711916	C
2012YSH14	2012	Henan	KF711876	C	KF711938	C	KF711910	C
2012YSH16	2012	Henan	KF711874	C	KF711934	C	KF711908	C
2012YSH27	2012	Henan	KF711878	C	KF711932	C	KF711903	C
2012YSH37	2012	Henan	KF711871	C	KF711936	C	KF711905	C
2012YSH6	2012	Henan	KF711882	D	KF711921	D	KF711892	D

Isolate	Year	Location	L		M		S	
			Accession	Genotype	Accession	Genotype	Accession	Genotype
2012YSH86	2012	Henan	KF711877	C	KF711939	C	KF711907	C
2012YSH89	2012	Henan	KF711873	C	KF711935	C	KF711906	C
2012YSH9	2012	Henan	KF711881	D	KF711919	D	KF711890	D
2012YSH91	2012	Henan	KF711883	D	KF711920	D	KF711891	D
2012YSH92	2012	Henan	KF711865	C	KF711941	C	KF711917	C
2012YSH93	2012	Henan	KF711866	C	KF711946	C	KF711913	C
2012YXX1	2012	Henan	KF711862	A	KF711929	A	KF711901	A
HNXY_115	2012	Henan	KC292338	C	KC292312	C	KC292285	C
HNXY_130	2012	Henan	KC292342	C	KC292316	C	KC292289	C
12-China_Hubei-21	2012	Hubei	OM452947	A	OM452249	A	OM451566	A
HNXY_144	2012	Henan	KC292333	C	KC292307	C	KC292280	C
HNXY_157	2012	Henan	KC292331	C	KC292305	C	KC292278	C
HNXY_164	2012	Henan	KC292340	C	KC292314	C	KC292287	C
HNXY_170	2012	Henan	KC292353	C	KC292304	C	KC292277	C
HNXY_174	2012	Henan	KC292332	C	KC292306	C	KC292279	C
HNXY_182	2012	Henan	KC292350	C	KC292324	C	KC292297	C
HNXY_186	2012	Henan	KC292334	C	KC292308	C	KC292281	C
HNXY_188	2012	Henan	KC292328	A	KC292301	A	KC292274	A
HNXY_191	2012	Henan	KC292349	A	KC292323	A	KC292296	A
HNXY_195	2012	Henan	KC292352	C	KC292326	C	KC292299	C
HNXY_2	2012	Henan	KC292344	C	KC292318	C	KC292291	C
HNXY_202	2012	Henan	KC292351	C	KC292325	C	KC292298	C
HNXY_206	2012	Henan	KC292329	A	KC292302	A	KC292275	A
HNXY_207	2012	Henan	KC292341	C	KC292315	C	KC292288	C
HNXY_212	2012	Henan	KC292327	D	KC292300	D	KC292273	D
HNXY_224	2012	Henan	KC292343	C	KC292317	C	KC292290	C
HNXY_231	2012	Henan	KC292339	C	KC292313	C	KC292286	C
HNXY_245	2012	Henan	KC292346	C	KC292320	C	KC292293	C
HNXY_262	2012	Henan	KC292335	C	KC292309	C	KC292282	C
HNXY_278	2012	Henan	KC292348	A	KC292322	A	KC292295	A
HNXY_293	2012	Henan	KC292330	C	KC292303	C	KC292276	C
HNXY_31	2012	Henan	KC292336	C	KC292310	C	KC292283	C
HNXY_319	2012	Henan	KC292345	C	KC292319	C	KC292292	C
HNXY_327	2012	Henan	KC292347	D	KC292321	D	KC292294	D
HNXY_93	2012	Henan	KC292337	C	KC292311	C	KC292284	C
HB2014-26	2014	Henan	KY933702	C	KY933689	C	KY933676	C
RAT3	2015	Henan	MF140447	C	MF140448	C	MF140449	C
SFTSV2015YL20X	2015	Henan	MF574211	C	MF574212	C	MF574213	C

Isolate	Year	Location	L		M		S	
			Accession	Genotype	Accession	Genotype	Accession	Genotype
BL136	2016	Henan	MF045953	C	MF045959	C	MF045968	C
BL282	2016	Henan	MF045948	C	MF045956	C	MF045962	C
GP1	2016	Henan	MF045954	A	MF045961	A	MF045967	A
NM1	2016	Henan	MF045950	A	MF045960	A	MF045966	A
SW131	2016	Henan	MF045949	C	MF045957	C	MF045963	C
SW228	2016	Henan	MF045951	C	MF045955	C	MF045964	C
SW249	2016	Henan	MF045952	C	MF045958	C	MF045965	C
2021HNYGS5	2021	Henan	OP652089	A	OP652061	A	OP652033	A
2021HNYSC4	2021	Henan	OP652088	C	OP652060	C	OP652032	C
2022HNYGS1	2022	Henan	OP652085	C	OP652057	C	OP652029	C
2022HNYGS10	2022	Henan	OP652090	C	OP652062	C	OP652034	A
2022HNYGS13	2022	Henan	OP652097	C	OP652069	C	OP652041	C
2022HNYGS2	2022	Henan	OP652086	C	OP652058	C	OP652030	C
2022HNYGS24	2022	Henan	OP652108	C	OP652080	C	OP652052	C
2022HNYGS3	2022	Henan	OP652087	C	OP652059	C	OP652031	C
2022HNYGS8	2022	Henan	OP652095	C	OP652067	C	OP652039	C
2022HNYLS11	2022	Henan	OP652091	D	OP652063	D	OP652035	C
2022HNYLS14	2022	Henan	OP652098	C	OP652070	C	OP652042	C
2022HNYLS15	2022	Henan	OP652099	C	OP652071	C	OP652043	C
2022HNYLS27	2022	Henan	OP652111	C	OP652083	C	OP652055	C
2022HNYLS28	2022	Henan	OP652112	A	OP652084	A	OP652056	A
2022HNYSC22	2022	Henan	OP652106	C	OP652078	C	OP652050	C
2022HNYSC25	2022	Henan	OP652109	C	OP652081	C	OP652053	C
2022HNYSC26	2022	Henan	OP652110	E	OP652082	E	OP652054	E
2022HNYSH12	2022	Henan	OP652092	C	OP652064	C	OP652036	D
2022HNYSH17	2022	Henan	OP652101	C	OP652073	C	OP652045	C
2022HNYSH18	2022	Henan	OP652102	C	OP652074	C	OP652046	C
2022HNYSH19	2022	Henan	OP652103	D	OP652075	D	OP652047	D
2022HNYSH21	2022	Henan	OP652105	C	OP652077	C	OP652049	C
2022HNYSH23	2022	Henan	OP652107	C	OP652079	C	OP652051	C
2022HNYSH6	2022	Henan	OP652093	C	OP652065	C	OP652037	C
2022HNYSH7	2022	Henan	OP652094	A	OP652066	A	OP652038	D
BX-2010/Henan/CHN20	2010	Henan	JF682773	C	JF682774	C	JF682775	C
BX-2010/Henan/CHN69	2010	Henan	JF682776	C	JF682777	C	JF682778	C
BX-2010/Henan/CHNHenan_01	2010	Henan	HQ642766	C	HQ642767	C	HQ642768	C
2010-FQM	2010	Henan	HQ419227	A	HQ419236	A	HQ419240	A
2010-WSQ	2010	Henan	HQ419226	D	HQ419235	D	HQ419239	D
11-China_Henan-1	2011	Henan	MN509835	C	OM452244	C	MN510176	C

Isolate	Year	Location	L		M		S	
			Accession	Genotype	Accession	Genotype	Accession	Genotype
11-China_Henan-13	2011	Henan	MN509837	C	MN510019	C	MN510177	C
11-China_Henan-2	2011	Henan	MN509836	D	OM452245	D	OM451560	D
11-China_Henan-266	2011	Henan	MN509848	A	OM452246	A	MN510181	A
11-China_Henan-273	2011	Henan	MN509849	C	MN510023	C	MN510182	C
11-China_Henan-42	2011	Henan	MN509839	C	MN510024	C	MN510178	C
12-China_Anhui-154	2012	Henan	MN509863	C	MN510025	C	MN510198	C
12-China_Henan-103	2012	Henan	MN509858	C	MN510026	C	MN510194	C
12-China_Henan-108	2012	Henan	OM452943	A	OM452247	A	OM451561	A
12-China_Henan-109	2012	Henan	MN509859	D	MN510027	D	OM451562	D
12-China_Henan-110	2012	Henan	MN509860	C	MN510028	C	MN510195	C
12-China_Henan-114	2012	Henan	MN509861	D	MN510029	D	OM451563	D
12-China_Henan-127	2012	Henan	OM452944	A	OM452248	A	MN510196	A
12-China_Henan-13	2012	Henan	OM452945	C	MN510030	C	OM451564	C
12-China_Henan-143	2012	Henan	MN509862	C	MN510031	C	MN510197	C
12-China_Henan-194	2012	Henan	OM452946	C	MN510034	C	OM451565	C
12-China_Henan-220	2012	Henan	OM452948	C	OM452250	C	OM451567	C
12-China_Henan-224	2012	Henan	OM452949	C	OM452251	C	MN510199	C
12-China_Henan-23	2012	Henan	MN509851	C	MN510035	C	OM451568	C
12-China_Henan-234	2012	Henan	MN509864	C	MN510036	C	OM451569	C
12-China_Henan-237	2012	Henan	OM452950	C	OM452252	C	OM451570	C
12-China_Henan-265	2012	Henan	MN509866	C	MN510037	C	MN510201	C
12-China_Henan-270	2012	Henan	MN509867	C	MN510038	C	MN510202	C
12-China_Henan-271	2012	Henan	MN509868	C	OM452254	C	MN510203	C
12-China_Henan-279	2012	Henan	OM452952	A	OM452255	A	MN510204	A
12-China_Henan-30	2012	Henan	OM452953	A	OM452256	A	OM451572	A
12-China_Henan-32	2012	Henan	OM452954	C	OM452257	C	OM451573	C
12-China_Henan-324	2012	Henan	OM452955	A	OM452258	A	MN510206	A
12-China_Henan-36	2012	Henan	OM452956	C	MN510040	C	OM451574	C
12-China_Henan-37	2012	Henan	OM452957	D	MN510041	D	OM451575	D
12-China_Henan-40	2012	Henan	OM452958	D	OM452259	D	OM451576	D
12-China_Henan-41	2012	Henan	OM452959	C	OM452260	C	OM451577	C
12-China_Henan-48	2012	Henan	MN509852	C	MN510043	C	MN510186	C
12-China_Henan-5	2012	Henan	OM452960	A	OM452261	A	OM451578	A
12-China_Henan-53	2012	Henan	MN509854	C	MN510045	C	MN510188	C
12-China_Henan-54	2012	Henan	MN509855	C	OM452262	C	OM451579	C
12-China_Henan-55	2012	Henan	MN509856	C	MN510046	C	MN510189	C
12-China_Henan-56	2012	Henan	OM452961	A	OM452263	A	OM451580	A
12-China_Henan-64	2012	Henan	OM452962	C	OM452264	C	OM451581	C

Isolate	Year	Location	L		M		S	
			Accession	Genotype	Accession	Genotype	Accession	Genotype
12-China_Henan-65	2012	Henan	OM452963	C	OM452265	C	OM451582	C
12-China_Henan-7	2012	Henan	OM452964	C	OM452266	C	MN510184	C
12-China_Henan-9	2012	Henan	OM452965	C	MN510050	C	OM451583	C
12-China_Henan-90	2012	Henan	OM452966	B	OM452267	B	OM451584	B
12-China_Hubei-242	2012	Henan	MN509865	C	MN510051	C	MN510200	C
13-China_Henan-100	2013	Henan	OM452967	C	MN510053	D	OM451585	D
13-China_Henan-101	2013	Henan	OM452968	C	OM452268	C	OM451586	C
13-China_Henan-110	2013	Henan	MN509876	C	MN510054	C	OM451587	C
13-China_Henan-112	2013	Henan	OM452969	A	OM452269	A	OM451588	A
13-China_Henan-116	2013	Henan	OM452970	C	OM452304	C	OM451624	C
13-China_Henan-118	2013	Henan	OM452971	C	OM452270	C	OM451627	D
13-China_Henan-119	2013	Henan	OM452972	C	OM452271	C	OM451589	C
13-China_Henan-120	2013	Henan	OM452973	C	OM452272	C	OM451590	C
13-China_Henan-122	2013	Henan	OM452974	A	OM452273	C	OM451679	D
13-China_Henan-126	2013	Henan	OM452975	D	OM452274	A	OM451591	C
13-China_Henan-128	2013	Henan	MN509880	C	MN510055	D	OM451592	C
13-China_Henan-129	2013	Henan	OM452976	C	MN510056	C	OM451593	A
13-China_Henan-130	2013	Henan	OM452977	C	OM452275	C	OM451594	D
13-China_Henan-132	2013	Henan	OM452978	C	OM452276	C	OM451595	C
13-China_Henan-139	2013	Henan	OM452979	C	OM452277	C	OM451596	C
13-China_Henan-141	2013	Henan	OM452980	A	OM452278	C	OM451597	C
13-China_Henan-142	2013	Henan	OM452981	C	OM452279	A	OM451598	C
13-China_Henan-144	2013	Henan	OM452982	C	OM452280	C	OM451599	C
13-China_Henan-147	2013	Henan	MN509881	C	OM452281	C	MN510211	A
13-China_Henan-154	2013	Henan	OM452983	A	OM452282	C	OM451600	C
13-China_Henan-155	2013	Henan	OM452984	C	OM452283	A	OM451601	C
13-China_Henan-160	2013	Henan	MN509882	C	OM452284	C	OM451602	C
13-China_Henan-166	2013	Henan	OM452985	C	OM452285	C	MN510212	A
13-China_Henan-170	2013	Henan	OM452986	C	OM452286	C	OM451603	C
13-China_Henan-177	2013	Henan	OM452987	C	OM452287	C	OM451604	C
13-China_Henan-178	2013	Henan	MN509883	C	OM452288	C	MN510214	C
13-China_Henan-181	2013	Henan	OM452988	C	OM452289	C	OM451605	C
13-China_Henan-192	2013	Henan	OM452989	C	OM452290	C	OM451606	C
13-China_Henan-195	2013	Henan	OM452990	A	OM452291	C	OM451607	C
13-China_Henan-198	2013	Henan	OM452991	D	OM452292	A	OM451608	C
13-China_Henan-200	2013	Henan	OM452992	C	MN510058	D	OM451609	C
13-China_Henan-201	2013	Henan	OM452993	C	OM452293	C	OM451610	A
13-China_Henan-212	2013	Henan	MN509885	C	OM452294	C	OM451611	D

Isolate	Year	Location	L		M		S	
			Accession	Genotype	Accession	Genotype	Accession	Genotype
13-China_Henan-213	2013	Henan	OM452994	D	OM452295	C	OM451612	C
13-China_Henan-219	2013	Henan	OM452995	C	OM452296	D	OM451613	C
13-China_Henan-221	2013	Henan	OM452996	C	OM452297	C	OM451614	C
13-China_Henan-224	2013	Henan	OM452997	C	OM452298	C	OM451615	D
13-China_Henan-229	2013	Henan	OM452998	A	OM452299	C	OM451616	C
13-China_Henan-238	2013	Henan	OM452999	A	OM452300	A	OM451617	C
13-China_Henan-24	2013	Henan	OM453000	C	OM452301	A	OM451618	C
13-China_Henan-240	2013	Henan	OM453001	C	MN510060	C	OM451619	A
13-China_Henan-246	2013	Henan	OM453002	A	OM452302	C	OM451620	A
13-China_Henan-254	2013	Henan	OM453003	C	OM452303	A	OM451621	C
13-China_Henan-255	2013	Henan	MN509888	C	OM452305	C	OM451622	C
13-China_Henan-258	2013	Henan	OM453004	C	OM452306	C	OM451623	A
13-China_Henan-262	2013	Henan	OM453005	D	OM452307	C	OM451625	C
13-China_Henan-263	2013	Henan	MN509890	C	OM452308	D	OM451626	C
13-China_Henan-264	2013	Henan	OM453006	C	OM452309	C	OM451628	C
13-China_Henan-266	2013	Henan	OM453007	D	OM452310	C	OM451629	D
13-China_Henan-271	2013	Henan	OM453008	C	OM452311	D	MN510216	C
13-China_Henan-275	2013	Henan	OM453009	A	OM452312	C	MN510217	C
13-China_Henan-279	2013	Henan	OM453010	D	OM452313	A	OM451630	D
13-China_Henan-280	2013	Henan	OM453011	C	OM452314	D	OM451631	C
13-China_Henan-288	2013	Henan	MN509891	C	OM452315	C	MN510218	A
13-China_Henan-29	2013	Henan	OM453012	C	MN510061	C	OM451632	D
13-China_Henan-292	2013	Henan	OM453013	A	OM452316	C	OM451633	C
13-China_Henan-293	2013	Henan	OM453014	A	OM452317	A	MN510219	C
13-China_Henan-294	2013	Henan	OM453015	C	OM452318	A	OM451634	C
13-China_Henan-295	2013	Henan	OM453016	A	OM452319	C	MN510220	A
13-China_Henan-297	2013	Henan	OM453017	D	OM452320	A	OM451635	A
13-China_Henan-299	2013	Henan	OM453018	D	MN510062	D	OM451636	C
13-China_Henan-30	2013	Henan	OM453019	C	OM452321	D	MN510221	A
13-China_Henan-300	2013	Henan	OM453020	A	OM452322	C	OM451637	D
13-China_Henan-302	2013	Henan	MN509892	C	OM452323	A	OM451638	D
13-China_Henan-304	2013	Henan	OM453021	C	OM452324	C	OM451639	C
13-China_Henan-31	2013	Henan	OM453022	A	OM452325	C	OM451640	A
13-China_Henan-310	2013	Henan	OM453023	C	OM452326	A	OM451641	C
13-China_Henan-311	2013	Henan	OM453024	C	OM452327	C	OM451642	C
13-China_Henan-313	2013	Henan	OM453025	A	OM452328	C	OM451643	A
13-China_Henan-320	2013	Henan	OM453026	C	OM452329	A	OM451644	C
13-China_Henan-324	2013	Henan	OM453027	A	OM452330	C	OM451645	C

Isolate	Year	Location	L		M		S	
			Accession	Genotype	Accession	Genotype	Accession	Genotype
13-China_Henan-357	2013	Henan	OM453028	C	OM452331	A	OM451646	A
13-China_Henan-364	2013	Henan	OM453029	D	MN510063	C	OM451647	C
13-China_Henan-368	2013	Henan	OM453030	C	OM452332	D	OM451648	A
13-China_Henan-369	2013	Henan	MN509895	C	OM452333	C	OM451649	C
13-China_Henan-37	2013	Henan	OM453031	C	OM452334	C	OM451650	D
13-China_Henan-371	2013	Henan	OM453032	D	OM452335	C	OM451651	C
13-China_Henan-38	2013	Henan	OM453033	A	OM452336	D	OM451652	C
13-China_Henan-382	2013	Henan	MN509896	D	OM452337	A	OM451653	C
13-China_Henan-39	2013	Henan	MN509871	C	OM452338	D	OM451654	D
13-China_Henan-392	2013	Henan	MN509898	D	OM452339	C	OM451655	A
13-China_Henan-398	2013	Henan	OM453034	A	MN510066	D	OM451656	D
13-China_Henan-40	2013	Henan	MN509872	C	OM452340	A	OM451657	C
13-China_Henan-408	2013	Henan	OM453035	C	OM452341	C	OM451658	D
13-China_Henan-420	2013	Henan	OM453036	C	OM452342	C	MN510223	A
13-China_Henan-423	2013	Henan	OM453037	D	OM452343	C	OM451659	C
13-China_Henan-424	2013	Henan	OM453038	C	MN510067	D	OM451660	C
13-China_Henan-425	2013	Henan	OM453039	C	OM452344	C	OM451661	C
13-China_Henan-428	2013	Henan	OM453040	C	OM452345	C	OM451662	D
13-China_Henan-429	2013	Henan	OM453041	A	OM452346	C	OM451663	C
13-China_Henan-44	2013	Henan	OM453042	C	OM452347	A	MN510224	C
13-China_Henan-45	2013	Henan	OM453043	A	OM452348	C	OM451664	C
13-China_Henan-48	2013	Henan	MN509873	C	OM452349	A	MN510225	A
13-China_Henan-50	2013	Henan	OM453044	C	MN510068	C	OM451665	C
13-China_Henan-52	2013	Henan	OM453045	C	OM452350	C	OM451666	A
13-China_Henan-53	2013	Henan	OM453046	C	OM452351	C	OM451667	C
13-China_Henan-54	2013	Henan	OM453047	A	OM452352	C	OM451668	C
13-China_Henan-55	2013	Henan	OM453048	A	OM452353	A	OM451669	C
13-China_Henan-60	2013	Henan	OM453049	A	OM452354	A	OM451670	C
13-China_Henan-62	2013	Henan	OM453050	C	OM452355	A	OM451671	A
13-China_Henan-65	2013	Henan	OM453051	C	OM452356	C	OM451672	A
13-China_Henan-67	2013	Henan	OM453052	C	OM452357	C	OM451673	A
13-China_Henan-68	2013	Henan	OM453053	C	OM452358	C	OM451674	C
13-China_Henan-77	2013	Henan	OM453054	C	OM452359	C	MN510207	C
13-China_Henan-78	2013	Henan	OM453055	C	OM452360	C	OM451675	C
13-China_Henan-86	2013	Henan	OM453056	A	OM452361	C	OM451676	C
13-China_Henan-87	2013	Henan	OM453057	A	OM452362	A	OM451677	C
13-China_Henan-91	2013	Henan	OM453058	A	OM452363	A	OM451678	C
13-China_Henan-93	2013	Henan	OM453059	C	OM452364	A	OM451680	A

Isolate	Year	Location	L		M		S	
			Accession	Genotype	Accession	Genotype	Accession	Genotype
13-China_Henan-94	2013	Henan	OM453060	A	OM452365	C	OM451681	A
13-China_Hubei-113	2013	Henan	MN509877	C	OM452366	A	OM451682	A
13-China_Hubei-124	2013	Henan	MN509878	C	MN510069	C	OM451683	C
13-China_Hubei-252	2013	Henan	MN509887	C	MN510070	C	MN510208	A
13-China_Hubei-256	2013	Henan	MN509889	D	MN510071	D	MN510209	C
13-China_Hubei-321	2013	Henan	MN509894	C	OM452410	C	MN510210	C
AS11	2019	Republic of Korea	OQ644269	B	OM452420	C	OM451721	C
AS3	2019	Republic of Korea	OQ644270	B	MN510073	C	MN510222	C
AS4	2019	Republic of Korea	OQ644271	B	OM452425	D	OM451735	D
AS7	2019	Republic of Korea	OQ644272	B	OM452432	C	OM451741	C
AS8	2019	Republic of Korea	OQ644273	B	MN510075	D	OQ644299	B
AS9	2019	Republic of Korea	OQ644274	B	OQ644284	B	OQ644300	B
JJ15	2019	Republic of Korea	OQ644275	B	OQ644285	B	OQ644301	B
JJ2	2019	Republic of Korea	OQ644276	B	OQ644286	B	OQ644302	B
JJ3	2019	Republic of Korea	OQ644277	B	OQ644287	B	OQ644303	B
JN13	2019	Republic of Korea	OQ644278	B	OQ644288	B	OQ644304	B
JN13tick	2019	Republic of Korea	OQ644279	B	OQ644289	B	OQ644305	B
JN14	2019	Republic of Korea	OQ644280	B	OQ644290	B	OQ644306	B
JN31	2019	Republic of Korea	OQ644281	B	OQ644291	B	OQ644307	B
JN49	2019	Republic of Korea	OQ644282	B	OQ644292	B	OQ644308	B
Tick2016Jeju	2019	Republic of Korea	OQ644283	B	OQ644293	B	OQ644309	B
HLP23_VE6	2017	Republic of Korea	MN450761	B	OQ644294	B	OQ644310	B
16KS10	2016	Republic of Korea	MF094730	B	OQ644295	B	OQ644311	B
16KS15	2016	Republic of Korea	MF094732	D	OQ644296	B	OQ644312	B
16KS17	2016	Republic of Korea	MF094734	D	OQ644297	B	OQ644313	B
16KS19	2016	Republic of Korea	MF094735	B	OQ644298	B	MN450763	B
13-China_Hubei-79	2013	Henan	MN509874	D	MN450762	B	MF094780	B
14-China_Henan-107	2014	Henan	OM453061	C	MF094755	B	MF094782	D
14-China_Henan-109	2014	Henan	OM453062	A	MF094757	D	MF094784	D
14-China_Henan-11	2014	Henan	OM453063	C	MF094759	D	MF094785	B
14-China_Henan-118	2014	Henan	OM453064	C	MF094760	B	OM451684	C
14-China_Henan-129	2014	Henan	OM453065	A	OM452367	C	OM451685	A
14-China_Henan-13	2014	Henan	OM453066	C	OM452368	A	OM451686	C
14-China_Henan-134	2014	Henan	OM453067	D	OM452369	C	OM451687	C
14-China_Henan-143	2014	Henan	OM453068	C	OM452370	C	OM451688	A
14-China_Henan-148	2014	Henan	OM453069	C	OM452371	A	OM451689	C
14-China_Henan-149	2014	Henan	OM453070	C	OM452372	C	OM451690	D
14-China_Henan-15	2014	Henan	OM453071	A	OM452373	D	OM451691	C

Isolate	Year	Location	L		M		S	
			Accession	Genotype	Accession	Genotype	Accession	Genotype
14-China_Henan-151	2014	Henan	OM453072	C	OM452374	C	OM451692	C
14-China_Henan-152	2014	Henan	OM453073	C	OM452375	C	OM451693	C
14-China_Henan-172	2014	Henan	OM453074	A	OM452376	C	OM451694	A
14-China_Henan-174	2014	Henan	OM453075	A	OM452377	A	MN510227	C
14-China_Henan-175	2014	Henan	OM453076	C	OM452378	C	OM451695	C
14-China_Henan-177	2014	Henan	OM453077	C	OM452379	C	OM451696	A
14-China_Henan-178	2014	Henan	OM453078	C	OM452380	A	OM451697	A
14-China_Henan-189	2014	Henan	OM453079	C	OM452381	A	OM451698	C
14-China_Henan-191	2014	Henan	OM453080	A	OM452382	C	OM451699	C
14-China_Henan-193	2014	Henan	OM453081	C	OM452383	C	OM451700	C
14-China_Henan-20	2014	Henan	OM453082	C	OM452384	C	OM451701	C
14-China_Henan-22	2014	Henan	OM453083	A	OM452385	C	MN510228	A
14-China_Henan-222	2014	Henan	OM453084	C	OM452386	A	OM451702	C
14-China_Henan-236	2014	Henan	OM453085	A	OM452387	C	OM451703	C
14-China_Henan-26	2014	Henan	OM453086	C	OM452388	C	OM451704	A
14-China_Henan-3	2014	Henan	OM453087	C	OM452389	A	MN510229	C
14-China_Henan-307	2014	Henan	OM453088	D	OM452390	C	MN510230	A
14-China_Henan-311	2014	Henan	OM453089	A	OM452391	A	OM451705	C
14-China_Henan-312	2014	Henan	OM453090	D	OM452392	C	OM451706	C
14-China_Henan-314	2014	Henan	MN509905	C	OM452393	C	OM451707	D
14-China_Henan-316	2014	Henan	OM453091	C	OM452394	D	OM451708	A
14-China_Henan-320	2014	Henan	OM453092	A	OM452395	A	OM451709	D
14-China_Henan-326	2014	Henan	OM453093	A	OM452396	D	MN510232	C
14-China_Henan-337	2014	Henan	OM453094	C	OM452397	C	OM451710	C
14-China_Henan-346	2014	Henan	OM453095	A	OM452398	C	OM451711	A
14-China_Henan-35	2014	Henan	OM453096	C	OM452399	A	OM451712	A
14-China_Henan-403	2014	Henan	OM453097	A	OM452400	A	MN510233	C
14-China_Henan-422	2014	Henan	OM453098	C	OM452401	C	OM451713	A
14-China_Henan-424	2014	Henan	MN509909	D	OM452402	A	OM451714	C
14-China_Henan-436	2014	Henan	OM453099	C	OM452403	C	OM451715	A
14-China_Henan-438	2014	Henan	OM453100	C	OM452404	A	OM451716	C
14-China_Henan-440	2014	Henan	OM453101	A	OM452405	C	OM451717	D
14-China_Henan-446	2014	Henan	OM453102	C	OM452406	D	OM451718	C
14-China_Henan-452	2014	Henan	MN509911	C	OM452407	C	OM451719	A
14-China_Henan-453	2014	Henan	OM453103	C	OM452408	A	OM451720	A
14-China_Henan-463	2014	Henan	OM453104	C	OM452409	A	OM451722	C
14-China_Henan-474	2014	Henan	OM453105	A	OM452411	C	OM451723	C
14-China_Henan-484	2014	Henan	OM453106	C	OM452412	C	OM451724	C

Isolate	Year	Location	L		M		S	
			Accession	Genotype	Accession	Genotype	Accession	Genotype
14-China_Henan-485	2014	Henan	MN509912	D	OM452413	C	OM451725	C
14-China_Henan-488	2014	Henan	OM453107	C	OM452414	C	OM451726	A
14-China_Henan-49	2014	Henan	OM453108	C	OM452415	A	OM451727	C
14-China_Henan-494	2014	Henan	OM453109	A	OM452416	C	OM451728	D
14-China_Henan-499	2014	Henan	OM453110	C	OM452417	D	OM451729	C
14-China_Henan-5	2014	Henan	OM453111	C	OM452418	C	OM451730	C
14-China_Henan-501	2014	Henan	MN509914	D	OM452419	C	OM451731	A
14-China_Henan-523	2014	Henan	OM453112	A	OM452421	A	OM451732	C
14-China_Henan-529	2014	Henan	OM453113	C	OM452422	C	OM451733	C
14-China_Henan-54	2014	Henan	OM453114	A	OM452423	C	OM451734	D
14-China_Henan-540	2014	Henan	OM453115	A	OM452424	D	MN510236	A
14-China_Henan-542	2014	Henan	OM453116	D	OM452426	A	OM451736	C
14-China_Henan-546	2014	Henan	OM453117	D	OM452427	C	OM451737	A
14-China_Henan-55	2014	Henan	OM453118	C	OM452428	A	OM451738	A
14-China_Henan-555	2014	Henan	OM453119	A	OM452429	A	OM451739	D
14-China_Henan-559	2014	Henan	OM453120	A	OM452430	D	OM451740	D
14-China_Henan-561	2014	Henan	OM453121	A	OM452431	D	OM451742	C
14-China_Henan-562	2014	Henan	OM453122	D	OM452433	C	OM451743	A
14-China_Henan-563	2014	Henan	MN509917	D	OM452434	A	OM451744	A
14-China_Henan-564	2014	Henan	OM453123	C	OM452435	A	OM451745	A
14-China_Henan-567	2014	Henan	MN509918	D	OM452436	A	OM451746	D
14-China_Henan-568	2014	Henan	OM453124	C	OM452437	D	OM451747	D
14-China_Henan-570	2014	Henan	OM453125	C	OM452438	D	OM451748	C
14-China_Henan-571	2014	Henan	MN509919	C	OM452439	C	OM451749	D
14-China_Henan-573	2014	Henan	OM453126	C	OM452440	D	OM451750	C
14-China_Henan-574	2014	Henan	OM453127	D	OM452441	C	OM451751	C
14-China_Henan-578	2014	Henan	OM453128	C	MN510077	C	OM451752	C
14-China_Henan-579	2014	Henan	OM453129	A	OM452442	C	OM451753	C
14-China_Henan-58	2014	Henan	OM453130	C	OM452443	C	OM451754	D
14-China_Henan-581	2014	Henan	OM453131	C	OM452444	D	OM451755	C
14-China_Henan-582	2014	Henan	OM453132	C	MN510078	C	MN510237	A
14-China_Henan-586	2014	Henan	OM453133	A	OM452445	A	OM451756	C
14-China_Henan-592	2014	Henan	OM453134	C	OM452446	C	OM451757	C
14-China_Henan-593	2014	Henan	OM453135	C	OM452447	C	OM451758	C
14-China_Henan-600	2014	Henan	MN509920	D	OM452448	C	OM451759	A
14-China_Henan-601	2014	Henan	OM453136	C	OM452449	A	OM451760	C
14-China_Henan-604	2014	Henan	OM453137	C	OM452450	C	OM451761	C
14-China_Henan-605	2014	Henan	MN509921	C	OM452451	C	OM451762	D

Isolate	Year	Location	L		M		S	
			Accession	Genotype	Accession	Genotype	Accession	Genotype
14-China_Henan-606	2014	Henan	OM453138	C	OM452452	D	OM451763	C
14-China_Henan-607	2014	Henan	OM453139	C	OM452453	C	OM451764	C
14-China_Henan-61	2014	Henan	OM453140	C	OM452454	C	OM451765	C
14-China_Henan-610	2014	Henan	OM453141	C	OM452455	C	OM451766	C
14-China_Henan-613	2014	Henan	MN509922	C	OM452456	C	MN510238	C
14-China_Henan-617	2014	Henan	MN509923	C	OM452457	C	OM451767	C
14-China_Henan-62	2014	Henan	OM453142	A	OM452458	C	OM451768	C
16KS20	2016	Republic of Korea	MF094736	B	OM452459	C	MN510239	C
16KS23	2016	Republic of Korea	MF094737	B	OM452460	C	OM451769	C
16KS24	2016	Republic of Korea	MF094738	B	OM452461	C	OM451823	D
16KS26	2016	Republic of Korea	MF094739	B	OM452462	A	OM451770	A
16KS28	2016	Republic of Korea	MF094740	B	OM452463	C	MF094786	B
16KS29	2016	Republic of Korea	MF094741	B	MF094761	B	MF094787	B
16KS30	2016	Republic of Korea	MF094742	B	MF094762	B	MF094788	B
16KS31	2016	Republic of Korea	MF094743	B	MF094763	B	MF094789	B
16KS33	2016	Republic of Korea	MF094744	D	MF094764	B	MF094790	B
16KS4	2016	Republic of Korea	MF094728	B	MF094765	B	MF094791	B
16KS40	2016	Republic of Korea	MF094745	B	MF094766	B	MF094792	B
16KS41	2016	Republic of Korea	MF094746	B	MF094767	B	MF094793	B
16KS42	2016	Republic of Korea	MF094747	B	MF094768	B	MF094794	D
16KS43	2016	Republic of Korea	MF094748	B	MF094769	D	MF094778	B
16KS45	2016	Republic of Korea	MF094749	B	MF094753	B	MF094795	B
16KS47	2016	Republic of Korea	MF094750	B	MF094770	B	MF094796	B
16KS51	2016	Republic of Korea	MF094751	D	MF094771	B	MF094797	B
16KS52	2016	Republic of Korea	MF094752	D	MF094772	B	MF094798	B
16KS7	2016	Republic of Korea	MF094729	B	MF094773	B	MF094799	A
16MS104	2016	Republic of Korea	MF094733	B	MF094774	A	MF094800	B
16MS62	2016	Republic of Korea	MF094731	B	MF094775	B	MF094801	D
Dog22	2016	Republic of Korea	MH464252	B	MF094776	D	MF094802	D
2015-JJ01	2015	Republic of Korea	MN329150	B	MF094777	D	MF094779	B
Asan	2015	Republic of Korea	MF094804	B	MF094754	B	MF094783	B
KH1	2015	Republic of Korea	MH491549	B	MF094758	B	MF094781	B
KS11	2015	Republic of Korea	MF094807	B	MF094756	B	KY968712	B
KS13	2015	Republic of Korea	MF094805	B	MH464251	B	MN329148	B
KS17	2015	Republic of Korea	MF094808	B	MN329149	B	MF094816	B
KS7	2015	Republic of Korea	MF094803	B	MF094810	B	MH491547	B
MS39	2015	Republic of Korea	MF094806	B	MH491548	B	MF094819	B
KADGH	2013	Republic of Korea	KU507543	B	MF094813	B	MF094817	B

Isolate	Year	Location	L		M		S	
			Accession	Genotype	Accession	Genotype	Accession	Genotype
KADGH4	2013	Republic of Korea	KU507544	B	MF094811	B	MF094820	B
KAGNH	2013	Republic of Korea	KU507545	B	MF094814	B	MF094815	B
KAGNH4	2013	Republic of Korea	KU507546	B	MF094809	B	MF094818	B
KAGWT	2013	Republic of Korea	KY273136	B	MF094812	B	KU507553	B
KAJNH2	2013	Republic of Korea	KU507547	B	KU507548	B	KU507554	B
14-China_Henan-63	2014	Henan	OM453143	C	KU507549	B	KU507555	B
14-China_Henan-66	2014	Henan	OM453144	C	KU507550	B	KU507556	B
14-China_Henan-68	2014	Henan	OM453145	C	KU507551	B	KY273138	B
14-China_Henan-69	2014	Henan	OM453146	C	KY273137	B	KU507557	B
14-China_Henan-70	2014	Henan	OM453147	C	KU507552	B	OM451771	C
14-China_Henan-71	2014	Henan	MN509899	A	OM452464	C	OM451772	C
14-China_Henan-74	2014	Henan	OM453148	C	OM452465	C	OM451773	C
14-China_Henan-76	2014	Henan	OM453149	C	OM452466	C	OM451774	C
14-China_Henan-79	2014	Henan	OM453150	C	OM452467	C	OM451775	C
14-China_Henan-84	2014	Henan	OM453151	A	OM452468	A	OM451776	A
14-China_Henan-86	2014	Henan	OM453152	C	OM452469	C	OM451777	C
14-China_Henan-9	2014	Henan	OM453153	C	OM452470	C	OM451778	C
14-China_Hubei-444	2014	Henan	MN509910	C	OM452471	C	MN510226	C
14-China_Hubei-492	2014	Henan	MN509913	C	OM452472	A	OM451779	A
14-China_Hubei-512	2014	Henan	MN509915	D	OM452473	C	OM451780	C
14-China_Hubei-548	2014	Henan	MN509916	C	OM452474	C	OM451781	C
15-China_Henan-113	2015	Henan	OM453155	C	OM452476	C	MN510235	C
15-China_Henan-117	2015	Henan	OM453156	C	OM452477	C	OM451783	C
15-China_Henan-119	2015	Henan	MN509926	C	MN510079	C	OM451784	C
15-China_Henan-123	2015	Henan	OM453157	C	OM452478	C	MN510242	C
15-China_Henan-127	2015	Henan	OM453158	C	OM452479	C	OM451785	C
15-China_Henan-130	2015	Henan	MN509927	D	OM452480	D	OM451786	C
15-China_Henan-132	2015	Henan	OM453159	C	OM452481	C	OM451787	D
15-China_Henan-134	2015	Henan	OM453160	C	OM452482	C	OM451788	C
15-China_Henan-141	2015	Henan	MN509929	A	OM452484	A	OM451789	C
15-China_Henan-147	2015	Henan	OM453162	A	OM452485	A	OM451791	A
15-China_Henan-15	2015	Henan	OM453163	D	OM452486	C	OM451792	A
15-China_Henan-150	2015	Henan	OM453164	C	OM452487	C	OM451793	C
15-China_Henan-151	2015	Henan	OM453165	C	OM452488	C	MN510243	C
15-China_Henan-153	2015	Henan	OM453166	C	OM452489	C	OM451794	C
15-China_Henan-155	2015	Henan	OM453167	A	OM452490	A	OM451795	C
15-China_Henan-16	2015	Henan	OM453168	C	OM452491	D	OM451796	A
15-China_Henan-160	2015	Henan	OM453169	C	OM452492	C	OM451797	D

Isolate	Year	Location	L		M		S	
			Accession	Genotype	Accession	Genotype	Accession	Genotype
15-China_Henan-165	2015	Henan	OM453170	A	OM452493	A	OM451798	C
15-China_Henan-168	2015	Henan	OM453171	A	OM452494	A	OM451799	A
15-China_Henan-170	2015	Henan	OM453172	C	OM452495	C	OM451800	A
15-China_Henan-174	2015	Henan	OM453173	C	OM452496	C	OM451801	C
15-China_Henan-176	2015	Henan	OM453174	C	OM452497	C	OM451802	C
15-China_Henan-178	2015	Henan	OM453175	C	OM452498	C	OM451803	C
15-China_Henan-179	2015	Henan	OM453176	A	OM452499	A	OM451804	C
15-China_Henan-2	2015	Henan	OM453177	C	OM452500	C	OM451805	A
15-China_Henan-200	2015	Henan	OM453178	C	OM452501	C	OM451806	C
15-China_Henan-201	2015	Henan	MN509931	C	MN510081	C	OM451807	C
15-China_Henan-204	2015	Henan	OM453179	D	MN510082	D	OM451808	C
15-China_Henan-206	2015	Henan	OM453180	A	OM452502	A	OM451809	D
15-China_Henan-211	2015	Henan	MN509932	C	OM452503	C	OM451810	A
15-China_Henan-213	2015	Henan	OM453181	D	OM452504	D	OM451811	C
15-China_Henan-217	2015	Henan	MN509933	D	MN510083	D	OM451812	D
15-China_Henan-221	2015	Henan	MN509934	C	OM452505	C	OM451813	D
15-China_Henan-224	2015	Henan	OM453182	D	OM452506	D	OM451814	C
15-China_Henan-225	2015	Henan	OM453183	C	OM452507	C	OM451815	D
15-China_Henan-226	2015	Henan	OM453184	A	MN510084	A	OM451816	C
15-China_Henan-227	2015	Henan	OM453185	C	OM452508	C	OM451817	A
15-China_Henan-228	2015	Henan	OM453186	A	OM452509	A	OM451818	C
15-China_Henan-229	2015	Henan	OM453187	C	OM452510	C	OM451819	A
15-China_Henan-23	2015	Henan	OM453188	D	MN510085	D	OM451820	C
15-China_Henan-232	2015	Henan	OM453189	C	OM452511	C	OM451821	D
15-China_Henan-238	2015	Henan	OM453190	C	OM452512	C	OM451822	C
15-China_Henan-249	2015	Henan	OM453191	C	OM452513	C	OM451824	C
15-China_Henan-250	2015	Henan	OM453192	C	OM452514	C	OM451825	C
15-China_Henan-256	2015	Henan	MN509937	C	MN510086	C	OM451826	C
15-China_Henan-257	2015	Henan	OM453193	C	OM452515	C	MN510245	C
15-China_Henan-258	2015	Henan	OM453194	C	OM452516	C	OM451827	C
15-China_Henan-260	2015	Henan	MN509939	D	MN510087	D	OM451828	C
15-China_Henan-266	2015	Henan	OM453195	C	OM452517	C	OM451829	D
16-China_Hubei-471	2016	Hubei	OM453261	C	OM452585	C	OM451895	C
CB14	2023	Republic of Korea	PP656910	B	PP656911	B	PP656912	B
CB15	2023	Republic of Korea	PP656913	B	PP656914	B	PP656915	B
CB17	2023	Republic of Korea	PP656919	B	PP656920	B	PP656921	B
GGCB02/2022	2022	Republic of Korea	OR805360	B	OR805359	B	OR805358	B
JJCB01/2022	2022	Republic of Korea	OR797605	B	OR797606	B	OR797607	B

Isolate	Year	Location	L		M		S	
			Accession	Genotype	Accession	Genotype	Accession	Genotype
N39	2022	Republic of Korea	OR113701	A	OR113703	A	OR113705	A
N50	2022	Republic of Korea	OR113702	B	OR113704	B	OR113706	B
SMCB03/2022	2022	Republic of Korea	OR805363	B	OR805362	B	OR805361	B
JJCB01/2021	2021	Republic of Korea	OR797610	B	OR797609	B	OR797608	B
CB16	2020	Republic of Korea	PP656916	B	PP656917	B	PP656918	B
Hl20-8	2020	Republic of Korea	OM160960	B	OM160957	B	OM160981	B
IP417	2020	Republic of Korea	OL773689	B	OL773688	B	OK423755	B
IP418	2020	Republic of Korea	OM179923	B	OM179921	B	OK423754	B
JJCB01	2020	Republic of Korea	MZ501598	B	MZ352108	B	MZ342903	B
IP551	2020	Republic of Korea	MZ363633	B	MZ501599	B	MZ501600	B
Hl19-31-13	2019	Republic of Korea	OM160953	B	OM160983	B	OM160962	B
Hl19-31-4	2019	Republic of Korea	OM160952	B	OM160955	B	OM160979	B
IP46	2019	Republic of Korea	OM179922	B	OM179920	B	OL773687	B
CBCB01	2018	Republic of Korea	MZ501595	B	MZ501596	B	MZ501597	B
AFMC_17-1	2017	Republic of Korea	MT683683	B	MT683684	B	MT683685	B
CB7	2017	Republic of Korea	MK301483	B	MK301484	B	MK301485	B
CUK-JJ01	2017	Republic of Korea	MH937374	B	MH937373	B	MH937372	B
17WD333	2017	Republic of Korea	MK413197	B	MK413198	B	MK413199	B
16KS100	2016	Republic of Korea	MG737067	B	MG737176	B	MG737284	B
16KS104	2016	Republic of Korea	MG737074	B	MG737183	B	MG737291	B
16KS108	2016	Republic of Korea	MG737075	B	MG737184	B	MG737292	B
16KS112	2016	Republic of Korea	MG737076	B	MG737185	B	MG737293	B
16KS115	2016	Republic of Korea	MG737077	A	MG737186	A	MG737294	A
16KS116	2016	Republic of Korea	MG737078	B	MG737187	B	MG737295	B
16KS126	2016	Republic of Korea	MG737079	B	MG737188	B	MG737296	B
16KS128	2016	Republic of Korea	MG737080	B	MG737189	B	MG737297	B
16KS129	2016	Republic of Korea	MG737081	B	MG737190	B	MG737298	B
16KS54	2016	Republic of Korea	MG737045	B	MG737154	B	MG737262	B
16KS55	2016	Republic of Korea	MG737046	D	MG737155	B	MG737263	D
16KS62	2016	Republic of Korea	MG737048	B	MG737157	B	MG737265	B
16KS63	2016	Republic of Korea	MG737049	B	MG737158	B	MG737266	B
16KS71	2016	Republic of Korea	MG737051	B	MG737160	B	MG737268	B
16KS77	2016	Republic of Korea	MG737053	B	MG737162	B	MG737270	B
16KS80	2016	Republic of Korea	MG737054	B	MG737163	B	MG737271	B
16KS83	2016	Republic of Korea	MG737055	B	MG737164	B	MG737272	B
16KS85	2016	Republic of Korea	MG737056	B	MG737165	B	MG737273	B
16KS86	2016	Republic of Korea	MG737057	B	MG737166	B	MG737274	B
16KS89	2016	Republic of Korea	MG737058	C	MG737167	C	MG737275	C

Isolate	Year	Location	L		M		S	
			Accession	Genotype	Accession	Genotype	Accession	Genotype
16KS92	2016	Republic of Korea	MG737059	B	MG737168	B	MG737276	B
16KS96	2016	Republic of Korea	MG737066	A	MG737175	A	MG737283	A
16MS239	2016	Republic of Korea	MG737047	B	MG737156	B	MG737264	B
16MS269	2016	Republic of Korea	MG737050	B	MG737159	B	MG737267	B
16MS286	2016	Republic of Korea	MG737052	B	MG737161	B	MG737269	B
16MS299	2016	Republic of Korea	MG737060	B	MG737169	B	MG737277	B
16MS300	2016	Republic of Korea	MG737061	C	MG737170	C	MG737278	C
16MS310	2016	Republic of Korea	MG737062	B	MG737171	B	MG737279	B
16MS313	2016	Republic of Korea	MG737063	B	MG737172	B	MG737280	B
16MS315	2016	Republic of Korea	MG737064	B	MG737173	B	MG737281	B
16MS323	2016	Republic of Korea	MG737068	B	MG737177	B	MG737285	B
16MS344	2016	Republic of Korea	MG737069	B	MG737178	B	MG737286	B
16MS347	2016	Republic of Korea	MG737070	B	MG737179	B	MG737287	B
16MS351	2016	Republic of Korea	MG737071	B	MG737180	B	MG737288	B
16MS368	2016	Republic of Korea	MG737072	B	MG737181	B	MG737289	B
16MS373	2016	Republic of Korea	MG737073	B	MG737182	B	MG737290	B
CB13	2016	Republic of Korea	MK301489	A	MK301490	A	MK301491	A
CB3	2016	Republic of Korea	KY789435	B	KY789438	B	KY789441	B
15-China_Henan-29	2015	Henan	OM453196	D	OM452518	D	OM451830	C
15-China_Henan-30	2015	Henan	OM453197	C	OM452519	C	OM451831	D
15-China_Henan-316	2015	Henan	MN509941	C	OM452520	C	OM451832	C
15-China_Henan-321	2015	Henan	OM453198	A	OM452521	A	OM451833	C
15-China_Henan-322	2015	Henan	MN509942	C	MN510088	C	MN510247	A
15-China_Henan-323	2015	Henan	OM453199	C	OM452522	C	OM451834	C
15-China_Henan-326	2015	Henan	OM453200	C	OM452523	C	OM451835	C
15-China_Henan-338	2015	Henan	OM453201	C	MN510090	C	OM451836	C
15-China_Henan-34	2015	Henan	MN509924	C	OM452524	C	OM451837	C
15-China_Henan-343	2015	Henan	OM453202	C	OM452525	C	MN510240	C
15-China_Henan-35	2015	Henan	OM453203	C	OM452526	C	OM451838	C
15-China_Henan-351	2015	Henan	OM453204	A	OM452527	A	MN510241	C
15-China_Henan-353	2015	Henan	OM453205	D	OM452528	D	MN510249	A
15-China_Henan-356	2015	Henan	OM453206	D	MN510091	C	OM451839	D
15-China_Henan-363	2015	Henan	MN509945	C	OM452529	C	MN510250	C
15-China_Henan-365	2015	Henan	OM453208	C	OM452531	C	OM451840	C
15-China_Henan-374	2015	Henan	OM453209	A	OM452532	A	OM451842	C
15-China_Henan-380	2015	Henan	MN509947	C	MN510092	C	OM451843	A
15-China_Henan-391	2015	Henan	OM453210	C	OM452533	C	MN510251	C
15-China_Henan-393	2015	Henan	OM453211	C	MN510094	C	OM451844	C

Isolate	Year	Location	L		M		S	
			Accession	Genotype	Accession	Genotype	Accession	Genotype
15-China_Henan-398	2015	Henan	OM453212	D	MN510095	D	OM451845	C
15-China_Henan-4	2015	Henan	OM453213	C	OM452534	C	OM451846	D
15-China_Henan-40	2015	Henan	OM453214	C	OM452535	C	OM451847	C
15-China_Henan-411	2015	Henan	OM453215	A	OM452536	A	OM451848	C
15-China_Henan-413	2015	Henan	OM453216	C	OM452537	C	MN510252	A
15-China_Henan-414	2015	Henan	MN509952	C	OM452538	C	OM451849	C
15-China_Henan-415	2015	Henan	MN509953	C	OM452539	C	OM451850	C
15-China_Henan-430	2015	Henan	MN509954	C	MN510099	C	OM451851	C
15-China_Henan-55	2015	Henan	MN509925	D	MN510107	D	MN510253	C
15-China_Henan-58	2015	Henan	OM453217	A	OM452540	A	OM451852	D
15-China_Henan-61	2015	Henan	OM453218	C	OM452541	C	OM451853	A
15-China_Henan-62	2015	Henan	OM453219	C	OM452542	C	OM451854	C
15-China_Henan-68	2015	Henan	OM453220	C	OM452543	C	OM451855	C
15-China_Henan-7	2015	Henan	OM453221	C	OM452544	C	OM451856	C
15-China_Henan-73	2015	Henan	OM453222	C	OM452545	C	OM451857	C
15-China_Henan-75	2015	Henan	OM453223	C	OM452546	C	OM451858	C
15-China_Henan-78	2015	Henan	OM453224	D	OM452547	D	OM451859	C
15-China_Henan-79	2015	Henan	OM453225	C	OM452548	C	OM451860	D
15-China_Henan-8	2015	Henan	OM453226	C	OM452549	C	OM451861	C
15-China_Henan-82	2015	Henan	OM453227	C	OM452550	C	OM451862	C
15-China_Henan-9	2015	Henan	OM453228	C	OM452551	C	OM451863	C
15-China_Henan-91	2015	Henan	OM453229	A	OM452552	A	OM451864	C
15-China_Henan-95	2015	Henan	OM453230	C	OM452553	C	OM451865	A
15-China_Hubei-154	2015	Henan	MN509930	C	MN510102	C	OM451866	C
15-China_Hubei-234	2015	Henan	MN509935	C	MN510103	D	MN510244	C
15-China_Hubei-259	2015	Henan	MN509938	C	MN510105	C	MN510246	C
16-China_Henan-110	2016	Henan	OM453154	D	OM452475	D	OM451782	D
16-China_Henan-115	2016	Henan	OM453231	A	OM452554	A	OM451867	A
16-China_Henan-118	2016	Henan	OM453232	C	OM452555	C	OM451868	C
16-China_Henan-122	2016	Henan	OM453233	C	OM452556	C	OM451869	C
16-China_Henan-123	2016	Henan	OM453234	C	OM452557	C	OM451870	C
16-China_Henan-128	2016	Henan	OM453235	C	OM452558	C	OM451871	C
16-China_Henan-135	2016	Henan	MN509957	C	MN510108	C	MN510256	C
16-China_Henan-136	2016	Henan	OM453161	D	OM452483	D	OM451790	D
16-China_Henan-138	2016	Henan	MN509958	C	MN510109	C	MN510257	C
16-China_Henan-149	2016	Henan	MN509959	C	MN510110	C	MN510258	C
16-China_Henan-16	2016	Henan	OM453236	A	OM452559	A	OM451872	A
16-China_Henan-164	2016	Henan	OM453237	C	OM452560	C	OM451873	C

Isolate	Year	Location	L		M		S	
			Accession	Genotype	Accession	Genotype	Accession	Genotype
16-China_Henan-167	2016	Henan	OM453238	A	OM452561	A	MN510259	A
16-China_Henan-17	2016	Henan	MN509956	C	MN510111	C	MN510255	C
16-China_Henan-176	2016	Henan	MN509960	C	MN510112	C	MN510260	C
16-China_Henan-179	2016	Henan	OM453239	A	OM452562	A	MN510261	A
16-China_Henan-18	2016	Henan	OM453240	C	OM452563	C	OM451874	C
16-China_Henan-181	2016	Henan	OM453241	C	OM452564	C	OM451875	C
16-China_Henan-187	2016	Henan	OM453242	C	OM452565	C	OM451876	C
16-China_Henan-188	2016	Henan	OM453243	A	OM452566	A	OM451877	A
16-China_Henan-19	2016	Henan	OM453244	C	OM452567	C	OM451878	C
16-China_Henan-196	2016	Henan	OM453245	A	OM452568	A	MN510262	A
16-China_Henan-198	2016	Henan	MN509961	C	OM452569	C	OM451879	C
16-China_Henan-212	2016	Henan	OM453246	D	OM452570	D	OM451880	D
17-China_Hubei-102	2017	Hubei	OM453281	C	OM452603	C	OM451909	C
17-China_Hubei-120	2017	Hubei	OM453292	C	OM452612	C	OM451919	C
16-China_Henan-245	2016	Henan	MN509963	C	MN510113	C	MN510264	C
17-China_Hubei-144	2017	Hubei	OM453303	C	OM452623	C	OM451930	C
16-China_Henan-251	2016	Henan	OM453247	A	OM452571	A	OM451881	A
17-China_Hubei-25	2017	Hubei	OM453274	D	OM452572	C	MK301482	B
16-China_Henan-279	2016	Henan	OM453248	C	MK301481	B	OM451882	C
CB6	2016	Republic of Korea	MK301480	B	OM452573	C	OM451883	C
16-China_Henan-283	2016	Henan	OM453249	C	OM452574	C	OM451884	C
16-China_Henan-286	2016	Henan	OM453250	C	OM452575	C	OM451885	C
16-China_Henan-29	2016	Henan	OM453251	C	OM452576	A	MN510265	A
16-China_Henan-305	2016	Henan	OM453252	A	OM452577	C	OM451886	C
16-China_Henan-32	2016	Henan	OM453253	C	OM452578	A	MN510266	A
16-China_Henan-335	2016	Henan	OM453254	A	OM452579	A	MN510267	A
16-China_Henan-341	2016	Henan	OM453255	A	OM452530	D	OM451841	D
16-China_Henan-364	2016	Henan	OM453207	D	MN510115	E	OM451887	E
16-China_Henan-370	2016	Henan	MN509965	E	OM452580	D	OM451888	D
16-China_Henan-39	2016	Henan	OM453256	D	OM452581	A	MN510268	A
16-China_Henan-397	2016	Henan	OM453257	A	OM452582	A	OM451889	A
16-China_Henan-401	2016	Henan	OM453258	A	MN510116	C	MN510269	C
16-China_Henan-402	2016	Henan	MN509966	C	OM452583	A	OM451890	A
16-China_Henan-405	2016	Henan	OM453259	A	MN510117	C	MN510270	C
16-China_Henan-412	2016	Henan	MN509967	C	MN510118	C	MN510271	C
16-China_Henan-418	2016	Henan	MN509968	C	MN510119	C	OM451891	C
16-China_Henan-421	2016	Henan	MN509969	C	MN510120	C	OM451892	C
16-China_Henan-428	2016	Henan	MN509970	C	MN510121	C	MN510272	C

Isolate	Year	Location	L		M		S	
			Accession	Genotype	Accession	Genotype	Accession	Genotype
16-China_Henan-431	2016	Henan	MN509971	C	MN510122	C	OM451893	C
16-China_Henan-439	2016	Henan	MN509972	C	MN510123	C	MN510273	C
16-China_Henan-444	2016	Henan	MN509973	C	OM452584	A	MN510274	A
16-China_Henan-452	2016	Henan	OM453260	A	MN510124	D	OM451894	D
16-China_Henan-453	2016	Henan	MN509974	D	MN510125	C	MN510275	C
16-China_Henan-456	2016	Henan	MN509975	C	OM452586	A	MN510276	A
16-China_Henan-504	2016	Henan	OM453262	A	OM452587	C	OM451896	C
16-China_Henan-532	2016	Henan	OM453263	C	MN510126	C	MN510277	C
16-China_Henan-590	2016	Henan	OM453264	C	OM452588	A	MN510278	A
16-China_Henan-595	2016	Henan	OM453265	A	OM452589	D	OM451897	D
16-China_Henan-598	2016	Henan	OM453266	D	OM452590	C	OM451898	C
16-China_Henan-605	2016	Henan	OM453267	C	OM452591	C	OM451899	C
16-China_Henan-614	2016	Henan	OM453268	C	OM452592	C	OM451900	C
16-China_Henan-64	2016	Henan	OM453269	C	OM452593	A	OM451901	A
16-China_Henan-74	2016	Henan	OM453270	A	OM452594	D	OM451902	D
16-China_Henan-81	2016	Henan	OM453271	D	MN510128	C	MN510263	C
16-China_Hubei-203	2016	Henan	MN509962	C	OM452602	C	OM451908	C
17-China_Henan-101	2017	Henan	OM453280	C	OM452605	C	OM451912	C
17-China_Henan-107	2017	Henan	OM453284	C	MN510129	C	MN510285	C
17-China_Henan-110	2017	Henan	MN509982	C	MN510130	C	MN510286	C
17-China_Henan-112	2017	Henan	MN509983	C	OM452609	C	OM451915	C
17-China_Henan-113	2017	Henan	OM453288	C	OM452608	C	OM451914	C
17-China_Henan-114	2017	Henan	OM453287	C	MN510131	C	MN510287	C
17-China_Henan-116	2017	Henan	MN509984	C	OM452614	C	OM451921	C
17-China_Henan-128	2017	Henan	OM453294	C	OM452616	C	OM451923	C
17-China_Henan-13	2017	Henan	OM453296	C	OM452618	A	OM451925	A
17-China_Henan-136	2017	Henan	OM453298	A	OM452595	A	MN510289	A
17-China_Henan-138	2017	Henan	OM453272	A	OM452619	C	OM451926	C
17-China_Henan-139	2017	Henan	OM453299	C	MN510132	C	MN510290	C
17-China_Henan-142	2017	Henan	MN509986	C	MN510133	C	MN510291	C
17-China_Henan-143	2017	Henan	MN509987	C	OM452624	C	OM451931	C
17-China_Henan-147	2017	Henan	OM453304	C	OM452625	C	OM451932	C
17-China_Henan-148	2017	Henan	OM453305	C	OM452596	C	MN510292	C
17-China_Henan-154	2017	Henan	MN509988	C	OM452635	C	OM451942	C
17-China_Henan-163	2017	Henan	OM453316	C	OM452637	D	OM451944	D
17-China_Henan-165	2017	Henan	OM453318	D	OM452640	C	OM451949	C
17-China_Henan-177	2017	Henan	OM453323	C	OM452597	C	MN510293	C
17-China_Henan-178	2017	Henan	MN509989	C	OM452645	D	OM451954	D

Isolate	Year	Location	L		M		S	
			Accession	Genotype	Accession	Genotype	Accession	Genotype
17-China_Henan-190	2017	Henan	OM453330	D	OM452648	A	OM451957	A
17-China_Henan-194	2017	Henan	OM453333	A	MN510134	C	MN510294	C
17-China_Henan-215	2017	Henan	MN509990	C	MN510135	D	OM451903	D
17-China_Henan-218	2017	Henan	OM453273	D	OM452663	A	OM451972	A
17-China_Henan-241	2017	Henan	OM453346	A	OM452598	A	MN510295	A
17-China_Henan-242	2017	Henan	MN509991	A	OM452667	C	OM451976	C
17-China_Henan-255	2017	Henan	OM453349	C	MN510136	D	OM451904	D
17-China_Henan-257	2017	Henan	OM453275	D	OM452670	C	OM451979	C
17-China_Henan-261	2017	Henan	OM453352	C	OM452672	A	OM451981	A
17-China_Henan-264	2017	Henan	OM453354	A	OM452674	C	OM451983	C
17-China_Henan-269	2017	Henan	OM453356	C	OM452681	C	OM451988	C
17-China_Henan-281	2017	Henan	OM453363	C	OM452682	C	OM451989	C
17-China_Henan-285	2017	Henan	OM453364	C	OM452683	C	OM451990	C
17-China_Henan-286	2017	Henan	OM453365	C	OM452685	D	OM451991	D
17-China_Henan-289	2017	Henan	OM453367	D	MN510137	C	MN510296	C
17-China_Henan-290	2017	Henan	MN509992	C	OM452687	C	OM451994	C
17-China_Henan-293	2017	Henan	OM453370	C	OM452688	A	OM451995	A
17-China_Henan-299	2017	Henan	OM453371	A	OM452689	A	OM451996	A
17-China_Henan-304	2017	Henan	OM453372	A	OM452599	C	MN510297	C
17-China_Henan-317	2017	Henan	MN509993	C	OM452600	A	MN510298	A
17-China_Henan-320	2017	Henan	OM453276	A	OM452693	C	OM452000	C
17-China_Henan-321	2017	Henan	OM453376	C	OM452692	D	OM451999	D
17-China_Henan-322	2017	Henan	OM453375	D	OM452694	C	OM452001	C
17-China_Henan-325	2017	Henan	OM453377	C	OM452696	C	OM452003	C
17-China_Henan-33	2017	Henan	OM453379	C	MN510138	C	MN510299	C
17-China_Henan-337	2017	Henan	MN509994	C	OM452701	C	OM452007	C
17-China_Henan-343	2017	Henan	OM453383	C	MN510139	C	MN510280	C
17-China_Henan-35	2017	Henan	MN509976	C	OM452706	C	OM452012	C
17-China_Henan-354	2017	Henan	OM453388	C	MN510140	C	MN510300	C
17-China_Henan-356	2017	Henan	MN509995	C	OM452704	C	OM452010	C
17-China_Henan-36	2017	Henan	OM453386	C	MN510141	C	MN510301	C
17-China_Henan-365	2017	Henan	MN509996	C	OM452713	C	OM452018	C
17-China_Henan-367	2017	Henan	OM453394	C	OM452717	A	OM452022	A
17-China_Henan-42	2017	Henan	OM453398	A	OM452720	A	OM452025	A
17-China_Henan-47	2017	Henan	OM453401	A	MN510142	C	MN510281	C
17-China_Henan-51	2017	Henan	MN509977	C	OM452724	C	OM452028	C
17-China_Henan-54	2017	Henan	OM453405	C	MN510143	C	MN510282	C
17-China_Henan-64	2017	Henan	MN509978	C	OM452729	C	OM452033	C

Isolate	Year	Location	L		M		S	
			Accession	Genotype	Accession	Genotype	Accession	Genotype
17-China_Henan-67	2017	Henan	OM453410	C	OM452733	C	OM452036	C
17-China_Henan-72	2017	Henan	OM453414	C	MN510145	D	OM451905	D
17-China_Henan-76	2017	Henan	OM453277	D	OM452734	C	OM452037	C
17-China_Henan-79	2017	Henan	OM453415	C	MN510146	D	OM451906	D
17-China_Henan-84	2017	Henan	OM453278	D	OM452738	A	OM452041	A
17-China_Henan-87	2017	Henan	OM453418	A	OM452601	A	MN510283	A
17-China_Henan-91	2017	Henan	OM453279	A	OM452613	D	OM451918	C
18-China_Hubei-118	2018	Hubei	OM453291	C	OM452638	C	OM451920	D
18-China_Hubei-123	2018	Hubei	OM453293	D	OM452643	D	OM451945	C
18-China_Hubei-167	2018	Hubei	OM453319	C	OM452649	C	OM451952	D
18-China_Hubei-186	2018	Hubei	OM453327	D	MN510147	C	OM451958	C
18-China_Hubei-358	2018	Hubei	OM453391	C	OM452709	C	OM452015	C
18-China_Hubei-368	2018	Hubei	OM453395	C	OM452714	C	OM452019	C
18-China_Hubei-58	2018	Hubei	OM453406	D	LC730376	B	MN510284	C
JY1	2021	Japan	LC732568	B	MN510148	D	OM452029	D
17-China_Henan-94	2017	Henan	MN509980	C	OM452742	C	LC730375	B
17-China_Henan-96	2017	Henan	MN509981	D	OM452743	C	OM451907	D
17-China_Henan-98	2017	Henan	OM453421	C	MN510149	C	OM452043	C
17-China_Henan-99	2017	Henan	OM453422	C	MN510150	D	OM452044	C
17-China_Hubei-125	2017	Henan	MN509985	C	MN510151	C	MN510288	C
17-China_Hubei-385	2017	Henan	MN509997	C	MN510152	C	MN510279	D
18-China_Henan-103	2018	Henan	OM453282	C	OM452604	A	MN510302	C
18-China_Henan-105	2018	Henan	OM453283	A	OM452606	C	OM451910	C
18-China_Henan-109	2018	Henan	OM453285	C	OM452607	A	OM451911	A
18-China_Henan-110	2018	Henan	OM453286	A	OM452610	D	MN510310	C
18-China_Henan-114	2018	Henan	OM453289	D	OM452611	A	OM451913	A
18-China_Henan-115	2018	Henan	OM453290	A	OM452615	A	OM451916	D
18-China_Henan-129	2018	Henan	OM453295	A	OM452617	C	OM451917	A
18-China_Henan-130	2018	Henan	OM453297	C	OM452620	C	OM451922	A
18-China_Henan-139	2018	Henan	OM453300	C	OM452621	C	OM451924	C
18-China_Henan-141	2018	Henan	OM453301	C	OM452622	C	OM451927	C
18-China_Henan-143	2018	Henan	OM453302	C	OM452626	C	OM451928	C
18-China_Henan-149	2018	Henan	OM453306	C	OM452627	C	OM451929	C
18-China_Henan-150	2018	Henan	OM453307	C	OM452628	A	OM451933	C
18-China_Henan-152	2018	Henan	OM453308	A	MN510155	D	OM451934	C
18-China_Henan-153	2018	Henan	OM453309	D	OM452629	C	MN510311	A
18-China_Henan-154	2018	Henan	OM453310	C	OM452630	A	OM451935	D
18-China_Henan-155	2018	Henan	OM453311	A	OM452631	A	OM451936	C

Isolate	Year	Location	L		M		S	
			Accession	Genotype	Accession	Genotype	Accession	Genotype
18-China_Henan-158	2018	Henan	OM453312	A	OM452632	C	OM451937	A
18-China_Henan-159	2018	Henan	OM453313	C	OM452633	C	OM451938	A
18-China_Henan-160	2018	Henan	OM453314	C	OM452634	C	OM451939	C
18-China_Henan-161	2018	Henan	OM453315	C	OM452636	D	OM451940	C
18-China_Henan-164	2018	Henan	OM453317	D	MN510156	C	OM451941	C
18-China_Henan-169	2018	Henan	OM453320	C	OM452639	C	OM451943	D
18-China_Henan-170	2018	Henan	OM453321	C	MN510157	D	OM451946	C
18-China_Henan-172	2018	Henan	OM453322	D	MN510159	C	OM451947	C
18-China_Henan-178	2018	Henan	OM453324	C	OM452641	C	OM451948	D
18-China_Henan-180	2018	Henan	OM453325	C	OM452642	C	OM451950	C
18-China_Henan-184	2018	Henan	OM453326	C	MN510160	C	OM451951	C
18-China_Henan-187	2018	Henan	OM453328	C	OM452644	C	MN510312	C
18-China_Henan-189	2018	Henan	OM453329	C	OM452646	C	OM451953	C
18-China_Henan-191	2018	Henan	OM453331	C	OM452647	C	MN510313	C
18-China_Henan-193	2018	Henan	OM453332	C	OM452650	D	OM451955	C
18-China_Henan-2	2018	Henan	OM453334	D	OM452651	A	OM451956	C
18-China_Henan-200	2018	Henan	OM453335	A	OM452652	C	OM451959	D
18-China_Henan-207	2018	Henan	OM453336	C	MN510162	D	MN510314	A
18-China_Henan-208	2018	Henan	MN510005	D	OM452653	C	OM451960	C
18-China_Henan-209	2018	Henan	MN510006	C	OM452654	C	OM451961	D
18-China_Henan-210	2018	Henan	OM453337	C	OM452655	C	OM451962	C
18-China_Henan-213	2018	Henan	OM453338	C	OM452656	C	OM451963	C
18-China_Henan-217	2018	Henan	OM453339	C	OM452657	A	MN510315	C
18-China_Henan-22	2018	Henan	OM453340	A	MN510163	D	OM451964	C
18-China_Henan-222	2018	Henan	OM453341	D	OM452658	C	OM451965	A
18-China_Henan-223	2018	Henan	OM453342	C	OM452659	C	OM451966	D
18-China_Henan-225	2018	Henan	OM453343	C	OM452660	C	OM451967	C
18-China_Henan-226	2018	Henan	OM453344	C	MN510164	D	OM451968	C
18-China_Henan-227	2018	Henan	MN510007	D	OM452661	C	MN510316	C
18-China_Henan-228	2018	Henan	OM453345	C	OM452662	D	OM451969	D
18-China_Henan-233	2018	Henan	MN510009	D	OM452664	A	OM451970	C
18-China_Henan-243	2018	Henan	OM453347	A	OM452665	C	OM451971	D
18-China_Henan-251	2018	Henan	OM453348	C	OM452666	C	OM451973	A
18-China_Henan-254	2018	Henan	MN510012	C	OM452668	C	OM451974	C
18-China_Henan-257	2018	Henan	OM453350	C	OM452669	C	OM451975	C
18-China_Henan-258	2018	Henan	OM453351	C	OM452671	C	OM451977	C
18-China_Henan-263	2018	Henan	OM453353	C	OM452673	D	OM451978	C
18-China_Henan-268	2018	Henan	OM453355	D	OM452675	C	OM451980	C

Isolate	Year	Location	L		M		S	
			Accession	Genotype	Accession	Genotype	Accession	Genotype
18-China_Henan-27	2018	Henan	OM453357	C	OM452676	D	OM451982	D
18-China_Henan-271	2018	Henan	OM453358	D	OM452677	C	OM451984	C
18-China_Henan-273	2018	Henan	OM453359	C	OM452678	A	OM451985	D
18-China_Henan-278	2018	Henan	OM453360	A	OM452679	C	OM451986	C
18-China_Henan-279	2018	Henan	OM453361	C	OM452680	A	MN510317	A
18-China_Henan-28	2018	Henan	OM453362	A	OM452684	A	MN510318	C
18-China_Henan-287	2018	Henan	OM453366	A	MN510168	D	OM451987	A
18-China_Henan-291	2018	Henan	OM453368	D	OM452686	A	MN510319	A
18-China_Henan-292	2018	Henan	OM453369	A	OM452690	C	OM451992	D
18-China_Henan-309	2018	Henan	OM453373	C	OM452691	C	OM451993	A
18-China_Henan-312	2018	Henan	OM453374	C	MN510170	C	OM451997	C
18-China_Henan-323	2018	Henan	MN510014	C	OM452695	C	OM451998	C
18-China_Henan-326	2018	Henan	OM453378	C	OM452697	C	MN510321	C
18-China_Henan-334	2018	Henan	OM453380	C	OM452698	C	OM452002	C
18-China_Henan-337	2018	Henan	MN510016	C	OM452699	C	MN510323	C
18-China_Henan-338	2018	Henan	OM453381	C	OM452700	C	OM452004	C
18-China_Henan-342	2018	Henan	OM453382	C	OM452702	C	OM452005	C
18-China_Henan-348	2018	Henan	OM453384	C	OM452745	C	OM452006	C
19-China_Hubei-144	2019	Hubei	OM453424	C	OM452873	D	OM452008	C
19-China_Hubei-166	2019	Hubei	OM453552	D	OM452747	C	OM452046	C
19-China_Hubei-167	2019	Hubei	OM453426	C	OM452875	C	OM452174	D
19-China_Hubei-174	2019	Hubei	OM453554	C	OM452880	C	OM452048	C
19-China_Hubei-185	2019	Hubei	OM453559	C	OM452882	C	OM452176	C
19-China_Hubei-195	2019	Hubei	OM453561	C	OM452755	D	OM452181	C
19-China_Hubei-238	2019	Hubei	OM453434	D	OM452896	C	OM452183	C
19-China_Hubei-240	2019	Hubei	OM453575	C	OM452900	C	OM452056	D
19-China_Hubei-249	2019	Hubei	OM453579	C	OM452907	C	OM452197	C
19-China_Hubei-263	2019	Hubei	OM453586	C	OM452915	C	OM452201	C
19-China_Hubei-279	2019	Hubei	OM453594	C	OM452918	D	OM452208	C
19-China_Hubei-291	2019	Hubei	OM453597	D	OM452920	C	OM452216	C
19-China_Hubei-297	2019	Hubei	OM453599	C	OM452922	C	OM452219	D
19-China_Hubei-299	2019	Hubei	OM453601	C	OM452927	C	OM452221	C
19-China_Hubei-306	2019	Hubei	OM453606	C	OM452929	C	OM452223	C
19-China_Hubei-310	2019	Hubei	OM453608	C	OM452930	C	OM452228	C
19-China_Hubei-314	2019	Hubei	OM453609	C	OM452935	D	OM452230	C
19-China_Hubei-327	2019	Hubei	OM453614	D	OM452937	C	OM452231	C
19-China_Hubei-377	2019	Hubei	OM453616	C	OM452765	C	OM452236	D
19-China_Hubei-7	2019	Hubei	OM453444	C	LC663821	B	OM452238	C

Isolate	Year	Location	L		M		S	
			Accession	Genotype	Accession	Genotype	Accession	Genotype
SFTSV/cat/1/Kochi/2021	2021	Japan	LC663822	B	LC663818	B	OM452066	C
SFTSV/human/1/Kochi/2021	2021	Japan	LC663819	B	OM452703	C	LC663820	B
18-China_Henan-349	2018	Henan	OM453385	C	OM452705	C	LC663817	B
18-China_Henan-353	2018	Henan	OM453387	C	OM452707	C	OM452009	C
18-China_Henan-356	2018	Henan	OM453389	C	OM452708	D	OM452011	C
18-China_Henan-357	2018	Henan	OM453390	D	OM452710	C	OM452013	C
18-China_Henan-362	2018	Henan	OM453392	C	OM452711	C	OM452014	D
18-China_Henan-365	2018	Henan	OM453393	C	OM452712	C	OM452016	C
18-China_Henan-366	2018	Henan	MN510018	C	OM452715	C	OM452017	C
18-China_Henan-37	2018	Henan	OM453396	C	OM452716	C	MN510325	C
18-China_Henan-40	2018	Henan	OM453397	C	OM452718	A	OM452020	C
18-China_Henan-43	2018	Henan	OM453399	A	OM452719	A	OM452021	C
18-China_Henan-44	2018	Henan	OM453400	A	OM452721	A	OM452023	A
18-China_Henan-50	2018	Henan	OM453402	A	OM452722	C	OM452024	A
18-China_Henan-51	2018	Henan	OM453403	C	OM452723	C	MN510304	A
18-China_Henan-53	2018	Henan	OM453404	C	OM452725	C	OM452026	C
18-China_Henan-6	2018	Henan	OM453407	C	MN510175	C	OM452027	C
18-China_Henan-60	2018	Henan	OM453408	C	OM452726	D	OM452030	C
18-China_Henan-61	2018	Henan	MN509998	D	OM452727	C	MN510305	C
18-China_Henan-64	2018	Henan	OM453409	C	OM452728	D	OM452031	D
18-China_Henan-65	2018	Henan	MN509999	D	OM452730	A	MN510306	C
18-China_Henan-7	2018	Henan	OM453411	A	OM452731	C	OM452032	D
18-China_Henan-70	2018	Henan	OM453412	C	OM452732	C	MN510303	A
18-China_Henan-71	2018	Henan	OM453413	C	OM452735	C	OM452034	C
18-China_Henan-80	2018	Henan	OM453416	C	OM452736	D	OM452035	C
18-China_Henan-81	2018	Henan	OM453417	D	OM452737	C	OM452038	C
18-China_Henan-84	2018	Henan	MN510000	C	OM452739	D	OM452039	D
18-China_Henan-89	2018	Henan	MN510001	D	OM452740	A	OM452040	C
18-China_Henan-93	2018	Henan	OM453419	A	MK301487	D	OM452042	D
CB8	2016	Republic of Korea	MK301486	D	OM452741	C	MN510308	A
18-China_Henan-94	2018	Henan	OM453420	C	MN510153	C	MK301488	D
18-China_Hubei-199	2018	Henan	MN510004	C	MN510174	D	MN510309	C
2020_P2_plasma/2020/China/Shandong	2020	Shandong	OP379658	D	OP379666	D	OP379674	D
2021_P11_plasma/2021/China/Shandong	2021	Shandong	OP379663	D	OP379671	D	OP379679	D
2018_154	2018	Henan	MW021165	C	MW021166	C	MW021167	C
19-China_Henan-12	2019	Henan	OM453423	C	OM452744	C	OM452045	C
2022_P28_plasma/2022/China/Shandong	2022	Shandong	OP379665	D	OP379673	D	OP379681	D
2022_P32_plasma/2022/China/Shandong	2022	Shandong	OP379664	D	OP379672	D	OP379680	D

Isolate	Year	Location	L		M		S	
			Accession	Genotype	Accession	Genotype	Accession	Genotype
19-China_Henan-126	2019	Henan	OM453543	D	OM452864	D	OM452165	D
19-China_Henan-129	2019	Henan	OM453544	A	OM452865	A	OM452166	A
19-China_Henan-132	2019	Henan	OM453545	C	OM452866	C	OM452167	C
19-China_Henan-133	2019	Henan	OM453546	C	OM452867	C	OM452168	C
19-China_Henan-137	2019	Henan	OM453547	C	OM452868	C	OM452169	C
19-China_Henan-139	2019	Henan	OM453548	C	OM452869	C	OM452170	C
19-China_Henan-148	2019	Henan	OM453549	C	OM452870	C	OM452171	C
19-China_Henan-156	2019	Henan	OM453550	A	OM452871	A	OM452172	A
19-China_Henan-160	2019	Henan	OM453551	C	OM452872	C	OM452173	C
19-China_Henan-162	2019	Henan	OM453425	D	OM452746	D	OM452047	D
19-China_Henan-173	2019	Henan	OM453553	A	OM452874	A	OM452175	A
19-China_Henan-175	2019	Henan	OM453555	C	OM452876	C	OM452177	C
19-China_Henan-177	2019	Henan	OM453556	C	OM452877	C	OM452178	C
19-China_Henan-179	2019	Henan	OM453557	A	OM452878	A	OM452179	A
19-China_Henan-183	2019	Henan	OM453558	C	OM452879	C	OM452180	C
19-China_Henan-186	2019	Henan	OM453427	C	OM452748	C	OM452049	C
19-China_Henan-189	2019	Henan	OM453560	C	OM452881	C	OM452182	C
19-China_Henan-19	2019	Henan	OM453428	D	OM452749	D	OM452050	D
19-China_Henan-193	2019	Henan	OM453429	C	OM452750	C	OM452051	C
19-China_Henan-197	2019	Henan	OM453562	C	OM452883	C	OM452184	C
19-China_Henan-198	2019	Henan	OM453563	C	OM452884	C	OM452185	C
19-China_Henan-199	2019	Henan	OM453564	C	OM452885	C	OM452186	C
19-China_Henan-201	2019	Henan	OM453565	C	OM452886	C	OM452187	C
19-China_Henan-208	2019	Henan	OM453566	C	OM452887	C	OM452188	C
19-China_Henan-214	2019	Henan	OM453567	C	OM452888	C	OM452189	C
19-China_Henan-215	2019	Henan	OM453568	A	OM452889	A	OM452190	A
19-China_Henan-217	2019	Henan	OM453569	C	OM452890	C	OM452191	C
19-China_Henan-223	2019	Henan	OM453570	A	OM452891	A	OM452192	A
19-China_Henan-227	2019	Henan	OM453571	A	OM452892	A	OM452193	A
19-China_Henan-231	2019	Henan	OM453430	C	OM452751	C	OM452052	C
19-China_Henan-232	2019	Henan	OM453572	C	OM452893	C	OM452194	C
19-China_Henan-233	2019	Henan	OM453431	D	OM452752	D	OM452053	D
19-China_Henan-234	2019	Henan	OM453432	A	OM452753	A	OM452054	A
19-China_Henan-235	2019	Henan	OM453573	C	OM452894	C	OM452195	C
19-China_Henan-236	2019	Henan	OM453574	C	OM452895	C	OM452196	C
19-China_Henan-237	2019	Henan	OM453433	C	OM452754	C	OM452055	C
19-China_Henan-241	2019	Henan	OM453576	C	OM452897	C	OM452198	C
19-China_Henan-242	2019	Henan	OM453577	C	OM452898	C	OM452199	C

Isolate	Year	Location	L		M		S	
			Accession	Genotype	Accession	Genotype	Accession	Genotype
19-China_Henan-243	2019	Henan	OM453578	C	OM452899	C	OM452200	C
19-China_Henan-248	2019	Henan	OM453435	C	OM452756	C	OM452057	C
19-China_Henan-253	2019	Henan	OM453580	C	OM452901	C	OM452202	C
19-China_Henan-254	2019	Henan	OM453581	C	OM452902	C	OM452203	C
19-China_Henan-259	2019	Henan	OM453582	C	OM452903	C	OM452204	C
19-China_Henan-260	2019	Henan	OM453583	C	OM452904	C	OM452205	C
19-China_Henan-261	2019	Henan	OM453584	C	OM452905	C	OM452206	C
19-China_Henan-262	2019	Henan	OM453585	C	OM452906	C	OM452207	C
19-China_Henan-264	2019	Henan	OM453587	C	OM452908	C	OM452209	C
19-China_Henan-266	2019	Henan	OM453588	C	OM452909	C	OM452210	C
19-China_Henan-268	2019	Henan	OM453589	C	OM452910	C	OM452211	C
19-China_Henan-271	2019	Henan	OM453436	A	OM452757	A	OM452058	A
19-China_Henan-272	2019	Henan	OM453590	C	OM452911	C	OM452212	C
19-China_Henan-273	2019	Henan	OM453591	C	OM452912	C	OM452213	C
19-China_Henan-274	2019	Henan	OM453592	D	OM452913	D	OM452214	D
19-China_Henan-278	2019	Henan	OM453593	C	OM452914	C	OM452215	C
19-China_Henan-280	2019	Henan	OM453595	A	OM452916	A	OM452217	A
19-China_Henan-285	2019	Henan	OM453596	C	OM452917	C	OM452218	C
19-China_Henan-287	2019	Henan	OM453437	A	OM452758	A	OM452059	A
19-China_Henan-295	2019	Henan	OM453598	D	OM452919	D	OM452220	D
19-China_Henan-298	2019	Henan	OM453600	C	OM452921	C	OM452222	C
19-China_Henan-3	2019	Henan	OM453438	C	OM452759	C	OM452060	C
19-China_Henan-301	2019	Henan	OM453602	C	OM452923	C	OM452224	C
19-China_Henan-302	2019	Henan	OM453603	C	OM452924	C	OM452225	C
19-China_Henan-304	2019	Henan	OM453604	C	OM452925	C	OM452226	C
19-China_Henan-305	2019	Henan	OM453605	C	OM452926	C	OM452227	C
19-China_Henan-308	2019	Henan	OM453607	A	OM452928	A	OM452229	A
19-China_Henan-309	2019	Henan	OM453439	C	OM452760	C	OM452061	C
19-China_Henan-313	2019	Henan	OM453440	C	OM452761	C	OM452062	C
19-China_Henan-316	2019	Henan	OM453441	A	OM452762	A	OM452063	A
19-China_Henan-317	2019	Henan	OM453442	C	OM452763	C	OM452064	C
19-China_Henan-321	2019	Henan	OM453610	A	OM452931	A	OM452232	A
19-China_Henan-322	2019	Henan	OM453611	C	OM452932	C	OM452233	C
19-China_Henan-323	2019	Henan	OM453612	C	OM452933	C	OM452234	C
19-China_Henan-324	2019	Henan	OM453613	D	OM452934	D	OM452235	D
19-China_Henan-330	2019	Henan	OM453443	C	OM452764	C	OM452065	C
19-China_Henan-358	2019	Henan	OM453615	C	OM452936	C	OM452237	C
19-China_Henan-382	2019	Henan	OM453617	D	OM452938	D	OM452239	D

Isolate	Year	Location	L		M		S	
			Accession	Genotype	Accession	Genotype	Accession	Genotype
19-China_Henan-388	2019	Henan	OM453618	C	OM452939	C	OM452240	C
19-China_Henan-94	2019	Henan	OM453445	C	OM452766	C	OM452067	C
2020_168	2020	Henan	MW021168	C	MW021169	C	MW021170	C
20-China_Henan-1	2020	Henan	OM453446	C	OM452767	C	OM452068	C
20-China_Henan-100	2020	Henan	OM453447	A	OM452768	A	OM452069	A
20-China_Henan-102	2020	Henan	OM453448	A	OM452769	A	OM452070	A
20-China_Henan-108	2020	Henan	OM453450	C	OM452771	C	OM452072	C
20-China_Henan-111	2020	Henan	OM453451	D	OM452772	C	OM452073	C
20-China_Henan-112	2020	Henan	OM453452	C	OM452773	C	OM452074	C
20-China_Henan-113	2020	Henan	OM453453	C	OM452774	C	OM452075	C
20-China_Henan-114	2020	Henan	OM453454	C	OM452775	C	OM452076	C
20-China_Henan-115	2020	Henan	OM453620	D	OM452941	D	OM452242	D
20-China_Henan-118	2020	Henan	OM453456	C	OM452777	C	OM452078	C
20-China_Henan-12	2020	Henan	OM453619	D	OM452940	D	OM452241	D
20-China_Henan-120	2020	Henan	OM453457	C	OM452778	C	OM452079	C
20-China_Henan-122	2020	Henan	OM453458	A	OM452779	A	OM452080	A
20-China_Henan-126	2020	Henan	OM453459	C	OM452780	C	OM452081	C
20-China_Henan-13	2020	Henan	OM453460	C	OM452781	C	OM452082	C
20-China_Henan-130	2020	Henan	OM453461	C	OM452782	C	OM452083	C
20-China_Henan-133	2020	Henan	OM453462	A	OM452783	C	OM452084	C
20-China_Henan-134	2020	Henan	OM453463	C	OM452784	C	OM452085	C
20-China_Henan-137	2020	Henan	OM453464	C	OM452785	C	OM452086	C
20-China_Henan-138	2020	Henan	OM453465	C	OM452786	C	OM452087	C
20-China_Henan-139	2020	Henan	OM453621	D	OM452942	D	OM452243	D
20-China_Henan-14	2020	Henan	OM453466	B	OM452787	B	OM452088	B
20-China_Henan-140	2020	Henan	OM453467	D	OM452788	D	OM452089	D
20-China_Henan-142	2020	Henan	OM453468	A	OM452789	A	OM452090	A
20-China_Henan-144	2020	Henan	OM453470	A	OM452791	A	OM452092	A
20-China_Henan-145	2020	Henan	OM453471	A	OM452792	A	OM452093	A
20-China_Henan-149	2020	Henan	OM453472	D	OM452793	D	OM452094	D
20-China_Henan-15	2020	Henan	OM453473	C	OM452794	C	OM452095	C
20-China_Henan-153	2020	Henan	OM453474	C	OM452795	C	OM452096	C
20-China_Henan-156	2020	Henan	OM453475	A	OM452796	A	OM452097	A
20-China_Henan-157	2020	Henan	OM453476	C	OM452797	C	OM452098	C
20-China_Henan-158	2020	Henan	OM453477	A	OM452798	A	OM452099	A
20-China_Henan-168	2020	Henan	OM453478	C	OM452799	C	OM452100	C
20-China_Henan-175	2020	Henan	OM453479	C	OM452800	C	OM452101	C
20-China_Henan-177	2020	Henan	OM453480	A	OM452801	A	OM452102	A

Isolate	Year	Location	L		M		S	
			Accession	Genotype	Accession	Genotype	Accession	Genotype
20-China_Hubei-116	2020	Hubei	OM453455	C	OM452776	C	OM452077	C
20-China_Hubei-143	2020	Hubei	OM453469	C	OM452790	C	OM452091	C
20-China_Hubei-191	2020	Hubei	OM453488	C	OM452809	D	OM452110	D
20-China_Hubei-201	2020	Hubei	OM453493	C	OM452814	C	OM452115	C
20-China_Hubei-228	2020	Hubei	OM453502	C	OM452823	C	OM452124	C
20-China_Hubei-233	2020	Hubei	OM453504	C	OM452825	A	OM452126	C
20-China_Hubei-240	2020	Hubei	OM453509	C	OM452830	C	OM452131	C
20-China_Hubei-54	2020	Hubei	OM453527	C	OM452848	C	OM452149	C
20-China_Hubei-92	2020	Hubei	OM453540	C	OM452861	C	OM452162	C
20-China_Jiangxi-104	2020	Jiangxi	OM453449	A	OM452770	C	OM452071	C
20ZJTZ01	2020	Zhejiang	MZ773019	B	MZ773034	B	MZ773049	B
20ZJTZ02	2020	Zhejiang	MZ773018	A	MZ773033	A	MZ773048	A
20ZJTZ03	2020	Zhejiang	MZ773029	B	MZ773044	B	MZ773059	B
20ZJTZ04	2020	Zhejiang	MZ773028	B	MZ773043	B	MZ773058	B
20ZJTZ05	2020	Zhejiang	MZ773027	A	MZ773042	A	MZ773057	A
20ZJTZ06	2020	Zhejiang	MZ773026	A	MZ773041	A	MZ773056	A
20ZJTZ07	2020	Zhejiang	MZ773025	A	MZ773040	A	MZ773055	A
20ZJTZ08	2020	Zhejiang	MZ773024	A	MZ773039	A	MZ773054	A
20ZJTZ09	2020	Zhejiang	MZ773023	A	MZ773038	A	MZ773053	A
20ZJTZ10	2020	Zhejiang	MZ773022	A	MZ773037	A	MZ773052	A
20ZJTZ11	2020	Zhejiang	MZ773021	A	MZ773036	A	MZ773051	A
20ZJTZ12	2020	Zhejiang	MZ773020	B	MZ773035	B	MZ773050	B
20ZJTZ13	2020	Zhejiang	MZ773017	A	MZ773032	A	MZ773045	A
20ZJTZ14	2020	Zhejiang	MZ773016	A	MZ773031	A	MZ773047	A
20ZJTZ15	2020	Zhejiang	MZ773015	A	MZ773030	A	MZ773046	A
20-China_Henan-178	2020	Henan	OM453481	A	OM452802	A	OM452103	A
20-China_Henan-180	2020	Henan	OM453482	C	OM452803	A	OM452104	A
20-China_Henan-181	2020	Henan	OM453483	C	OM452804	C	OM452105	C
20-China_Henan-182	2020	Henan	OM453484	C	OM452805	C	OM452106	C
20-China_Henan-183	2020	Henan	OM453485	C	OM452806	C	OM452107	C
20-China_Henan-189	2020	Henan	OM453486	C	OM452807	C	OM452108	C
1981_TKY	2019	Japan	LC549339	B	LC549338	B	LC549337	B
15KS10	2015	Republic of Korea	MG737017	B	MG737126	B	MG737234	B
AH001	2024	Anhui	PQ619853	C	PQ619852	C	PQ619854	C
AH12/China/2010AH12	2010	Anhui	HQ116417	C	HQ141590	C	HQ141591	C
AH15/China/2010AH15	2010	Anhui	HQ141592	A	HQ141593	A	HQ141594	A
AH-2019-CKY	2023	Anhui	OR709728	C	OR709720	C	OR709711	C
AH-2019-CZY	2019	Anhui	OR682385	C	OR682386	C	OR682387	C

Isolate	Year	Location	L		M		S	
			Accession	Genotype	Accession	Genotype	Accession	Genotype
AH-2019-QQS	2023	Anhui	OR709729	C	OR709721	C	OR709712	C
AH-2019-SDR	2023	Anhui	OR709730	C	OR709722	D	OR709713	D
AH-2019-TCY	2023	Anhui	OR709731	C	OR709723	C	OR709714	C
AH-2019-WDG	2019	Anhui	PP318212	A	OR682388	A	OR682389	A
AH-2019-ZSX	2023	Anhui	OR709732	B	OR709724	B	OR709715	B
AH-2019-ZXC	2019	Anhui	PP318213	D	OR682392	C	OR682393	D
AHL/China/2011	2011	Anhui	JQ670934	A	JQ670930	A	JQ670932	D
AHZ/2020-01	2020	Anhui	MT522608	C	MT522609	C	MT559337	C
AHZ/China/2011	2011	Anhui	JQ670929	C	JQ670931	C	JQ670933	C
AHZ-2019-22	2019	Anhui	OP729250	C	OP729249	C	OP729244	C
AHZ-2019-BJY	2019	Anhui	OP651053	C	OP699668	C	OP729240	C
AHZ-2019-LCL	2019	Anhui	OP651048	B	OP651049	B	OP651050	B
AHZ-2019-LCLin	2019	Anhui	OP651051	C	OP699664	C	OP699665	C
AHZ-2019-WGL	2019	Anhui	OP651055	C	OP729246	C	OP729242	C
AHZ-2019-WJ	2019	Anhui	PP318214	C	OR682394	C	OR709709	C
AHZ-2019-WXP	2019	Anhui	OP651054	C	OP729245	C	OP729241	C
AHZ-2019-ZYT	2019	Anhui	OP651056	B	OP729247	B	OP729243	B
AHZ-2022-CGY	2022	Anhui	OR762192	C	OR762193	C	OR762194	C
AHZ-2022-FDY	2022	Anhui	OR762207	A	OR762208	A	PP072285	A
AHZ-2022-GDK	2022	Anhui	OR762186	C	OR762187	C	OR762188	C
AHZ-2022-GSY	2022	Anhui	OR762189	C	OR762190	C	OR762191	C
AHZ-2022-HAF	2022	Anhui	OR762212	B	OR762213	B	OR762214	B
AHZ-2022-HAL	2022	Anhui	OR725097	C	OR725098	C	OR725099	C
AHZ-2022-HBY	2022	Anhui	OR762201	B	OR762202	A	OR762203	B
AHZ-2022-HZM	2022	Anhui	OR725100	C	OR725101	C	OR725102	C
AHZ-2022-JZH	2022	Anhui	OR725103	C	OR725104	C	OR725105	C
AHZ-2022-LJX	2022	Anhui	OR725106	C	OR725107	C	OR725108	C
AHZ-2022-LMW	2022	Anhui	OR762195	C	OR762196	C	OR762197	C
AHZ-2022-LXY	2022	Anhui	OR709733	C	OR709725	C	OR709716	C
AHZ-2022-WAX	2022	Anhui	OR762198	C	OR762199	C	OR762200	C
AHZ-2022-WFC	2022	Anhui	OR762204	B	OR762205	B	OR762206	B
AHZ-2022-WXC	2022	Anhui	OR762183	C	OR762184	C	OR762185	C
AHZ-2022-XJM	2022	Anhui	OR709735	C	OR709727	C	OR709718	C
AHZ-2022-ZRS	2022	Anhui	OR709734	C	OR709726	C	OR709717	C
AHZ-2022-ZXD	2022	Anhui	OR725109	C	OR725110	C	OR725111	C
AHZ-2022-ZXM	2022	Anhui	OR762209	B	OR762210	B	OR762211	B
AHZ-2023-CXY	2023	Anhui	PP072289	C	PP072290	C	PP072291	C
AHZ-2023-XYA	2023	Anhui	PP072292	A	PP072293	A	PP072294	D

Isolate	Year	Location	L		M		S	
			Accession	Genotype	Accession	Genotype	Accession	Genotype
AHZ-2023-ZYY	2023	Anhui	PP072286	A	PP072287	A	PP072288	A
AJH24006	2024	Zhejiang	PX306835	B	PX306828	B	PX306821	B
AJH24036	2024	Zhejiang	PX306836	A	PX306829	A	PX306822	A
AJH24047	2024	Zhejiang	PX306837	B	PX306830	B	PX306823	B
AJH25009	2025	Zhejiang	PX306838	B	PX306831	B	PX306824	B
AJH25015	2025	Zhejiang	PX306839	B	PX306832	B	PX306825	B
AJT24071	2024	Zhejiang	PX306840	B	PX306833	B	PX306826	B
AJT24074	2024	Zhejiang	PX306841	B	PX306834	B	PX306827	B
N100	2019	Japan	LC570801	B	LC570800	B	LC570799	B
15KS15	2015	Republic of Korea	MG920819	B	MG920820	B	MG920821	B
15KS16	2015	Republic of Korea	MG737020	B	MG737129	B	MG737237	B
15KS19	2015	Republic of Korea	MG737021	B	MG737130	B	MG737238	B
15KS20	2015	Republic of Korea	MG737022	B	MG737131	B	MG737239	B
15KS23	2015	Republic of Korea	MG737023	B	MG737132	B	MG737240	B
15KS25	2015	Republic of Korea	MG737024	B	MG737133	B	MG737241	B
15KS26	2015	Republic of Korea	MG737025	B	MG737134	B	MG737242	B
Ydog59	2019	Japan	LC570792	B	LC570791	B	LC570790	B
BJ001-2021	2021	Beijing	OQ832099	D	OQ832098	D	OQ832097	D
BJ2023-PG-003	2023	Beijing	OR679090	C	OR679087	C	OR679084	C
BJ2023-PG-004	2023	Beijing	OR679091	C	OR679088	C	OR679085	C
BJ2023-PG-Camel	2023	Beijing	OR679092	C	OR679089	C	OR679086	C
BK2947	2020	Thailand	OQ341225	B	OQ341221	B	ON840549	B
BK3247	2020	Thailand	OQ341226	B	OQ341222	B	ON840550	B
20-China_Henan-190	2020	Henan	OM453487	C	OM452808	A	OM452109	A
20-China_Henan-192	2020	Henan	OM453489	C	OM452810	C	OM452111	C
BNY19003	2019	Zhejiang	PP488489	B	PP488482	B	PP488475	B
BNY20005	NA	Zhejiang	PP488490	D	PP488483	D	PP488476	D
BNY20006	NA	Zhejiang	PP488491	D	PP488484	D	PP488477	D
BNY21002	2021	Zhejiang	PP488492	B	PP488485	A	PP488478	B
BNY22005	2022	Zhejiang	PP488493	D	PP488486	D	PP488479	D
BNY23017	2023	Zhejiang	PP488494	D	PP488487	D	PP488480	D
BNY23028	2023	Zhejiang	PP488495	A	PP488488	A	PP488481	A
20-China_Henan-198	2020	Henan	OM453490	C	OM452811	C	OM452112	C
20-China_Henan-20	2020	Henan	OM453491	A	OM452812	A	OM452113	A
20-China_Henan-200	2020	Henan	OM453492	C	OM452813	C	OM452114	C
Ydog63	2019	Japan	LC570795	B	LC570794	B	LC570793	B
15KS28	2015	Republic of Korea	MG737026	B	MG737135	B	MG737243	B
15KS29	2015	Republic of Korea	MG737027	B	MG737136	B	MG737244	B

Isolate	Year	Location	L		M		S	
			Accession	Genotype	Accession	Genotype	Accession	Genotype
15KS31	2015	Republic of Korea	MG737028	B	MG737137	B	MG737245	B
15KS33	2015	Republic of Korea	MG737029	B	MG737138	B	MG737246	B
15KS43	2015	Republic of Korea	MG920825	B	MG920826	B	MG920827	B
15KS47	2015	Republic of Korea	MG920828	B	MG920829	B	MG920830	B
15KS5	2015	Republic of Korea	MG737014	B	MG737123	B	MG737231	B
15KS50	2015	Republic of Korea	MG737030	B	MG737139	B	MG737247	B
15KS51	2015	Republic of Korea	MG921166	B	MG921167	B	MG921168	B
15KS57	2015	Republic of Korea	MG737033	A	MG737142	A	MG737250	A
15KS59	2015	Republic of Korea	MG737034	B	MG737143	B	MG737251	B
15KS61	2015	Republic of Korea	MG737035	B	MG737144	B	KR698319	B
CPF	2023	Anhui	PQ671011	C	PQ671010	C	PQ671009	C
15KS62	2015	Republic of Korea	MG737036	B	MG737145	B	MG737252	B
DanDong202	2016	Liaoning	MF357027	C	MF357013	C	MF357020	C
DanDong203	2016	Liaoning	MF357028	D	MF357014	D	MF357021	D
DanDong273	2016	Liaoning	MF357029	D	MF357015	B	MF357022	D
DanDong292	2016	Liaoning	MF357030	C	MF357016	C	MF357023	C
15KS64	2015	Republic of Korea	MG737037	A	MG737146	A	MG737253	B
dongchongjun-SFTSV	2024	Liaoning	PV553243	A	PV553242	A	PV553241	A
DS02/CHN/2013	2013	Zhejiang	KR698345	B	KR698332	B	KR698320	B
DS03/CHN/2013	2013	Zhejiang	KR698346	B	KR698333	B	KR698321	B
DS04/CHN/2013	2013	Zhejiang	KR698347	B	KR698334	B	KR698322	B
DS05/CHN/2013	2013	Zhejiang	KR698348	B	KR698335	B	PP706953	D
DZY	2024	Shandong	PQ463020	D	PQ463045	D	PQ463070	D
HZ2013-1	2013	Zhejiang	PP706825	D	PP706889	D	PP706954	B
15KS67	2015	Republic of Korea	MG737038	B	MG737147	B	MG737254	A
GAY	2023	Anhui	PQ671008	C	PQ671007	C	PQ671006	C
15KS71	2015	Republic of Korea	MG737039	B	MG737148	B	MG737255	B
20-China_Henan-203	2020	Henan	OM453494	A	OM452815	A	OM452116	A
Ydog70	2019	Japan	LC570798	A	LC570797	A	LC570796	A
HB154/China/2011	2011	Hubei	JQ733561	D	JQ733560	D	JQ733562	D
HB155/China/2011	2011	Hubei	JQ733564	D	JQ733563	D	JQ733565	D
HB156/China/2011	2011	Hubei	JQ733567	D	JQ733566	D	JQ733568	D
HB2011-004	2011	Hubei	MT114232	D	MT114246	D	MT114276	D
HB2011-071	2011	Hubei	MT114233	D	MT114247	C	MT114277	C
HB2011-137	2011	Hubei	MT114234	C	MT114248	C	MT114278	D
HB2012-171	2012	Hubei	MT114235	C	MT114249	C	MT114279	C
HB2012-175	2012	Hubei	MT114236	A	MT114250	A	MT114280	A
HB2012-177	2011	Hubei	MT114237	D	MT114251	D	MT114281	D

Isolate	Year	Location	L		M		S	
			Accession	Genotype	Accession	Genotype	Accession	Genotype
HB2012-179	2012	Hubei	MT114238	D	MT114252	D	MT114282	D
HB2012-182	2012	Hubei	MT114239	A	MT114253	A	MT114283	A
HB2012-193	2012	Hubei	MT114240	A	MT114254	A	MT114284	A
HB2012-196	2012	Hubei	MT114241	C	MT114255	C	MT114285	C
HB2012-197	2012	Hubei	MT114242	B	MT114256	A	MT114286	B
HB2013-048	2013	Hubei	MT114244	D	MT114258	D	MT114288	D
HB2013-050	2013	Hubei	MT114243	A	MT114257	A	MT114287	A
HB2014-013	2014	Hubei	MT114245	C	MT114259	C	MT114289	C
HB2014-04	2014	Hubei	KU524075	C	KR080475	C	KP984502	C
HB2014-08	2014	Hubei	KU529304	D	KY933682	D	KU738905	D
HB2014-10	2015	Hubei	KY933695	C	KY933683	C	KU738906	C
HB2014-12	2014	Hubei	KY933696	C	KY965128	C	KU738907	C
HB2014-13	2014	Hubei	KY933697	C	KY933684	C	KU738908	C
HB2014-15	2014	Hubei	KY933698	C	KY933685	C	KU738909	C
HB2014-20	2014	Hubei	KY933699	A	KY933686	A	KU738910	A
HB2014-21	2014	Hubei	KY933700	C	KY933687	C	KU738911	C
HB2014-22	2014	Hubei	KY933701	C	KY933688	C	KY933675	D
20-China_Henan-209	2020	Henan	OM453495	C	OM452816	C	OM452117	C
HB2014-31	2014	Hubei	KY933707	B	KY933691	B	KY933678	B
HB2014-33	2014	Hubei	KY933703	C	KY933690	C	KY933677	C
HB2014-35	2014	Hubei	KY933704	B	KY933692	B	KY933679	B
HB2014-36	2014	Hubei	KY933705	B	KY933693	B	KY933680	B
HB2014-37	2014	Hubei	KY933706	B	KY933694	B	KY933681	B
HB2015-09	2015	Hubei	MK513906	B	MK513905	B	MK513904	B
HB2015-10	2015	Hubei	MK513909	E	MK513908	E	MK513907	E
HB2015-19	2015	Hubei	MK513912	A	MK513911	A	MK513910	A
HB2015-23	2015	Hubei	MK513915	B	MK513914	B	MK513913	B
HB2015-25	2015	Hubei	MK513918	C	MK513917	C	MK513916	C
HB2015-28	2015	Hubei	MK513921	E	MK513920	E	MK513919	E
HB2015-31	2015	Hubei	MK513924	A	MK513923	A	MK513922	A
HB2015-34	2015	Hubei	MK513927	A	MK513926	A	MK513925	A
HB2015-35	2015	Hubei	MK513930	E	MK513929	E	MK513928	E
HB2015-39	2015	Hubei	MK513933	D	MK513932	D	MK513931	D
HB2016-003	2016	Hubei	KY965109	D	KY965092	D	KY965074	D
HB2016-010	2016	Hubei	KY965110	C	KY965127	C	KY965075	C
HB2016-011	2016	Hubei	KY965111	D	KY965093	D	KY965076	D
HB2016-013	2016	Hubei	KY965112	D	KY965094	D	KY965077	C
HB2016-016	2016	Hubei	KY965113	D	KY965095	D	KY965078	D

Isolate	Year	Location	L		M		S	
			Accession	Genotype	Accession	Genotype	Accession	Genotype
HB2016-021	2016	Hubei	KY965114	C	KY965096	C	KY965079	C
HB2016-031	2016	Hubei	KY965115	C	KY965097	C	KY965080	C
HB2016-034	2016	Hubei	KY965116	C	KY965098	C	KY965081	D
HB2016-038	2016	Hubei	KY965117	E	KY965099	E	KY965082	E
HB2016-047	2016	Hubei	KY965118	A	KY965100	A	KY965083	A
HB2016-052	2016	Hubei	KY965119	C	KY965101	C	KY965084	C
HB2016-055	2016	Hubei	KY965120	E	KY965102	E	KY965085	E
HB2016-073	2016	Hubei	KY965121	B	KY965103	B	KY965086	B
HB2016-078	2016	Hubei	KY965122	E	KY965104	E	KY965087	E
HB2016-080	2016	Hubei	KY965123	B	KY965105	B	KY965088	B
HB2016-090	2016	Hubei	KY965124	D	KY965106	D	KY965089	D
HB2016-100	2016	Hubei	KY965125	C	KY965107	C	KY965090	C
HB2016-106	2016	Hubei	KY965126	C	KY965108	C	KY965091	C
HB2016-P35	2016	Hubei	KY773990	C	KY773991	C	KY773992	C
HB2017-02	2017	Hubei	MK524353	D	MK524352	B	MK524351	D
HB2017-09	2017	Hubei	MK524356	C	MK524355	C	MK524354	C
HB2017-17	2017	Hubei	MK524359	B	MK524358	B	MK524357	B
HB2017-24	2017	Hubei	MK524362	C	MK524361	C	MK524360	C
HB2017-25	2017	Hubei	MK524365	D	MK524364	D	MK524363	D
HB2017-49	2017	Hubei	MK524368	C	MK524367	C	MK524366	C
HB2017-51	2017	Hubei	MK524371	E	MK524370	E	MK524369	E
HB2017-53	2017	Hubei	MK524374	C	MK524373	C	MK524372	C
HB2022-37/Hubei/2022	2022	Hubei	OQ388765	D	OQ388976	D	OQ389081	D
HB29	2010	Hubei	NC_018136	D	NC_018138	D	NC_018137	D
HZ2013-2	2013	Zhejiang	PP706826	B	PP706890	B	KY440772	C
HBGS13	2016	Hubei	KY440774	C	LC462232	B	OQ389082	C
HBHG2019-09/Huanggang_Qichun/2019	2019	Hubei	OQ388741	C	KY440773	C	OQ389083	B
HBHG2019-10/Huanggang_Yingshan/2019	2019	Hubei	OQ388742	B	OQ388955	C	OQ389084	C
HBHG2020-01/Huanggang_Xishui/2020	2020	Hubei	OQ388784	C	OQ388956	B	OQ389085	B
HBHG2020-02/Huanggang_Qichun/2020	2020	Hubei	OQ388785	B	OQ388999	C	OQ389086	B
HBHG2020-03/Huanggang_Yingshan/2020	2020	Hubei	OQ388743	B	OQ388998	B	OQ389087	B
HBHG2020-08/Huanggang_Yingshan/2020	2020	Hubei	OQ388744	B	OQ388957	B	OQ389088	B
HBHG2020-11/Huanggang_Yingshan/2020	2020	Hubei	OQ388783	B	OQ388958	B	OQ389089	D
HBHG2020-12/Huanggang_Hongan/2020	2020	Hubei	OQ388745	D	OQ388997	B	OQ389090	B
HBHG2020-14/Huanggang_Yingshan/2020	2020	Hubei	OQ388782	B	OQ388959	D	OQ389091	B
HBHG2021-01/Huanggang_Qichun/2021	2021	Hubei	OQ388780	B	OQ388996	B	OQ389092	B
HBHG2021-02/Huanggang_Luotian/2021	2021	Hubei	OQ388747	B	OQ388993	B	OQ389093	D
HBHG2021-04/Huanggang_Wuxue/2021	2021	Hubei	OQ388748	C	OQ388961	B	OQ389098	B

Isolate	Year	Location	L		M		S	
			Accession	Genotype	Accession	Genotype	Accession	Genotype
HBHG2021-13/Huanggang_Yingshan/2021	2021	Hubei	OQ388749	E	OQ388962	C	OQ389095	B
HBHG2021-18/Huanggang_Yingshan/2021	2021	Hubei	OQ388778	B	OQ388963	E	OQ389096	C
HBHG2021-20/Huanggang_Qichun/2021	2021	Hubei	OQ388777	C	OQ388994	B	OQ389097	B
HBHG2021-W11/Huanggang_Yingshan/2021	2021	Hubei	OQ388750	B	OQ388974	C	OQ389094	E
HBHG2021-W3/Huanggang_Yingshan/2021	2021	Hubei	OQ388760	B	OQ388964	B	OQ389099	B
HBHG2022-12/Huanggang_Qichun/2022	2022	Hubei	OQ388757	B	OQ388980	B	OQ389100	D
HBHG2022-36/Huanggang_Luotian/2022	2022	Hubei	OQ388758	B	OQ388971	B	OQ389101	B
HBHG2022-39/Huanggang_Yingshan/2022	2022	Hubei	OQ388764	B	OQ388973	D	OQ389102	C
HBHG2022-40/Huanggang_Macheng/2022	2022	Hubei	OQ388763	C	OQ388977	B	OQ389103	C
HBHG2022-41/Huanggang_Yingshan/2022	2022	Hubei	OQ388762	C	OQ388981	C	MT320789	D
HBHG29	2017	Hubei	MT320787	D	OQ388978	C	MT320792	D
HBHG30	2017	Hubei	MT320790	D	MT320788	D	MT320795	D
HBHG35	2017	Hubei	MT320793	D	MT320791	D	MT320798	A
HBHG36	2017	Hubei	MT320796	A	MT320794	D	MT320801	D
HBHG38	2017	Hubei	MT320799	A	MT320797	A	MT320810	C
HBHG54	2017	Hubei	MT320808	C	MT320800	A	MT320816	D
HBHG8	2017	Hubei	MT320814	D	MT320809	C	KY440775	D
HBMC16	2015	Hubei	KY440777	D	MT320815	D	KY440769	D
HBMC5	2016	Hubei	KY440771	D	KY440776	D	PP239069	A
HBSY2023-1	2023	Hubei	PP239063	A	KY440770	D	PP239070	C
HBSY2023-3	2023	Hubei	PP239064	C	PP239066	C	PP239071	C
HBSY2023-4	2023	Hubei	PP239065	C	PP239067	C	PQ866003	C
HBSY2024-14	2024	Hubei	PQ865997	C	PP239068	C	PQ866004	C
HBSY2024-18	2024	Hubei	PQ865998	C	PQ866000	C	PQ866002	C
HBSY2024-4	2024	Hubei	PQ865996	C	PQ866001	C	MT320819	D
HBSZ11	2017	Hubei	MT320817	D	PQ865999	C	OQ389104	C
HBSZ2021-28/Suizhou/2021	2021	Hubei	OQ388753	C	MT320818	D	OQ389105	E
HBSZ2022-10/Suizhou/2022	2022	Hubei	OQ388755	E	OQ388966	C	OQ389106	C
HBSZ2022-11/Suizhou/2022	2022	Hubei	OQ388756	D	OQ388969	E	OQ389107	C
HBSZ2022-18/Suizhou/2022	2022	Hubei	OQ388773	C	OQ388970	C	OQ389108	C
HBSZ2022-22/Suizhou/2022	2022	Hubei	OQ388772	C	OQ388988	C	OQ389109	D
HBSZ2022-23/Suizhou/2022	2022	Hubei	OQ388771	D	OQ388987	C	OQ389110	C
HBSZ2022-25/Suizhou/2022	2022	Hubei	OQ388770	C	OQ388986	D	OQ389111	C
HBSZ2022-27/Suizhou/2022	2022	Hubei	OQ388752	C	OQ388985	C	OQ389112	E
HBSZ2022-28/Suizhou/2022	2022	Hubei	OQ388769	E	OQ388965	C	OQ389113	C
HBSZ2022-29/Suizhou/2022	2022	Hubei	OQ388768	C	OQ388984	E	OQ389114	D
HBSZ2022-30/Suizhou/2022	2022	Hubei	OQ388767	D	OQ388983	C	OQ389115	D
HBSZ2022-31/Suizhou/2022	2022	Hubei	OQ388766	D	OQ388975	D	OQ389116	D

Isolate	Year	Location	L		M		S	
			Accession	Genotype	Accession	Genotype	Accession	Genotype
HBSZ2022-54/Suizhou/2022	2022	Hubei	OQ388759	D	OQ388982	D	MT320807	C
HBSZ52	2017	Hubei	MT320805	C	OQ388972	C	MT320813	D
HBSZ55	2017	Hubei	MT320811	C	MT320806	C	OQ389117	C
HBWH2021-09/Wuhan_Xinzhou/2021	2021	Hubei	OQ388779	C	MT320812	C	OQ389118	D
HBXG2020-16/Xiaogan_Dawu/2020	2020	Hubei	OQ388781	D	OQ388992	C	OQ389119	A
HBXG2020-26/Xiaogan_Dawu/2020	2020	Hubei	OQ388746	A	OQ388995	D	OQ389120	D
HBXG2022-01/Xiaogan_Dawu/2022	2022	Hubei	OQ388776	D	OQ388960	A	OQ389121	D
HBXG2022-04/Xiaogan_Dawu/2022	2022	Hubei	OQ388775	D	OQ388991	D	OQ389122	D
HBXG2022-07/Xiaogan_Xiaochang/2022	2022	Hubei	OQ388751	D	OQ388990	D	OQ389123	D
HBXG2022-08/Xiaogan_Xiaochang/2022	2022	Hubei	OQ388774	D	OQ388967	D	OQ389124	A
HBXG2022-09/Xiaogan_Dawu/2022	2022	Hubei	OQ388754	A	OQ388989	D	MT320804	C
HBXG51	2017	Hubei	MT320802	C	OQ388968	A	OQ389125	C
HBYC2022-49/Yichang/2022	2022	Hubei	OQ388761	C	MT320803	C	PP761123	C
HBYS-2	2023	Hebei	PP761119	C	OQ388979	C	PP761124	C
HBYS-3	2023	Hebei	PP761120	C	PP761121	C	PQ463086	D
HCY	2024	Shandong	PQ463036	D	PP761122	C	PQ463071	D
HFX	2024	Shandong	PQ463021	D	PQ463061	D	MG737256	B
15KS72	2015	Republic of Korea	MG737040	B	PQ463046	D	MG737257	B
15KS73	2015	Republic of Korea	MG737041	C	MG737149	B	MG737258	C
15KS74	2015	Republic of Korea	MG737043	B	MG737150	C	MG737260	B
15KS75	2015	Republic of Korea	MG921169	B	MG737152	B	OM452118	A
20-China_Henan-210	2020	Henan	OM453496	A	MG921170	B	OM452119	C
20-China_Henan-211	2020	Henan	OM453497	C	OM452817	A	OM452120	D
20-China_Henan-215	2020	Henan	OM453498	D	OM452818	C	OM452121	C
20-China_Henan-219	2020	Henan	OM453499	C	OM452819	D	OM452122	C
20-China_Henan-222	2020	Henan	OM453500	C	OM452820	C	OM452123	A
20-China_Henan-223	2020	Henan	OM453501	A	OM452821	C	OM452125	A
20-China_Henan-230	2020	Henan	OM453503	A	OM452822	A	OM452127	C
20-China_Henan-234	2020	Henan	OM453505	C	OM452824	A	OM452128	C
20-China_Henan-236	2020	Henan	OM453506	C	OM452826	C	OM452129	A
20-China_Henan-238	2020	Henan	OM453507	A	OM452827	C	OM452130	C
20-China_Henan-24	2020	Henan	OM453508	A	OM452828	A	OM452132	C
20-China_Henan-244	2020	Henan	OM453510	C	OM452829	C	OM452133	C
20-China_Henan-246	2020	Henan	OM453511	C	OM452831	C	OM452134	C
20-China_Henan-249	2020	Henan	OM453512	C	OM452832	C	OM452135	A
20-China_Henan-252	2020	Henan	OM453513	A	OM452833	C	OM452136	D
20-China_Henan-254	2020	Henan	OM453514	D	OM452834	A	OM452137	D
20-China_Henan-255	2020	Henan	OM453515	D	OM452835	D	OM452138	D

Isolate	Year	Location	L		M		S	
			Accession	Genotype	Accession	Genotype	Accession	Genotype
20-China_Henan-28	2020	Henan	OM453516	D	OM452836	D	OM452139	C
20-China_Henan-3	2020	Henan	OM453517	C	OM452837	D	OM452140	C
20-China_Henan-37	2020	Henan	OM453518	C	OM452838	C	OM452141	C
20-China_Henan-4	2020	Henan	OM453519	C	OM452839	C	OM452142	C
20-China_Henan-40	2020	Henan	OM453520	C	OM452840	C	OM452143	C
20-China_Henan-41	2020	Henan	OM453521	C	OM452841	C	OM452144	C
20-China_Henan-42	2020	Henan	OM453522	C	OM452842	C	OM452145	C
20-China_Henan-43	2020	Henan	OM453523	C	OM452843	C	OM452146	C
20-China_Henan-48	2020	Henan	OM453524	C	OM452844	C	OM452147	C
20-China_Henan-49	2020	Henan	OM453525	C	OM452845	C	OM452148	C
20-China_Henan-51	2020	Henan	OM453526	C	OM452846	C	OM452150	C
20-China_Henan-59	2020	Henan	OM453528	C	OM452847	C	MH790412	C
Hubei	2016	Hubei	MH790414	C	OM452849	C	PP706956	A
HZ2014-1	2014	Zhejiang	PP706828	A	MH790413	C	PP706957	A
HZ2015-1	2015	Zhejiang	PP706829	A	PP706892	A	PP706958	E
HZ2017-1	2017	Zhejiang	PP706830	E	PP706893	A	PP706960	A
HZ2018-13	2018	Zhejiang	PP706832	A	PP706894	E	PP706961	A
HZ2018-15	2018	Zhejiang	PP706833	A	PP706896	A	PP706959	E
HZ2018-2	2018	Zhejiang	PP706831	E	PP706897	A	PP706962	C
HZ2018-23	2018	Zhejiang	PP706834	B	PP706895	E	PP706963	C
HZ2018-44	2018	Zhejiang	PP706835	C	PP706898	C	PP706964	C
HZ2018-46	2018	Zhejiang	PP706836	C	PP706899	C	PP706965	A
HZ2019-10	2019	Zhejiang	PP706837	A	PP706900	C	PP706966	A
HZ2019-32	2019	Zhejiang	PP706838	A	PP706901	A	PP706967	D
HZ2019-38	2019	Zhejiang	PP706839	D	PP706902	A	PP706969	B
HZ2020-2	2020	Zhejiang	PP706841	B	PP706903	D	PP706970	B
HZ2021-1	2021	Zhejiang	PP706842	B	PP706905	B	PP706971	B
HZ2021-4	2021	Zhejiang	PP706843	B	PP706906	B	PP706972	E
HZ2021-5	2021	Zhejiang	PP706844	E	PP706907	B	PP706973	A
HZ2021-6	2021	Zhejiang	PP706845	A	PP706908	E	PP706979	A
HZ2022-10	2022	Zhejiang	PP706851	A	PP706909	A	PP706980	A
HZ2022-11	2022	Zhejiang	PP706852	A	PP706915	A	PP706981	E
HZ2022-13	2022	Zhejiang	PP706853	E	PP706916	A	PP706974	B
HZ2022-2	2022	Zhejiang	PP706846	B	PP706917	E	PP706975	B
HZ2022-5	2022	Zhejiang	PP706847	B	PP706910	A	PP706977	A
HZ2022-7	2022	Zhejiang	PP706849	A	PP706911	B	PP706982	A
HZ2023-1	2023	Zhejiang	PP706854	A	PP706913	A	PP706988	D
HZ2023-10	2023	Zhejiang	PP706860	D	PP706918	A	PP706989	D

Isolate	Year	Location	L		M		S	
			Accession	Genotype	Accession	Genotype	Accession	Genotype
HZ2023-11	2023	Zhejiang	PP706861	D	PP706924	D	PP706990	D
HZ2023-12	2023	Zhejiang	PP706862	D	PP706925	D	PP706991	D
HZ2023-13	2023	Zhejiang	PP706864	D	PP706926	D	PP706993	D
HZ2023-15	2023	Zhejiang	PP706865	D	PP706927	D	PP706994	A
HZ2023-16	2023	Zhejiang	PP706866	A	PP706929	C	PP706995	A
HZ2023-17	2023	Zhejiang	PP706867	A	PP706930	A	PP706996	B
HZ2023-18	2023	Zhejiang	PP706868	B	PP706931	A	PP706997	D
HZ2023-19	2023	Zhejiang	PP706869	D	PP706932	B	PP706998	E
HZ2023-20	2023	Zhejiang	PP706870	E	PP706933	D	PP706999	B
HZ2023-21	2023	Zhejiang	PP706871	B	PP706934	E	PP707000	D
HZ2023-23	2023	Zhejiang	PP706872	D	PP706935	B	PP707001	A
HZ2023-24	2023	Zhejiang	PP706873	A	PP706936	D	PP707002	B
HZ2023-26	2023	Zhejiang	PP706874	B	PP706937	A	PP707003	B
HZ2023-27	2023	Zhejiang	PP706875	B	PP706938	B	PP707004	A
HZ2023-28	2023	Zhejiang	PP706876	A	PP706939	B	PP707005	A
HZ2023-29	2023	Zhejiang	PP706877	A	PP706940	A	PP706983	A
HZ2023-3	2023	Zhejiang	PP706855	A	PP706941	A	PP707006	A
HZ2023-30	2023	Zhejiang	PP706878	A	PP706919	A	PP707007	B
HZ2023-31	2023	Zhejiang	PP706879	B	PP706942	A	PP707008	D
HZ2023-32	2023	Zhejiang	PP706880	D	PP706943	B	PP707009	E
HZ2023-33	2023	Zhejiang	PP706881	E	PP706944	C	PP707010	C
HZ2023-34	2023	Zhejiang	PP706882	C	PP706945	E	PP707011	A
HZ2023-35	2023	Zhejiang	PP706883	A	PP706946	C	PP707012	A
HZ2023-36	2023	Zhejiang	PP706884	A	PP706947	A	PP707013	E
HZ2023-37	2023	Zhejiang	PP706885	E	PP706948	A	PP707014	C
HZ2023-38	2023	Zhejiang	PP706886	C	PP706949	E	PP707015	A
HZ2023-39	2023	Zhejiang	PP706887	A	PP706950	C	PP707016	A
HZ2023-40	2023	Zhejiang	PP706888	A	PP706951	A	PP706984	A
HZ2023-5	2023	Zhejiang	PP706856	A	PP706952	A	PP706985	A
HZ2023-6	2023	Zhejiang	PP706857	A	PP706920	A	PP706986	C
HZ2023-8	2023	Zhejiang	PP706858	C	PP706921	A	PP706987	C
HZ2023-9	2023	Zhejiang	PP706859	C	PP706922	C	KR698323	A
NB08/CHN/2013	2013	Zhejiang	KR698349	A	PP706923	C	KR698324	B
NB24/CHN/2013	2013	Zhejiang	KR698350	B	KR698336	A	MG921171	B
15KS77	2015	Republic of Korea	MG737044	B	KR698337	B	MG737261	B
15KS8	2015	Republic of Korea	MG737015	C	MG737153	B	MG737232	C
15KS9	2015	Republic of Korea	MG737016	B	MG737124	C	MG737233	B
15MS104	2015	Republic of Korea	MG737031	B	MG737125	B	MH822138	C

Isolate	Year	Location	L		M		S	
			Accession	Genotype	Accession	Genotype	Accession	Genotype
JA1-2018	2018	Jilin	MH822136	C	MG737140	B	LC462229	B
Cat/Miyazaki/2018	2018	Japan	LC462235	B	MH822137	C	LC462230	B
H9/Miyazaki/2018	2018	Japan	LC462236	B	LC462233	B	KT890282	A
JaOT2018-6	2018	Japan	LC590893	B	KT890281	A	MG737248	B
Jilin2013	2013	Jilin	KT890280	A	MG737151	B	MG737259	B
15MS132	2015	Republic of Korea	MG737042	B	MG737127	C	MG737235	C
15MS31	2015	Republic of Korea	MG737018	C	MG737128	B	MG737236	B
15MS32	2015	Republic of Korea	MG737019	B	MG920823	B	MG920824	B
15MS65	2015	Republic of Korea	MG920822	B	MG737141	B	MG737249	B
Asan_Serum1	2015	Republic of Korea	MG737032	B	KY789437	A	JN258705	D
CB2	2015	Republic of Korea	KY789434	A	JN258706	D	KY789440	A
JN1/China/2010	2010	Shandong	JN258707	D	MG737103	B	MG737211	B
14KS22	2014	Republic of Korea	MG736994	B	MG737104	B	MG737212	B
14KS25	2014	Republic of Korea	MG736995	B	MG737105	B	MG737213	B
14KS27	2014	Republic of Korea	MG736996	B	MG737106	B	MG737214	B
14KS30	2014	Republic of Korea	MG736997	B	MG737107	B	JF837595	A
14KS33	2014	Republic of Korea	MG736998	B	JF837594	A	JQ317171	C
JS2007-01	2011	Jiangsu	JF837593	A	JQ317170	C	JQ317174	C
JS2010-014	2010	Jiangsu	JQ317169	C	JQ317173	C	JQ317177	C
JS2010-015	2010	Jiangsu	JQ317172	C	JQ317176	C	JQ317180	C
JS2010-018	2010	Jiangsu	JQ317175	C	JQ317179	C	KC505125	A
JS2010-019	2011	Jiangsu	JQ317178	C	KC505124	A	KC505128	C
JS2011-004	2012	Jiangsu	KC505123	A	KC505127	C	KC505131	A
JS2011-013-1	2012	Jiangsu	KC505126	C	KC505130	A	KC505134	C
JS2011-027	2012	Jiangsu	KC505129	A	KC505133	C	KC505137	D
JS2011-034	2012	Jiangsu	KC505132	C	KC505136	D	KY362318	A
JS2011-062	2012	Jiangsu	KC505135	D	KY362342	A	KC505140	A
JS2011-106	2011	Anhui	KY362294	A	KC505139	A	KR230790	A
JS2011-109	2012	Jiangsu	KC505138	A	KR230770	A	KY362319	A
JS2011-13-2	2011	Jiangsu	KR230750	A	KY362343	A	KY362320	A
JS2011-19	2011	Anhui	KY362295	A	KY362344	A	KY362321	A
JS2011-28	2011	Anhui	KY362296	A	KY362345	A	KR230791	B
JS2011-46	2011	Anhui	KY362297	A	KR230771	B	KY362322	C
JS2011-69	2011	Jiangsu	KR230751	B	KY362346	C	KY362323	A
JS2011-70	2011	Anhui	KY362298	C	KY362347	A	KY362324	C
JS2011-79	2011	Anhui	KY362299	A	KY362348	C	KY362325	C
JS2011-92	2011	Anhui	KY362300	C	KY362349	C	KC505143	A
JS2011-98	2011	Anhui	KY362301	C	KC505142	A	KC505146	A

Isolate	Year	Location	L		M		S	
			Accession	Genotype	Accession	Genotype	Accession	Genotype
JS2012-020	2012	Jiangsu	KC505141	A	KC505145	A	KY362326	A
JS2012-035	2012	Jiangsu	KC505144	A	KY362350	A	KR230792	C
JS2012-70	2012	Anhui	KY362302	A	KR230772	C	KC473539	C
JS2012-79	2012	Jiangsu	KR230752	C	KC473538	C	KC473542	C
JS2012-goat01	2012	Jiangsu	KC473537	C	KC473541	C	KY362327	E
JS2012-tick01	2012	Jiangsu	KC473540	C	KY362351	E	KY362335	C
JS2013-31	2013	Anhui	KY362303	E	KY362359	C	KY362336	C
JS2013-32	2013	Anhui	KY362311	C	KY362360	C	KY362337	C
JS2013-38	2013	Anhui	KY362312	C	KY362361	C	KR230793	C
JS2013-41	2013	Anhui	KY362313	C	KR230773	C	KR230794	A
JS2013-44	2013	Jiangsu	KR230753	C	KR230774	A	KR230795	C
JS2013-46	2013	Jiangsu	KR230754	A	KR230775	C	KY362328	C
JS2013-52	2013	Jiangsu	KR230755	C	KY362352	C	KR230796	C
JS2013-55	2013	Anhui	KY362304	C	KR230776	C	KR230797	A
JS2013-69	2013	Jiangsu	KR230756	C	KR230777	A	KR230798	C
JS2013-71	2013	Jiangsu	KR230757	A	KR230778	C	KY362338	C
JS2014-03	2014	Jiangsu	KR230758	C	KY362362	C	KY362339	C
JS2014-04	2014	Anhui	KY362314	C	KY362363	C	KR230799	A
JS2014-06	2014	Anhui	KY362315	C	KR230779	A	KR230800	B
JS2014-15	2014	Jiangsu	KR230759	A	KR230780	B	KR230801	A
JS2014-16	2014	Jiangsu	KR230760	B	KR230781	A	KY362329	A
JS2014-18	2014	Jiangsu	KR230761	A	KY362353	A	KR230802	C
JS2014-25	2014	Anhui	KY362305	A	KR230782	C	KR230803	C
JS2014-31	2014	Jiangsu	KR230762	C	KR230783	C	KR230804	A
JS2014-33	2014	Jiangsu	KR230763	C	KR230784	C	KR230805	B
JS2014-39	2014	Jiangsu	KR230764	C	KR230785	B	KR230806	C
JS2014-H.longicornis-01	2014	Jiangsu	KR230765	B	KR230786	C	KR230807	C
JS2014-H.longicornis-02	2014	Jiangsu	KR230766	C	KR230787	C	LC590895	B
JS2014-H.longicornis-03	2014	Jiangsu	KR230767	C	LC590894	B	LC733203	B
SFTSV/Dog/1/Thailand/2022	2022	Thailand	LC733205	B	LC733204	B	KY362340	A
Ydog34	2018	Japan	LC570786	A	KY362364	A	KY362330	C
JS2015-01	2015	Anhui	KY362316	A	KY362354	C	KY362341	D
JS2015-26	2015	Anhui	KY362306	C	KY362365	D	KY362331	C
JS2015-32	2015	Anhui	KY362317	D	KY362355	C	KY362332	B
JS2015-36	2015	Anhui	KY362307	C	KY362356	B	KY362333	C
JS2015-69	2015	Anhui	KY362308	B	KY362357	C	HQ830165	A
JS2015-78	2015	Anhui	KY362309	C	HQ830164	A	HQ830168	A
JS24	2010	Jiangsu	HQ830163	A	HQ830167	A	HQ141603	A

Isolate	Year	Location	L		M		S	
			Accession	Genotype	Accession	Genotype	Accession	Genotype
JS26	2010	Jiangsu	HQ830166	A	HQ141602	A	HQ141606	E
JS3/China/2010	2010	Jiangsu	HQ141601	A	HQ141605	E	HQ830171	A
JS4/China/2010	2010	Jiangsu	HQ141604	E	HQ830170	A	JF267785	C
JS6	2010	Jiangsu	HQ830169	A	JF267784	C	MN245798	D
JSD1	2011	Jiangsu	JF267783	C	MN245797	D	OR574963	B
JX2013-01	2013	Jiangxi	MN245796	D	OR574964	A	OR862770	B
JX23WN	2023	Jiangxi	OR574965	B	OR862771	A	LC570784	A
JX23WNZ	2023	Jiangxi	OR862772	B	LC570785	A	PQ463069	D
Ydog39	2018	Japan	LC570789	B	PQ463044	D	MG737215	B
JYJ	2023	Shandong	PQ463019	D	MG737108	C	MG737216	C
14KS35	2014	Republic of Korea	MG736999	C	MG737109	B	MG737217	B
14KS38	2014	Republic of Korea	MG737000	B	MG737110	C	MG737218	C
14KS39	2014	Republic of Korea	MG737001	C	MG737111	B	MG737219	B
14KS42	2014	Republic of Korea	MG737002	B	MG737112	B	MG737220	B
14KS44	2014	Republic of Korea	MG737003	B	MG737113	B	MG737221	B
14KS46	2014	Republic of Korea	MG737004	B	MG737114	C	MG737222	C
14KS47	2014	Republic of Korea	MG737005	C	MG737115	B	MG737223	B
14KS48,49	2014	Republic of Korea	MG737006	B	MG737116	A	MG737224	A
14KS52	2014	Republic of Korea	MG737007	A	MG737117	B	MG737225	B
14KS53,55	2014	Republic of Korea	MG737008	B	MG737118	D	MG737226	D
14KS58	2014	Republic of Korea	MG737009	D	MG737119	B	MG737227	B
14KS62	2014	Republic of Korea	MG737010	B	MG737120	D	MG737228	D
14KS64	2014	Republic of Korea	MG737011	D	MG737121	B	MG737229	B
14KS72	2014	Republic of Korea	MG737012	B	MG737122	B	MG737230	B
14KS73	2014	Republic of Korea	MG737013	B	KY789436	B	KY789439	B
CB1	2014	Republic of Korea	KY789433	B	KP663744	B	KP663745	B
KACNH3	2014	Republic of Korea	KP663743	B	KP663738	B	KP663739	B
KAGBH5	2014	Republic of Korea	KP663737	B	KP663741	B	KP663742	B
KAGBH6	2014	Republic of Korea	KP663740	B	KP663735	B	KP663736	B
KAGWH3	2014	Republic of Korea	KP663734	B	KP663732	D	KP663733	D
KASJH	2014	Republic of Korea	KP663731	D	MZ501602	B	MZ501603	B
U2014	2014	Republic of Korea	MZ501601	B	MG737085	D	MG920818	D
KACNH	2013	Republic of Korea	MG736976	D	MG737092	C	MG737200	B
KACNH2	2013	Republic of Korea	MG736983	B	MG737096	B	MG737204	B
KADGH2	2013	Republic of Korea	MG736987	B	MG737100	B	MG737208	B
KADGH3	2013	Republic of Korea	MG736991	B	MG737102	B	MG737210	B
KADGH5	2013	Republic of Korea	MG736993	B	MG737083	B	MG737192	B
KAGBH	2013	Republic of Korea	MG736974	B	MG737086	B	MG737194	B

Isolate	Year	Location	L		M		S	
			**Accession**	**Genotype**	**Accession**	**Genotype**	**Accession**	**Genotype**
KAGBH2	2013	Republic of Korea	MG736977	B	MG737088	B	MG737196	B
KAGBH3	2013	Republic of Korea	MG736979	B	MG737099	B	MG737207	B
KAGBH4	2013	Republic of Korea	MG736990	B	MG737087	B	MG737195	B
KAGNH2	2013	Republic of Korea	MG736978	B	MG737098	B	MG737206	B
KAGNH3	2013	Republic of Korea	MG736989	B	MG737084	B	MG737193	B
KAGWH	2013	Republic of Korea	MG736975	B	MG737095	A	MG737203	A
KAGWH2	2013	Republic of Korea	MG736986	A	MG737090	A	MG737198	A
KAICH	2013	Republic of Korea	MG736981	A	MG737082	B	PQ463065	D
KAJJH	2013	Republic of Korea	MG736973	B	PQ463040	D	HQ141609	C
LLL	2023	Shandong	PQ463015	D	HQ141608	C	MT114260	C
LN2/China/2010	2010	Liaoning	HQ141607	C	MT114216	C	MT114261	D
LN2010-001	2010	Liaoning	MT114200	C	MT114217	D	MT114262	C
LN2010-017	2010	Liaoning	MT114201	D	MT114218	C	MT114263	D
LN2011-001	2011	Liaoning	MT114202	C	MT114219	C	MT114265	D
LN2011-037	2011	Liaoning	MT114203	A	MT114221	C	MT114264	A
LN2011-pc	2011	Liaoning	MT114204	D	MT114220	A	MT114266	C
LN2011-xq	2011	Liaoning	MT114205	D	MT114222	C	MT114267	D
LN2012-030	2012	Liaoning	MT114206	C	MT114223	D	MT114268	D
LN2012-038	2012	Liaoning	MT114207	D	MT114224	C	MT114269	C
LN2012-047	2012	Liaoning	MT114208	D	MT114225	C	KF887431	D
LN2012-049	2012	Liaoning	MT114209	C	KF887436	C	KF887432	D
LN2012-14	2012	Liaoning	KF887441	D	KF887437	C	KF887433	D
LN2012-34	2012	Liaoning	KF887442	D	KF887438	C	KF887434	D
LN2012-41	2012	Liaoning	KF887443	D	KF887439	C	KF887435	C
LN2012-42	2012	Liaoning	KF887444	D	KF887440	C	MT114270	C
LN2012-58	2012	Liaoning	KF887445	D	MT114226	C	MT114271	D
LN2013-019	2013	Liaoning	MT114210	C	MT114227	C	MT114272	A
LN2013-029	2013	Liaoning	MT114211	C	MT114228	A	MT114273	D
LN2013-030	2013	Liaoning	MT114212	A	MT114229	C	MT114274	C
LN2013-035	2013	Liaoning	MT114213	D	MT114230	C	MT114275	C
LN2013-047	2013	Liaoning	MT114214	C	MT114231	C	HQ141612	A
LN2013-065	2013	Liaoning	MT114215	C	HQ141611	A	MT309105	D
LN3/China/2010	2010	Liaoning	HQ141610	A	MT309102	C	MT309106	D
LNDLChina2014-49	2014	Liaoning	MT309099	D	MT309103	D	MT309107	D
LNDLChina2014-50	2014	Liaoning	MT309100	D	MT309104	D	MT232960	A
LNDLChina2014-51	2014	Liaoning	MT309101	D	MT232961	A	PQ463087	D
LNHDG2019-6	2019	Liaoning	MT232962	A	PQ463062	D	PQ463083	D
LYH	2024	Shandong	PQ463037	D	PQ463058	D	MG737191	B

Isolate	Year	Location	L		M		S	
			Accession	Genotype	Accession	Genotype	Accession	Genotype
LYR	2024	Shandong	PQ463033	D	MG737091	B	PQ463068	D
KAJJH2	2013	Republic of Korea	MG736982	B	PQ463043	D	LC570787	B
MYA	2023	Shandong	PQ463018	D	LC570788	B	LC325239	B
ArtSp/2017	2017	Japan	LC325235	B	LC325237	B	LC325238	B
SkrP/2017	2017	Japan	LC325234	B	LC325236	B	LC590892	A
JaOF2017-7	2017	Japan	LC590890	A	LC590891	A	LC536536	B
A17	2016	Japan	LC536556	B	LC536546	B	LC536537	B
N2	2016	Japan	LC536557	B	LC536547	B	LC536538	B
N4	2016	Japan	LC536558	B	LC536548	B	MG737199	B
KAJNH	2013	Republic of Korea	MG736980	A	LC536550	B	LC536540	B
N10	2016	Japan	LC536560	B	MG737089	A	MG737197	A
KAJNH3	2013	Republic of Korea	MG736988	B	LC536551	B	LC536541	B
N12	2016	Japan	LC536561	B	MG737097	B	KR698325	B
NB31/CHN/2013	2013	Zhejiang	KR698351	B	LC536552	B	KR698326	B
NB32/CHN/2013	2013	Zhejiang	KR698352	B	KR698338	B	KR698327	A
NB34/CHN/2013	2013	Zhejiang	KR698353	A	KR698339	A	KR698328	B
NB38/CHN/2013	2013	Zhejiang	KR698354	A	KR698340	A	KR698329	B
NB39/CHN/2014	2014	Zhejiang	KR698355	B	KR698341	A	LC536542	B
N14	2016	Japan	LC536562	B	KR698342	B	LC536543	B
N19	2016	Japan	LC536563	B	LC536553	B	KR230808	A
20-China_Henan-61	2020	Henan	OM453529	D	OM452850	D	OM452151	D
PYL	2024	Shandong	PQ463022	D	PQ463047	D	PQ463072	D
QD7	2014	Shandong	KR706567	C	KR706566	C	KR706565	C
QingDao018	2016	Shandong	MF357031	D	MF357017	D	MF357024	D
JS2014-Hedgehog-01	2014	Jiangsu	KR230768	A	KR230788	A	KR698330	B
JS2014-Hedgehog-02	2014	Jiangsu	KR230769	C	KR698343	B	KR698331	B
Rodent01/NB/CHN/2014	2014	Zhejiang	KR698356	B	KR698344	B	KR230809	C
Rodent02/NB/CHN/2014	2014	Zhejiang	KR698357	B	KR230789	C	PP506387	E
SD2011-006	2011	Shandong	MT005200	E	MT005242	E	MT114290	E
SD2011-027	2011	Shandong	MT005201	D	MT005243	D	MT114291	D
SD2011-028	2011	Shandong	MT005202	A	MT005244	A	MT114292	A
SD2011-031	2011	Shandong	MT005203	D	MT005245	D	MT114293	D
SD2011-032	2011	Shandong	MT005204	D	MT005246	D	MT114294	D
SD2011-035	2011	Shandong	MT005205	D	MT005247	D	MT114295	D
SD2011-036	2011	Shandong	MT005206	D	MT005248	D	MT114296	D
SD2011-038	2011	Shandong	MT005207	D	MT005249	D	MT114297	D
SD2011-039	2011	Shandong	MT005208	D	MT005250	D	MT114298	D
SD2011-040	2011	Shandong	MT005209	E	MT005251	E	MT114299	E

Isolate	Year	Location	L		M		S	
			Accession	Genotype	Accession	Genotype	Accession	Genotype
SD2011-049	2011	Shandong	MT005210	D	MT005252	D	MT114300	D
SD2011-054	2011	Shandong	MT005211	D	MT005253	D	MT114301	D
SD2011-057	2011	Shandong	MT005212	D	MT005254	D	MT114302	D
SD2012-017	2012	Shandong	MT005213	E	MT005255	E	MT114303	E
SD2012-050	2012	Shandong	MT005214	D	MT005256	D	MT114304	D
SD2012-068	2012	Shandong	MT005215	D	MT005257	D	MT114305	D
SD2012-076	2012	Shandong	MT005216	D	MT005258	D	MT114306	D
SD2012-081	2012	Shandong	MT005217	A	MT005259	A	MT114307	A
SD2013-054	2013	Shandong	MT005218	E	MT005260	E	MT114308	E
SD2013-055	2013	Shandong	MT005219	D	MT005261	A	MT114309	A
SD2013-056	2013	Shandong	MT005220	A	MT005262	A	MT114310	A
SD2013-058	2013	Shandong	MT005221	D	MT005263	D	MT114311	D
SD2013-073	2013	Shandong	MT005222	D	MT005264	D	MT114312	D
SD2013-076	2013	Shandong	MT005223	A	MT005265	A	MT114313	A
SD2013-077	2013	Shandong	MT005224	D	MT005266	D	MT114314	D
SD2013-080	2013	Shandong	MT005225	D	MT005267	D	MT114315	D
SD2013-081	2013	Shandong	MT005226	D	MT005268	E	MT114316	D
SD2013-090	2013	Shandong	MT005227	D	MT005269	D	MT114317	E
SD2013-094	2013	Shandong	MT005228	D	MT005270	D	MT114318	D
SD2014-059	2014	Shandong	MT005229	D	MT005271	D	MT114319	D
SD2014-067	2014	Shandong	MT005230	E	MT005272	E	MT114320	E
SD2014-146	2014	Shandong	MT005231	D	MT005273	D	MT114321	D
SD2014-148	2014	Shandong	MT005232	D	MT005274	D	MT114322	D
SD2014-157	2014	Shandong	MT005233	E	MT005275	E	MT114323	E
SD2014-163	2014	Shandong	MT005234	D	MT005276	D	MT114324	D
SD2014-198	2014	Shandong	MT005235	D	MT005277	D	MT114325	D
SD2014-209	2014	Shandong	MT005236	D	MT005278	D	MT114326	D
SD2015-064	2015	Shandong	MT005237	D	MT005279	D	MT114327	D
SD2015-086	2015	Shandong	MT005238	D	MT005280	D	MT114328	D
SD2015-088	2015	Shandong	MT005239	D	MT005281	D	MT114329	D
SD2015-089	2015	Shandong	MT005240	D	MT005282	D	MT114330	D
SD2015-101	2015	Shandong	MT005241	D	MT005283	D	MT114331	D
SD24/China/2010	2010	Shandong	HM802200	D	HM802201	D	HM802205	D
SD4/China/2010	2010	Shandong	HM802202	E	HM802203	E	HM802204	E
SDF	2024	Shandong	PQ463023	D	PQ463048	D	PQ463073	D
SDLZtick12/2010	2010	Shandong	JQ684871	C	JQ684872	C	JQ684873	C
SDTA_1	2015	Shandong	KX641909	D	KX641913	D	KX641917	D
SDTA_2	2015	Shandong	KX641910	D	KX641914	D	KX641918	D

Isolate	Year	Location	L		M		S	
			Accession	Genotype	Accession	Genotype	Accession	Genotype
SDTA_3	2015	Shandong	KX641911	D	KX641915	D	KX641919	D
SDTA_4	2015	Shandong	KX641912	D	KX641916	D	KX641920	D
SDtickSFTS2018-01	2017	Shandong	MT413432	D	MT413433	D	MT413434	D
SDYY104/China/2011	2011	Shandong	KU239114	D	KU239112	D	KU239113	D
SF18-13	2018	Zhejiang	OQ938861	A	OQ938887	A	OQ938882	A
SF18-15	2018	Zhejiang	OQ938862	A	OQ938888	A	OQ938883	A
SF21-6	2021	Zhejiang	OQ938863	A	OQ938889	A	OQ938884	A
SF22-11	2019	Zhejiang	OQ938865	A	OQ938891	A	OQ938886	A
SF22-7	2021	Zhejiang	OQ938864	A	OQ938890	A	OQ938885	A
SF23-3	2023	Zhejiang	OQ938866	A	OQ938872	A	OQ938880	A
SF23-5	2023	Zhejiang	OQ938868	A	OQ938873	A	OQ938879	A
SF23-6	2023	Zhejiang	OQ938867	A	OQ938874	A	OQ938881	A
N21	2016	Japan	LC536564	B	LC536554	B	LC536544	B
WH23-fox1	2023	Shandong	PP506403	E	PP506395	E	PP506388	E
N7	2016	Japan	LC536559	B	LC536549	B	LC536539	B
SFTSV/LTBMH3	2019	Hubei	MZ965009	D	MZ965011	D	MZ965013	D
SFTSV/LTSL1	2019	Hubei	MZ965010	D	MZ965012	D	MZ965014	D
SFTSV_JX2013	2014	Jiangxi	KY848825	D	KY848826	D	KY848827	D
WH23-fox2	2023	Shandong	PP506404	E	PP506396	E	MG737205	B
KASUH	2013	Republic of Korea	MG736992	B	MG737101	B	PP066885	D
KAUSH	2013	Republic of Korea	MG736984	B	MG737093	B	MG737209	B
SRH186	2014	Japan	LC579714	B	LC579713	B	LC579712	B
SRH187	2014	Japan	LC579711	B	LC579710	B	LC579709	B
TNB1580	2013	Japan	LC579717	A	LC579716	A	LC579715	A
TNB1590	2013	Japan	LC579720	A	LC579719	A	LC579718	A
SPL128A	2014	Japan	AB983532	B	AB985325	B	AB985558	B
SPL129A	2014	Japan	AB983533	B	AB985326	B	AB985559	B
SPL054A	2013	Japan	AB983498	B	AB985293	B	AB985524	B
SPL055A	2013	Japan	AB983499	B	AB985294	B	AB985525	B
SPL057A	2013	Japan	AB983500	B	AB985295	B	AB985526	B
SPL060A	2013	Japan	AB983501	B	AB985296	B	AB985527	B
SPL062A	2013	Japan	AB983502	B	AB985297	B	AB985528	B
SPL066A	2013	Japan	AB983503	B	AB985298	B	AB985529	B
SPL067A	2013	Japan	AB983504	B	AB985299	B	AB985530	B
SPL069A	2013	Japan	AB983505	B	AB985300	B	AB985531	B
SPL070A	2013	Japan	AB983506	B	AB985301	B	AB985532	B
SPL071A	2013	Japan	AB983507	B	AB985302	B	AB985533	B
SPL075A	2013	Japan	AB983509	B	AB985303	B	AB985535	B

Isolate	Year	Location	L		M		S	
			Accession	Genotype	Accession	Genotype	Accession	Genotype
SPL077A	2013	Japan	AB983510	B	AB985304	B	AB985536	B
SPL078A	2013	Japan	AB983511	B	AB985305	B	AB985537	B
SPL082A	2013	Japan	AB983512	B	AB985306	B	AB985538	B
SPL084A	2013	Japan	AB983513	B	AB985307	B	AB985539	B
SPL086A	2013	Japan	AB983514	B	AB985308	B	AB985540	B
SPL089A	2013	Japan	AB983516	B	AB985310	B	AB985542	B
SPL090A	2013	Japan	AB983517	B	AB985311	B	AB985543	B
SPL097A	2013	Japan	AB983518	B	AB985312	B	AB985544	B
SPL100A	2013	Japan	AB983519	B	AB985313	B	AB985545	B
SPL104A	2013	Japan	AB983520	B	AB985314	B	AB985546	B
SPL105A	2013	Japan	AB983521	B	AB985315	B	AB985547	B
SPL106A	2013	Japan	AB983522	B	AB985316	B	AB985548	B
SPL107A	2013	Japan	AB983523	B	AB985317	B	AB985549	B
SPL108A	2013	Japan	AB983524	B	AB985318	B	AB985550	B
SPL112A	2013	Japan	AB983525	B	AB985319	B	AB985551	B
SPL114A	2013	Japan	AB983526	B	AB985320	B	AB985552	B
SPL117A	2013	Japan	AB983527	B	AB985321	B	AB985553	B
SPL121A	2013	Japan	AB983529	B	AB985323	B	AB985555	B
SPL124A	2013	Japan	AB983530	B	AB985324	B	AB985556	B
SPL003A	2012	Japan	AB817980	B	AB817988	B	AB817996	B
SPL004A	2012	Japan	AB817981	B	AB817989	B	AB817997	B
SPL005A	2012	Japan	AB817982	B	AB817990	B	AB817998	B
SPL035A	2012	Japan	AB817986	B	AB817994	B	AB818002	B
SPL053A	2012	Japan	AB983497	B	AB985292	B	AB985523	B
SD01	2022	Shandong	PP066883	D	PP066884	D	OQ160819	C
HB801	2020	Hubei	OQ160821	C	OQ160820	C	OM452152	C
20-China_Henan-65	2020	Henan	OM453530	C	AB985322	B	OM452153	C
20-China_Henan-67	2020	Henan	OM453531	C	OM452851	C	OM452154	C
20-China_Henan-73	2020	Henan	OM453532	C	OM452852	C	PQ463064	D
SYS	2023	Shandong	PQ463014	D	OM452853	C	PQ374006	B
SZ-202101	2021	Guangdong	PQ374004	B	PQ463039	D	PQ463074	D
SZH	2024	Shandong	PQ463024	D	PQ374005	A	MF357025	D
TaiAn036	2016	Shandong	MF357032	D	PQ463049	D	MF357026	D
TaiAn038	2016	Shandong	MF357033	D	MF357018	D	MG737201	B
KAUSH2	2013	Republic of Korea	MG736985	B	MF357019	D	AB985554	B
SPL120A	2012	Japan	AB983528	B	MG737094	B	AB817995	B
YG1	2012	Japan	AB817979	B	AB817987	B	MN830173	B
TP1910a	2019	Taiwan	MN910270	B	AB817992	B	MG737202	B

Isolate	Year	Location	L		M		S	
			Accession	Genotype	Accession	Genotype	Accession	Genotype
Gangwon/Korea/2012	2012	Republic of Korea	KF358691	C	MN830174	B	OM452155	D
20-China_Henan-75	2020	Henan	OM453533	D	KF358692	C	AB818000	B
SPL030A	2010	Japan	AB817984	B	OM452854	D	AB817999	B
SPL010A	2005	Japan	AB817983	B	AB817991	B	OM452156	C
20-China_Henan-77	2020	Henan	OM453534	C	OM452855	C	PQ671012	C
G3	2023	Anhui	PQ671014	C	PQ671013	C	OM452157	C
20-China_Henan-78	2020	Henan	OM453535	C	OM452856	C	OM452158	A
20-China_Henan-81	2020	Henan	OM453536	A	OM452857	A	KF358693	C
WH23-P1	2023	Shandong	PP506401	E	PP506393	E	PP506385	E
WH23-P2	2023	Shandong	PP506402	E	PP506394	E	PP506386	E
WJY	2024	Shandong	PQ463025	D	PQ463050	D	PQ463075	D
WSP	2024	Shandong	PQ463035	D	PQ463060	D	PQ463085	D
WXQ	2024	Shandong	PQ463026	D	PQ463051	D	PQ463076	D
WXY	2024	Shandong	PQ463031	D	PQ463056	D	PQ463081	D
XNQ	2023	Shandong	PQ463017	D	PQ463042	D	PQ463067	D
WH23-E1	2023	Shandong	PP506405	E	PP506397	E	PP506389	E
WH23-E2	2023	Shandong	PP506406	E	PP506398	E	PP506390	E
WH23-E3	2023	Shandong	PP506407	D	PP506399	D	PP506391	D
WH23-E5	2023	Shandong	PP506408	E	PP506400	E	PP506392	E
20-China_Henan-86	2020	Henan	OM453537	C	OM452858	C	OM452159	C
SPL032A	2005	Japan	AB817985	B	AB817993	B	AB818001	B
20-China_Henan-88	2020	Henan	OM453538	C	OM452859	C	OM452160	C
20-China_Henan-90	2020	Henan	OM453539	C	OM452860	C	OM452161	C
20-China_Henan-93	2020	Henan	OM453541	C	OM452862	C	OM452163	C
20-China_Henan-99	2020	Henan	OM453542	A	OM452863	A	OM452164	A
20-China_HNPY-001	2020	Henan	OQ413509	A	OQ413510	A	OQ413511	A
22-China_Henan-13	2022	Henan	ON402247	C	ON402253	C	ON402259	C
22-China_Henan-14	2022	Henan	ON402248	C	ON402254	C	ON402260	C
22-China_Henan-16	2022	Henan	ON402249	C	ON402255	C	ON402261	C
22-China_Henan-18	2022	Henan	ON402250	C	ON402256	C	ON402262	C
22-China_Henan-20	2022	Henan	ON402251	C	ON402257	C	ON402263	C
YSQ	2024	Shandong	PQ463027	D	PQ463052	D	PQ463077	D
22-China_Henan-326	2022	Henan	ON402252	C	ON402258	C	ON402264	C
YSHX002	NA	Henan	KF356551	D	KF356540	D	KF356528	D
ZCL	2023	Shandong	PQ463013	D	PQ463038	D	PQ463063	D
ZFQ	2024	Shandong	PQ463032	D	PQ463057	D	PQ463082	D
Zhejiang/01/2011	2011	Zhejiang	KJ597825	B	KJ597824	B	KJ597823	B
Zhejiang/01/2021	2021	Zhejiang	OQ658514	E	OQ658515	E	OQ658516	E

Isolate	Year	Location	L		M		S	
			Accession	Genotype	Accession	Genotype	Accession	Genotype
ZHX	2024	Shandong	PQ463030	D	PQ463055	D	PQ463080	D
ZHY	2024	Shandong	PQ463028	D	PQ463053	D	PQ463078	D
ZJ2012-03	2012	Zhejiang	KY273264	B	KY273268	B	KY273272	B
ZJ2013-06	2013	Zhejiang	KP280206	B	KP280204	B	KP280205	B
ZJ2013-07	2013	Zhejiang	KY273265	A	KY273269	A	KY273273	A
ZJ2014-01	2014	Zhejiang	KY273266	B	KY273270	B	KY273274	B
ZJ2014-02	2014	Zhejiang	KY273267	A	KY273271	A	KY273275	A
ZJ2018-01	2018	Zhejiang	MZ561692	A	MZ561691	A	MZ561690	A
ZJDH-SCH/05/2016	2015	Zhejiang	PV815698	A	PV815694	A	PV815690	A
ZJDH-YJH/06/2016	2016	Zhejiang	PV815696	A	PV815692	A	PV815688	A
ZJDH-YSQ/06/2016	2016	Zhejiang	PV815697	A	PV815693	A	PV815689	A
ZJDH-YWX/05/2017	2017	Zhejiang	PV815699	A	PV815695	A	PV815691	A
ZJnb001_2016	2016	Zhejiang	PV767530	B	PV767874	B	PV774073	B
ZJnb002_2017	2017	Zhejiang	PV767531	A	PV767875	A	PV774074	A
ZJnb003_2017	2017	Zhejiang	PV767532	B	PV767876	B	PV774075	B
ZJnb004_2017	2017	Zhejiang	PV767790	B	PV768134	B	PV774333	B
ZJnb005_2018	2018	Zhejiang	PV767791	B	PV768135	B	PV774334	B
ZJnb006_2018	2018	Zhejiang	PV767792	A	PV768136	A	PV774335	A
ZJnb007_2013	2013	Zhejiang	PV767793	A	PV768137	A	PV774336	A
ZJnb008_2013	2013	Zhejiang	PV767794	B	PV768138	B	PV774337	B
ZJnb009_2013	2013	Zhejiang	PV767795	B	PV768139	B	PV774338	B
ZJnb010_2013	2013	Zhejiang	PV767796	B	PV768140	B	PV774339	B
ZJnb011_2013	2013	Zhejiang	PV767797	A	PV768141	A	PV774340	A
ZJnb012_2013	2013	Zhejiang	PV767798	B	PV768142	B	PV774341	B
ZJnb013_2013	2013	Zhejiang	PV767799	A	PV768143	A	PV774342	A
ZJnb014_2014	2014	Zhejiang	PV767800	B	PV768144	B	PV774343	B
ZJnb015_2014	2014	Zhejiang	PV767801	A	PV768145	A	PV774344	A
ZJnb016_2014	2014	Zhejiang	PV767802	A	PV768146	A	PV774345	A
ZJnb017_2014	2014	Zhejiang	PV767803	B	PV768147	B	PV774346	B
ZJnb018_2014	2014	Zhejiang	PV767804	A	PV768148	A	PV774347	A
ZJnb019_2014	2014	Zhejiang	PV767805	B	PV768149	B	PV774348	B
ZJnb020_2014	2014	Zhejiang	PV767806	B	PV768150	B	PV774349	B
ZJnb021_2014	2014	Zhejiang	PV767807	B	PV768151	B	PV774350	B
ZJnb022_2014	2014	Zhejiang	PV767808	B	PV768152	B	PV774351	B
ZJnb023_2015	2015	Zhejiang	PV767809	A	PV768153	A	PV774352	A
ZJnb024_2015	2015	Zhejiang	PV767810	A	PV768154	A	PV774353	A
ZJnb025_2018	2018	Zhejiang	PV767811	E	PV768155	A	PV774354	E
ZJZHSH-AM/China/05/2012	2012	Zhejiang	KR017827	B	KR017846	B	KR017808	B

Isolate	Year	Location	L		M		S	
			Accession	Genotype	Accession	Genotype	Accession	Genotype
ZJZHSH-CXD/China/05/2012	2012	Zhejiang	KR017839	A	KR017858	A	KR017820	A
ZJZHSH-FDE/China/06/2012	2012	Zhejiang	KR017840	B	KR017859	B	KR017821	B
ZJZHSH-FZHR/China/07/2013	2013	Zhejiang	KR017843	A	KR017862	A	KR017824	A
ZJZHSH-HCY/China/06/2012	2012	Zhejiang	KR017836	B	KR017855	B	KR017817	B
ZJZHSH-HYC/China/05/2012	2012	Zhejiang	KR017828	B	KR017847	B	KR017809	B
ZJZHSH-HYJ/China/07/2012	2012	Zhejiang	KR017833	B	KR017852	B	KR017814	B
ZJZHSH-LHZH/China/06/2012	2012	Zhejiang	KR017838	B	KR017857	B	KR017819	B
ZJZHSH-LU/China/10/2011	2011	Zhejiang	KR017831	B	KR017850	B	KR017812	B
ZJZHSH-LWL/China/08/2014	2014	Zhejiang	KR017844	B	KR017863	B	KR017825	B
ZJZHSH-LWLTick/China/07/2014	2014	Zhejiang	MT236315	B	MT236316	B	MT236317	B
ZJZHSH-SHZ/China/05/2012	2012	Zhejiang	KR017829	B	KR017848	B	KR017810	B
ZJZHSH-WRF/China/08/2014	2014	Zhejiang	KR017845	B	KR017864	B	KR017826	B
ZJZHSH-WSH/China/08/2012	2012	Zhejiang	KR017835	A	KR017854	A	KR017816	A
ZJZHSH-XAM/China/06/2012	2012	Zhejiang	KR017834	B	KR017853	B	KR017815	B
ZJZHSH-YJX/China/06/2012	2012	Zhejiang	KR017841	B	KR017860	B	KR017822	B
ZJZHSH-ZLD/China/06/2012	2012	Zhejiang	KR017832	B	KR017851	B	KR017813	B
ZJZHSH-ZLN/China/06/2012	2012	Zhejiang	KR017837	B	KR017856	B	KR017818	B
ZJzs001_2016	2016	Zhejiang	PV767812	B	PV768156	B	PV774355	B
ZJzs002_2016	2016	Zhejiang	PV767813	A	PV768157	A	PV774356	A
ZJzs003_2016	2016	Zhejiang	PV767814	A	PV768158	A	PV774357	A
ZJzs004_2016	2016	Zhejiang	PV767815	B	PV768159	B	PV774358	B
ZJzs005_2016	2016	Zhejiang	PV767816	B	PV768160	B	PV774359	B
ZJzs006_2016	2016	Zhejiang	PV767817	B	PV768161	B	PV774360	B
ZJzs007_2016	2016	Zhejiang	PV767818	B	PV768162	B	PV774361	B
ZJzs008_2016	2016	Zhejiang	PV767819	B	PV768163	B	PV774362	B
ZJzs009_2017	2017	Zhejiang	PV767820	B	PV768164	B	PV774363	B
ZJzs011_2016	2016	Zhejiang	PV767821	B	PV768165	B	PV774364	B
ZJzs012_2016	2016	Zhejiang	PV767822	B	PV768166	B	PV774365	B
ZJzs014_2016	2016	Zhejiang	PV767823	B	PV768167	B	PV774366	B
ZJzs015_2016	2016	Zhejiang	PV767824	B	PV768168	B	PV774367	B
ZJzs016_2016	2016	Zhejiang	PV767825	B	PV768169	B	PV774368	B
ZJzs017_2016	2016	Zhejiang	PV767826	A	PV768170	A	PV774369	A
ZJzs018_2016	2016	Zhejiang	PV767827	A	PV768171	A	PV774370	A
ZJzs019_2015	2015	Zhejiang	PV767828	B	PV768172	B	PV774371	B
ZJzs020_2016	2016	Zhejiang	PV767829	B	PV768173	B	PV774372	B
ZJzs021_2015	2015	Zhejiang	PV767830	B	PV768174	B	PV774373	B
ZJzs022_2015	2015	Zhejiang	PV767831	D	PV768175	D	PV774374	D
ZJzs023_2015	2015	Zhejiang	PV767832	B	PV768176	B	PV774375	B

Isolate	Year	Location	L		M		S	
			Accession	Genotype	Accession	Genotype	Accession	Genotype
ZJzs024_2015	2015	Zhejiang	PV767833	A	PV768177	A	PV774376	A
ZJzs025_2015	2015	Zhejiang	PV767834	B	PV768178	B	PV774377	B
ZJzs026_2015	2015	Zhejiang	PV767835	B	PV768179	B	PV774378	B
ZJzs027_2013	2013	Zhejiang	PV767836	B	PV768180	B	PV774379	B
ZJzs028_2012	2012	Zhejiang	PV767837	B	PV768181	B	PV774380	B
ZJzs029_2012	2012	Zhejiang	PV767838	B	PV768182	B	PV774381	B
ZJzs030_2014	2014	Zhejiang	PV767839	B	PV768183	B	PV774382	B
ZJzs033_2014	2014	Zhejiang	PV767840	B	PV768184	A	PV774383	A
ZJzs034_2014	2014	Zhejiang	PV767841	A	PV768185	A	PV774384	A
ZJzs035_2013	2013	Zhejiang	PV767842	B	PV768186	B	PV774385	B
ZJzs036_2013	2013	Zhejiang	PV767843	B	PV768187	B	PV774386	B
ZJzs037_2013	2013	Zhejiang	PV767844	B	PV768188	B	PV774387	B
ZJzs038_2015	2015	Zhejiang	PV767845	B	PV768189	B	PV774388	B
ZJzs039_2013	2013	Zhejiang	PV767846	B	PV768190	B	PV774389	B
ZJzs040_2013	2013	Zhejiang	PV767847	B	PV768191	B	PV774390	B
ZJzs041_2013	2013	Zhejiang	PV767848	B	PV768192	B	PV774391	B
ZJzs042_2013	2013	Zhejiang	PV767849	B	PV768193	B	PV774392	B
ZJzs043_2013	2013	Zhejiang	PV767850	B	PV768194	B	PV774393	B
ZJzs044_2013	2013	Zhejiang	PV767851	A	PV768195	A	PV774394	A
ZJzs045_2013	2013	Zhejiang	PV767852	B	PV768196	B	PV774395	B
ZJzs046_2013	2013	Zhejiang	PV767853	B	PV768197	B	PV774396	B
ZJzs047_2015	2015	Zhejiang	PV767854	B	PV768198	B	PV774397	B
ZJzs048_2015	2015	Zhejiang	PV767855	A	PV768199	A	PV774398	A
ZJzs049_2015	2015	Zhejiang	PV767856	B	PV768200	B	PV774399	B
ZJzs050_2015	2015	Zhejiang	PV767857	B	PV768201	B	PV774400	B
ZJzs051_2015	2015	Zhejiang	PV767858	B	PV768202	B	PV774401	B
ZJzs052_2015	2015	Zhejiang	PV767859	B	PV768203	B	PV774402	B
ZJzs053_2018	2018	Zhejiang	PV767860	B	PV768204	B	PV774403	B
ZJzs054_2016	2016	Zhejiang	PV767861	B	PV768205	B	PV774404	B
ZJzs055_2017	2017	Zhejiang	PV767862	A	PV768206	A	PV774405	A
ZJzs056_2017	2017	Zhejiang	PV767863	B	PV768207	B	PV774406	B
ZJzs057_2017	2017	Zhejiang	PV767864	D	PV768208	D	PV774407	D
ZJzs058_2017	2017	Zhejiang	PV767865	D	PV768209	D	PV774408	D
ZJzs059_2015	2015	Zhejiang	PV767866	B	PV768210	B	PV774409	B
ZKL	2024	Shandong	PQ463034	D	PQ463059	D	PQ463084	D
ZWY	2023	Shandong	PQ463016	D	PQ463041	D	PQ463066	D
ZYL	2024	Shandong	PQ463029	D	PQ463054	D	PQ463079	D

NA, not applicable.

Table S5. Summary of viral reassortment events.

Isolate	L genotype	M genotype	S genotype	Year	Location
YSC3	C	C	A	2011	Henan
2011YSC60	A	C	A	2011	Henan
AHL/China/2011	A	A	D	2011	Anhui
HB2011-071	D	C	C	2011	Hubei
HB2011-137	C	C	D	2011	Hubei
LN2011-037	A	C	D	2011	Liaoning
LN2011-pc	D	C	D	2011	Liaoning
LN2011-xq	D	A	A	2011	Liaoning
2011YPQ12	C	A	C	2012	Henan
HB2012-197	B	A	B	2012	Hubei
LN2012-047	D	C	D	2012	Liaoning
LN2012-14	D	C	D	2012	Liaoning
LN2012-34	D	C	D	2012	Liaoning
LN2012-41	D	C	D	2012	Liaoning
LN2012-42	D	C	D	2012	Liaoning
LN2012-58	D	C	C	2012	Liaoning
13-China_Henan-100	C	D	D	2013	Henan
LN2013-029	C	C	D	2013	Liaoning
LN2013-035	D	C	D	2013	Liaoning
SD2013-055	D	A	A	2013	Shandong
SD2013-081	D	E	D	2013	Shandong
SD2013-090	D	D	E	2013	Shandong
NB32/CHN/2013	B	A	B	2013	Zhejiang
NB38/CHN/2013	A	A	B	2013	Zhejiang
KACNH2	B	C	B	2013	Republic of Korea
14-China_Henan-438	C	A	A	2014	Henan
HB2014-22	C	C	D	2014	Hubei
JS2014-39	C	C	A	2014	Jiangsu
LNDLChina2014-49	D	C	D	2014	Liaoning
ZJzs033_2014	B	A	A	2014	Zhejiang
15-China_Henan-15	D	C	C	2015	Henan
15-China_Henan-16	C	D	D	2015	Henan
15-China_Henan-356	D	C	C	2015	Henan
15-China_Hubei-234	C	D	D	2015	Henan
DanDong273	D	B	D	2016	Liaoning
HB2016-013	D	D	C	2016	Hubei
HB2016-034	C	C	D	2016	Hubei
16KS55	D	B	D	2016	Republic of Korea
16KS45	B	A	A	2016	Republic of Korea
HB2017-02	D	B	D	2017	Hubei
HBHG38	A	A	D	2017	Hubei
HBSZ55	C	C	D	2017	Hubei
HZ2018-23	B	C	C	2018	Zhejiang
ZJnb025_2018	E	A	E	2018	Zhejiang
AH-2019-ZXC	D	C	D	2019	Anhui
20-China_Henan-111	D	C	C	2020	Henan
20-China_Henan-133	A	C	C	2020	Henan
20-China_Henan-180	C	A	A	2020	Henan
20-China_Henan-190	C	A	A	2020	Henan
20-China_Henan-24	A	C	C	2020	Henan

Isolate	L genotype	M genotype	S genotype	Year	Location
20-China_Hubei-191	C	D	D	2020	Hubei
20-China_Hubei-233	C	A	C	2020	Hubei
20-China_Jiangxi-104	A	C	C	2020	Jiangxi
SZ-202101	B	A	B	2021	Guangdong
HBHG2021-04	C	C	D	2021	Hubei
HBHG2021-13	E	E	B	2021	Hubei
HBHG2021-W3	B	B	E	2021	Hubei
BNY21002	B	A	B	2021	Zhejiang
2022HNYGS10	C	C	A	2022	Henan
2022HNYSH7	A	A	D	2022	Henan
2022HNYSH12	C	C	D	2022	Henan
2022HNYLS11	D	D	C	2022	Henan
AHZ-2022-HBY	B	A	B	2022	Anhui
HBHG2022-36	B	D	D	2022	Hubei
HBSZ2022-11	D	C	C	2022	Hubei
HBSZ2022-54	D	C	D	2022	Hubei
HZ2022-2	B	A	B	2022	Zhejiang
HBSY2023-1	A	C	A	2023	Hubei
JX23WNZ	B	A	B	2023	Jiangxi
AH-2019-SDR	C	D	D	2023	Anhui
AHZ-2023-XYA	A	A	D	2023	Anhui
JX23WN	B	A	B	2023	Jiangxi
HZ2023-15	D	C	D	2023	Zhejiang
HZ2023-32	D	C	D	2023	Zhejiang

The table details 74 identified reassortment events based on the phylogenetic incongruence of the Large (L), Medium (M), and Small (S) segments. Genotypes (A–E) are assigned to each segment to illustrate the diverse genomic constellations

Table S6. Putative viral recombination events.

Segment	Recombinant Sequence	Minor Parental Sequence	Major Parental Sequence	Genotype	Location	Year	RDP	GENECONV	Bootscan	Maxchi	Chimaera	SiSscan	3Seq
L	KC292329	OM453334	OM453610	A	Henan	2012	+	+	+	+	+	+	+
	MT114235	MT005238	PP239065	C	Hubei	2012	+	+	+	+	+	+	+
	MT320802	MN509856	MN509896	C	Hubei	2017	+	+	+	+	–	+	+
	KC292349	MN509967	OM453550	A	Henan	2012	+	+	+	+	+	+	+
	KF711880	OM453027	OM453579	C	Henan	2011	+	+	+	+	+	+	+
	KF711863	OM453562	OM453009	A	Henan	2011	+	+	+	+	+	+	+
	OM452957	PQ463023	OP652093	D	Henan	2012	+	–	+	+	–	+	+
	KF711889	KF711884	OM453577	D	Henan	2011	–	–	+	+	–	+	+
	OM453009	MN509877	OM453151	A	Henan	2013	+	+	+	+	+	+	+
	OR709732	OR709734	OQ388743	B	Anhui	2023	+	+	+	+	+	+	+
	PP706874	PP706861	PX306839	B	Zhejiang	2023	+	+	–	+	+	+	+
	OM453266	OQ388770	MN509974	D	Henan	2016	+	+	+	+	+	+	+
	MF094739	MG737038	MG737073	B	Republic of Korea	2016	+	+	+	+	+	+	+
	PP706873	PP706861	PP706877	A	Zhejiang	2023	+	+	+	+	+	+	+
	KC292348	OM453483	KC292349	A	Henan	2012	–	+	+	+	+	+	+
	OQ388771	KY965119	OM453514	D	Hubei	2022	+	+	+	+	+	+	+
	OM453596	OM453080	OM453539	C	Henan	2019	+	+	+	+	+	+	+
	OM452994	OM453203	MN509896	D	Henan	2013	+	+	+	+	+	+	+
	MF094728	MG737071	OR797605	B	Republic of Korea	2016	+	+	+	+	+	+	+
	OM453018	OM453549	MT005238	D	Henan	2013	+	+	+	+	+	+	+
	OQ388770	OM453434	OP652107	C	Hubei	2022	+	+	+	+	+	+	–
	KR698352	PV767795	OQ938861	B	Zhejiang	2013	+	+	+	+	+	+	+
	OM453577	OM452955	OM453609	C	Henan	2019	+	+	+	–	–	+	+
	PP706875	LC590890	PX306839	B	Zhejiang	2023	+	+	+	+	+	+	+
	AB983501	AB983519	LC549339	B	Japan	2013	+	+	+	–	–	–	+
	PV767835	PV815699	MF094735	B	Zhejiang	2015	+	+	+	–	–	+	+
	OR709732	OR709734	OQ388743	B	Anhui	2023	+	+	+	–	–	+	+
	PP706880	KR230759	MT309101	D	Zhejiang	2023	+	+	+	+	+	+	+
	OM453602	PP239063	MN510016	C	Henan	2019	+	+	+	–	–	–	+
	MT005206	OQ658514	MT005238	D	Shandong	2011	+	+	+	–	–	+	+
	PV767825	MG737077	PV767819	B	Zhejiang	2016	+	+	+	–	–	–	+
	OM453119	OM453602	MT005223	A	Henan	2014	+	+	+	–	–	–	+
	OM453270	MN509932	OM453119	A	Henan	2016	+	+	+	+	+	+	+
	MT005226	PP706870	OM453367	D	Shandong	2013	+	+	–	+	+	+	+
	KR698352	KP280206	MZ773015	B	Zhejiang	2013	+	+	+	+	+	+	+
	PP706846	PP706834	MT114242	B	Zhejiang	2022	+	+	+	+	+	+	+
	PP239064	PP239063	KY933703	C	Hubei	2023	+	+	+	+	+	+	+
	PP706834	PX306839	OM453566	B	Zhejiang	2018	+	+	–	+	+	+	–

Segment	Recombinant Sequence	Minor Parental Sequence	Major Parental Sequence	Genotype	Location	Year	RDP	GENECONV	Bootscan	Maxchi	Chimaera	SiSscan	3Seq
	OR709730	OQ388775	OR709728	C	Anhui	2023	+	–	+	+	+	+	–
	KR698352	AB983500	OQ938866	B	Zhejiang	2013	+	+	+	+	–	+	+
	OM453591	KC292329	MN509881	C	Henan	2019	+	+	+	–	–	–	+
	KR017835	PV767835	PV767851	A	Zhejiang	2012	+	+	+	–	–	–	+
	MF094740	MG737073	MF094741	B	Republic of Korea	2016	+	+	+	–	–	+	+
	OM453579	OM453119	MN509971	C	Hubei	2019	+	+	+	+	–	–	+
	PP488495	PP488493	KY362305	A	Zhejiang	2023	+	+	+	–	–	–	+
	PV767848	OR762212	PV767795	B	Zhejiang	2013	+	+	+	+	+	+	–
	PV767848	PV767854	KP280206	B	Zhejiang	2013	–	+	+	+	+	+	+
	OR709730	MT005205	OR709734	C	Anhui	2023	+	+	+	–	–	–	+
M	MG737155	MG737161	PP488484	B	Republic of Korea	2016	+	+	+	+	–	+	+
	PV768145	PV768139	PV768143	A	Zhejiang	2014	+	+	+	+	+	+	+
	KY362353	KY362348	KY362345	A	Anhui	2014	+	+	+	+	+	+	+
	PV768201	KR017853	PV768138	B	Zhejiang	2015	–	+	+	+	+	+	+
	MG737120	MG737094	PP488484	D	Republic of Korea	2014	–	+	+	+	+	+	+
	MG737092	MN510063	PP656917	C	Republic of Korea	2013	–	+	+	+	–	+	+
	OQ388971	KY362359	OQ388998	B	Hubei	2022	+	+	+	+	+	+	+
	OQ388970	OR709725	MN510071	C	Hubei	2022	+	+	+	+	+	+	+
	MG737169	MG737168	MG737183	B	Republic of Korea	2016	–	+	+	+	–	+	+
	OM452391	OM452584	MN510159	A	Henan	2014	+	–	+	+	–	+	+
	OR709721	OR762208	OR682386	C	Anhui	2023	+	+	+	+	+	+	+
	OQ388989	KY965099	PQ463038	D	Hubei	2022	+	+	+	+	+	+	+
	KF711930	OM452630	OM452501	A	Henan	2012	–	–	–	+	+	+	+
	KR230776	OR762205	KY362354	C	Jiangsu	2013	+	+	+	+	+	+	+
	OP652075	OP652073	OM452259	D	Henan	2022	+	+	+	+	+	–	+
	KR017853	MG737089	PV768163	B	Zhejiang	2012	+	+	+	+	+	+	+
	OM452787	PP239067	KY789438	B	Henan	2020	+	+	–	+	+	+	+
	OQ388960	OQ388967	MN510084	A	Hubei	2020	+	+	+	+	+	+	+
	KY965103	MN510159	OP651049	B	Hubei	2016	+	+	+	+	+	–	+
	KR017854	PV768180	PV768171	A	Zhejiang	2012	+	+	+	+	+	–	+
	KF711945	OM452824	MN510139	C	Henan	2011	+	+	–	+	+	+	+
	KF356535	KF711927	OM452621	C	Henan	2011	+	+	+	–	–	+	+
	MF094759	MZ501602	PP488484	D	Republic of Korea	2016	+	+	+	–	–	+	+
	KR698339	PV768144	PV768184	A	Zhejiang	2013	+	+	+	–	–	+	+
	OM452259	OM452768	OM452864	D	Henan	2012	+	+	+	+	+	+	+
	KC292310	OM452857	OM452508	C	Henan	2012	+	+	+	+	–	–	+
	MT309104	MT309102	MF357014	D	Liaoning	2014	+	+	+	+	+	–	+
	OP652073	KY965093	MN510056	C	Henan	2022	+	+	+	–	–	–	+
	OM452332	HQ141596	MN510058	D	Henan	2013	–	+	+	+	+	+	+
	OM452307	OM452340	MH790413	C	Henan	2013	+	+	+	–	–	–	+
	KT890281	OR682392	KC505145	A	Jilin	2013	+	+	+	+	+	–	+

Segment	Recombinant Sequence	Minor Parental Sequence	Major Parental Sequence	Genotype	Location	Year	RDP	GENECONV	Bootscan	Maxchi	Chimaera	SiSscan	3Seq
	OP652071	KT890281	KF711946	C	Henan	2022	+	–	–	+	+	+	–
	OP652071	OM452584	KF711946	C	Henan	2022	+	+	+	–	–	–	+
S	OQ389106	OM451829	MT559337	C	Hubei	2022	–	+	+	+	+	+	+
	PV774373	PV774368	KR017816	B	Zhejiang	2015	+	+	+	+	+	+	+
	MK513931	MK513910	PV774408	D	Hubei	2015	+	+	+	+	+	–	+
	PV774369	MG737252	PV774347	A	Zhejiang	2016	+	+	+	+	+	+	+
	PP488481	PP706997	KY362321	A	Zhejiang	2023	+	+	+	+	+	–	+
	PV774368	PV774394	PV774377	B	Zhejiang	2016	–	+	+	+	+	+	+
	OM451720	KY965075	OM451726	A	Henan	2014	+	+	+	–	–	+	+

The “Recombinant Sequence” column lists the isolate inferred to be recombinant, while “Minor Parental Sequence” and “Major Parental Sequence” indicate the likely parental isolates contributing to the recombinant. Recombination detection methods are indicated across the columns: RDP, GENECONV, Bootscan, MaxChi, Chimaera, SiScan, and 3Seq. A “+” denotes that the event was supported by the respective method, whereas a “–” indicates no support.

Table S7. S-segment sequences used in the phylogeographic analysis (*n* = 207).

Isolation	Year	Location	Accession	Genotype
SPL010A	2005	Japan	AB817999	B
SPL032A	2005	Japan	AB818001	B
SPL030A	2010	Japan	AB818000	B
ZJZHSH-LU/China/10/2011	2011	Zhoushan	KR017812	B
YG1	2012	Japan	AB817995	B
SPL003A	2012	Japan	AB817996	B
SPL120A	2012	Japan	AB985554	B
Gangwon/Korea/2012	2012	Republic of Korea	KF358693	C
ZJZHSH-HYC/China/05/2012	2012	Zhoushan	KR017809	B
ZJZHSH-ZLD/China/06/2012	2012	Zhoushan	KR017813	B
ZJZHSH-WSH/China/08/2012	2012	Zhoushan	KR017816	A
ZJZHSH-LHZH/China/06/2012	2012	Zhoushan	KR017819	B
ZJZHSH-CXD/China/05/2012	2012	Zhoushan	KR017820	A
ZJzs028_2012	2012	Daishan	PV774380	B
SPL054A	2013	Japan	AB985524	B
SPL070A	2013	Japan	AB985532	B
SPL107A	2013	Japan	AB985549	B
ZJ2013-06	2013	Zhoushan	KP280205	B
ZJZHSH-FZHR/China/07/2013	2013	Zhoushan	KR017824	A
DS03/CHN/2013	2013	Ningbo	KR698320	B
NB08/CHN/2013	2013	Ningbo	KR698323	A
NB24/CHN/2013	2013	Ningbo	KR698324	B
NB34/CHN/2013	2013	Ningbo	KR698327	A
TNB1580	2013	Japan	LC579715	A
TNB1590	2013	Japan	LC579718	A
KAGNH2	2013	Republic of Korea	MG737195	B
KAJNH	2013	Republic of Korea	MG737197	A
KAICH	2013	Republic of Korea	MG737198	A
KAJJH2	2013	Republic of Korea	MG737199	B
KAGWH2	2013	Republic of Korea	MG737203	A
KADGH5	2013	Republic of Korea	MG737210	B
KACNH	2013	Republic of Korea	MG920818	D
HZ2013-1	2013	Hangzhou	PP706953	D
HZ2013-2	2013	Hangzhou	PP706954	B
ZJnb009_2013	2013	Ningbo	PV774338	B
ZJnb011_2013	2013	Ningbo	PV774340	A
ZJzs027_2013	2013	Daishan	PV774379	B
ZJzs042_2013	2013	Daishan	PV774392	B
ZJzs043_2013	2013	Zhoushan	PV774393	B
ZJzs044_2013	2013	Zhoushan	PV774394	A
ZJzs046_2013	2013	Daishan	PV774396	B
SPL128A	2014	Japan	AB985558	B
SPL129A	2014	Japan	AB985559	B
KASJH	2014	Republic of Korea	KP663733	D
KAGBH5	2014	Republic of Korea	KP663739	B
KACNH3	2014	Republic of Korea	KP663745	B
ZJZHSH-LWL/China/08/2014	2014	Zhoushan	KR017825	B
ZJZHSH-WRF/China/08/2014	2014	Zhoushan	KR017826	B
Rodent01/NB/CHN/2014	2014	Ningbo	KR698330	B
Rodent02/NB/CHN/2014	2014	Ningbo	KR698331	B
ZJ2014-01	2014	Huzhou	KY273274	B

Isolation	Year	Location	Accession	Genotype
ZJ2014-02	2014	Taizhou	KY273275	A
SRH187	2014	Japan	LC579709	B
14KS35	2014	Republic of Korea	MG737216	C
14KS39	2014	Republic of Korea	MG737218	C
14KS47	2014	Republic of Korea	MG737222	C
14KS52	2014	Republic of Korea	MG737224	A
14KS58	2014	Republic of Korea	MG737226	D
14KS64	2014	Republic of Korea	MG737228	D
14KS73	2014	Republic of Korea	MG737230	B
ZJZHSH-LWLTick/China/07/2014	2014	Zhoushan	MT236317	B
HZ2014-1	2014	Hangzhou	PP706956	A
ZJnb015_2014	2014	Ningbo	PV774344	A
ZJnb016_2014	2014	Ningbo	PV774345	A
ZJnb018_2014	2014	Ningbo	PV774347	A
ZJnb022_2014	2014	Ningbo	PV774351	B
ZJzs030_2014	2014	Daishan	PV774382	B
ZJzs033_2014	2014	Daishan	PV774383	A
ZJzs034_2014	2014	Zhoushan	PV774384	A
CB2	2015	Republic of Korea	KY789440	A
15KS8	2015	Republic of Korea	MG737232	C
15MS31	2015	Republic of Korea	MG737235	C
15KS25	2015	Republic of Korea	MG737241	B
15MS104	2015	Republic of Korea	MG737248	B
15KS57	2015	Republic of Korea	MG737250	A
15KS64	2015	Republic of Korea	MG737254	A
15KS73	2015	Republic of Korea	MG737258	C
15MS65	2015	Republic of Korea	MG920824	B
HZ2015-1	2015	Hangzhou	PP706957	A
ZJnb023_2015	2015	Ningbo	PV774352	A
ZJnb024_2015	2015	Ningbo	PV774353	A
ZJzs019_2015	2015	Daishan	PV774371	B
ZJzs022_2015	2015	Hangzhou	PV774374	D
ZJzs024_2015	2015	Daishan	PV774376	A
ZJzs026_2015	2015	Daishan	PV774378	B
ZJzs048_2015	2015	Ningbo	PV774398	A
ZJzs051_2015	2015	Daishan	PV774401	B
ZJzs059_2015	2015	Shengsi	PV774409	B
ZJDH-SCH/05/2016	2015	Zhoushan	PV815690	A
A17	2016	Japan	LC536536	B
N2	2016	Japan	LC536537	B
N10	2016	Japan	LC536540	B
16KS15	2016	Republic of Korea	MF094782	D
16KS26	2016	Republic of Korea	MF094789	B
16KS31	2016	Republic of Korea	MF094793	B
16KS33	2016	Republic of Korea	MF094794	D
16KS45	2016	Republic of Korea	MF094799	A
16KS52	2016	Republic of Korea	MF094802	D
16KS89	2016	Republic of Korea	MG737275	C
16MS300	2016	Republic of Korea	MG737278	C
16MS315	2016	Republic of Korea	MG737281	B
16KS96	2016	Republic of Korea	MG737283	A
16KS115	2016	Republic of Korea	MG737294	A

Isolation	Year	Location	Accession	Genotype
ZJnb001_2016	2016	Taizhou	PV774073	B
ZJzs001_2016	2016	Daishan	PV774355	B
ZJzs002_2016	2016	Zhoushan	PV774356	A
ZJzs003_2016	2016	Daishan	PV774357	A
ZJzs007_2016	2016	Daishan	PV774361	B
ZJzs018_2016	2016	Daishan	PV774370	A
ZJDH-YJH/06/2016	2016	Zhoushan	PV815688	A
ZJDH-YSQ/06/2016	2016	Zhoushan	PV815689	A
SkrP/2017	2017	Japan	LC325238	B
ArtSp/2017	2017	Japan	LC325239	B
JaOF2017-7	2017	Japan	LC590892	A
CUK-JJ01	2017	Republic of Korea	MH937372	B
CB7	2017	Republic of Korea	MK301485	B
HLP23_VE6	2017	Republic of Korea	MN450763	B
HZ2017-1	2017	Hangzhou	PP706958	E
ZJnb002_2017	2017	Ningbo	PV774074	A
ZJnb003_2017	2017	Ningbo	PV774075	B
ZJnb004_2017	2017	Ningbo	PV774333	B
ZJzs009_2017	2017	Daishan	PV774363	B
ZJzs055_2017	2017	Zhoushan	PV774405	A
ZJzs056_2017	2017	Daishan	PV774406	B
ZJzs057_2017	2017	Daishan	PV774407	D
ZJDH-YWX/05/2017	2017	Zhoushan	PV815691	A
Cat/Miyazaki/2018	2018	Japan	LC462229	B
H9/Miyazaki/2018	2018	Japan	LC462230	B
Ydog34	2018	Japan	LC570784	A
JaOT2018-6	2018	Japan	LC590895	B
CBCB01	2018	Republic of Korea	MZ501597	B
HZ2018-2	2018	Hangzhou	PP706959	E
HZ2018-13	2018	Hangzhou	PP706960	A
HZ2018-15	2018	Hangzhou	PP706961	A
HZ2018-23	2018	Hangzhou	PP706962	C
HZ2018-44	2018	Hangzhou	PP706963	C
HZ2018-46	2018	Hangzhou	PP706964	C
ZJnb005_2018	2018	Ningbo	PV774334	B
ZJnb006_2018	2018	Ningbo	PV774335	A
ZJnb025_2018	2018	Taizhou	PV774354	E
ZJzs053_2018	2018	Daishan	PV774403	B
1981_TKY	2019	Japan	LC549337	B
Ydog63	2019	Japan	LC570793	B
Ydog70	2019	Japan	LC570796	A
N100	2019	Japan	LC570799	B
AS7	2019	Republic of Korea	OQ644302	B
AS8	2019	Republic of Korea	OQ644303	B
JN13	2019	Republic of Korea	OQ644308	B
BNY19003	2019	Huzhou	PP488475	B
HZ2019-10	2019	Hangzhou	PP706965	A
HZ2019-32	2019	Hangzhou	PP706966	A
HZ2019-38	2019	Hangzhou	PP706967	D
JJCB01	2020	Republic of Korea	MZ501600	B
20ZJTZ01	2020	Taizhou	MZ773049	B
20ZJTZ12	2020	Taizhou	MZ773050	B

Isolation	Year	Location	Accession	Genotype
20ZJTZ11	2020	Taizhou	MZ773051	A
20ZJTZ08	2020	Taizhou	MZ773054	A
20ZJTZ06	2020	Taizhou	MZ773056	A
20ZJTZ04	2020	Taizhou	MZ773058	B
IP417	2020	Republic of Korea	OK423755	B
CB16	2020	Republic of Korea	PP656918	B
HZ2020-2	2020	Hangzhou	PP706969	B
SFTSV/human/1/Kochi/2021	2021	Japan	LC663817	B
SFTSV/cat/1/Kochi/2021	2021	Japan	LC663820	B
JY1	2021	Japan	LC730375	B
JJCB01/2021	2021	Republic of Korea	OR797608	B
BNY21002	2021	Huzhou	PP488478	B
HZ2021-1	2021	Hangzhou	PP706970	B
HZ2021-4	2021	Hangzhou	PP706971	B
HZ2021-5	2021	Hangzhou	PP706972	E
HZ2021-6	2021	Hangzhou	PP706973	A
N39	2022	Republic of Korea	OR113705	A
N50	2022	Republic of Korea	OR113706	B
JJCB01/2022	2022	Republic of Korea	OR797607	B
GGCB02/2022	2022	Republic of Korea	OR805358	B
BNY22005	2022	Huzhou	PP488479	D
HZ2022-2	2022	Hangzhou	PP706974	B
HZ2022-5	2022	Hangzhou	PP706975	B
HZ2022-7	2022	Hangzhou	PP706977	A
HZ2022-10	2022	Hangzhou	PP706979	A
HZ2022-11	2022	Hangzhou	PP706980	A
HZ2022-13	2022	Hangzhou	PP706981	E
BNY23017	2023	Huzhou	PP488480	D
CB14	2023	Republic of Korea	PP656912	B
CB15	2023	Republic of Korea	PP656915	B
CB17	2023	Republic of Korea	PP656921	B
HZ2023-9	2023	Hangzhou	PP706987	C
HZ2023-10	2023	Hangzhou	PP706988	D
HZ2023-18	2023	Hangzhou	PP706996	B
HZ2023-19	2023	Hangzhou	PP706997	D
HZ2023-20	2023	Hangzhou	PP706998	E
HZ2023-23	2023	Hangzhou	PP707000	D
HZ2023-24	2023	Hangzhou	PP707001	A
HZ2023-26	2023	Hangzhou	PP707002	B
HZ2023-28	2023	Hangzhou	PP707004	A
HZ2023-30	2023	Hangzhou	PP707006	A
HZ2023-31	2023	Hangzhou	PP707007	B
HZ2023-33	2023	Hangzhou	PP707009	E
HZ2023-34	2023	Hangzhou	PP707010	C
HZ2023-37	2023	Hangzhou	PP707013	E
HZ2023-38	2023	Hangzhou	PP707014	C
AJH24006	2024	Huzhou	PX306821	B
AJH24036	2024	Huzhou	PX306822	A
AJH24047	2024	Huzhou	PX306823	B
AJT24074	2024	Huzhou	PX306827	B
AJH25009	2025	Huzhou	PX306824	B
AJH25015	2025	Huzhou	PX306825	B

Table S8. Host range and geographic distribution of SFTSV.

Class	Order	Family	Host	Location	Accession counts
Arachnida	Ixodida	Ixodidae	Tick	Anhui	1
Arachnida	Ixodida	Ixodidae	Tick	Beijing	2
Arachnida	Ixodida	Ixodidae	Tick	China	20
Arachnida	Ixodida	Ixodidae	Tick	Guizhou	1
Arachnida	Ixodida	Ixodidae	Tick	Henan	2
Arachnida	Ixodida	Ixodidae	Tick	Hubei	15
Arachnida	Ixodida	Ixodidae	Tick	Japan	30
Arachnida	Ixodida	Ixodidae	Tick	Jiangsu	9
Arachnida	Ixodida	Ixodidae	Tick	Jilin	6
Arachnida	Ixodida	Ixodidae	Tick	Jinagsu	3
Arachnida	Ixodida	Ixodidae	Tick	Shandong	6
Arachnida	Ixodida	Ixodidae	Tick	Republic of Korea	30
Arachnida	Ixodida	Ixodidae	Tick	Zhejiang	11
Insecta	Diptera	Tabanidae	Tabanus	Henan	2
Mammalia	Artiodactyla	Bovidae	Bovine	Taiwan	1
Mammalia	Artiodactyla	Bovidae	Cattle	Shandong	1
Mammalia	Artiodactyla	Bovidae	Goat	Jinagsu	3
Mammalia	Artiodactyla	Bovidae	Goat	Republic of Korea	3
Mammalia	Artiodactyla	Bovidae	Sheep	Hubei	2
Mammalia	Artiodactyla	Bovidae	Sheep	Shandong	1
Mammalia	Artiodactyla	Camelidae	Camel	Beijing	3
Mammalia	Artiodactyla	Cervidae	Water deer	Republic of Korea	3
Mammalia	Carnivora	Canidae	Dog	Japan	19
Mammalia	Carnivora	Canidae	Dog	Jiangsu	3
Mammalia	Carnivora	Canidae	Dog	Shandong	1
Mammalia	Carnivora	Canidae	Dog	Republic of Korea	27
Mammalia	Carnivora	Canidae	Dog	Thailand	3
Mammalia	Carnivora	Canidae	Fox	Shandong	6
Mammalia	Carnivora	Felidae	Cat	Beijing	1
Mammalia	Carnivora	Felidae	Cat	Japan	92
Mammalia	Carnivora	Felidae	Cat	Republic of Korea	5
Mammalia	Carnivora	Felidae	Cheetah	Japan	6
Mammalia	Carnivora	Mustelidae	Mink	Shandong	6
Mammalia	Carnivora	Mustelidae	Yellow weasel	Henan	3
Mammalia	Carnivora	Procyonidae	Raccoon	Japan	12

Class	Order	Family	Host	Location	Accession counts
Mammalia	Chiroptera	Vespertilionidae	Bat	Hubei	3
Mammalia	Eulipotyphla	Erinaceidae	Hedgehog	China	3
Mammalia	Eulipotyphla	Erinaceidae	Hedgehog	Jiangsu	6
Mammalia	Primates	Hominidae	Homo sapiens	Anhui	155
Mammalia	Primates	Hominidae	Homo sapiens	Beijing	10
Mammalia	Primates	Hominidae	Homo sapiens	China	799
Mammalia	Primates	Hominidae	Homo sapiens	Guangdong	4
Mammalia	Primates	Hominidae	Homo sapiens	Hebei	9
Mammalia	Primates	Hominidae	Homo sapiens	Henan	2934
Mammalia	Primates	Hominidae	Homo sapiens	Hubei	557
Mammalia	Primates	Hominidae	Homo sapiens	Japan	411
Mammalia	Primates	Hominidae	Homo sapiens	Jiangsu	170
Mammalia	Primates	Hominidae	Homo sapiens	Jiangxi	15
Mammalia	Primates	Hominidae	Homo sapiens	Jilin	3
Mammalia	Primates	Hominidae	Homo sapiens	Liaoning	96
Mammalia	Primates	Hominidae	Homo sapiens	Shandong	265
Mammalia	Primates	Hominidae	Homo sapiens	Republic of Korea	574
Mammalia	Primates	Hominidae	Homo sapiens	Taiwan	3
Mammalia	Primates	Hominidae	Homo sapiens	Thailand	7
Mammalia	Primates	Hominidae	Homo sapiens	Zhejiang	664
Mammalia	Rodentia	Muridae	Mouse	Zhejiang	6
Mammalia	Rodentia	Muridae	Rat	Henan	3
Mammalia	Rodentia	Sciuridae	Squirrel	Anhui	3
Aves	Anseriformes	Anatidae	Duck	NA	NA
Aves	Anseriformes	Anatidae	Goose	NA	NA
Mammalia	Artiodactyla	Bovidae	Cattle	NA	NA
Mammalia	Artiodactyla	Bovidae	Goat	NA	NA
Mammalia	Artiodactyla	Bovidae	Sheep	NA	NA
Mammalia	Artiodactyla	Cervidae	Sika deer	NA	NA
Mammalia	Artiodactyla	Cervidae	Water deer	NA	NA
Mammalia	Artiodactyla	Suidae	Wild boar	NA	NA
Mammalia	Carnivora	Canidae	Wolf	NA	NA
Mammalia	Carnivora	Canidae	Raccoon dog	NA	NA
Mammalia	Carnivora	Felidae	Cat	NA	NA
Mammalia	Carnivora	Felidae	Leopard	NA	NA
Mammalia	Carnivora	Herpestidae	Meerkat	NA	NA
Mammalia	Carnivora	Mustelidae	Japanese Badger	NA	NA

Class	Order	Family	Host	Location	Accession counts
Mammalia	Carnivora	Mustelidae	Yellow weasel	NA	NA
Mammalia	Carnivora	Mustelidae	Mink	NA	NA
Mammalia	Carnivora	Procyonidae	Raccoon	NA	NA
Aves	Columbiformes	Columbidae	Spotted dove	NA	NA
Aves	Columbiformes	Columbidae	Pigeon	NA	NA
Mammalia	Eulipotyphla	Erinaceidae	Hedgehog	NA	NA
Mammalia	Eulipotyphla	Soricidae	Shrew	NA	NA
Aves	Galliformes	Phasianidae	Chicken	NA	NA
Arachnida	Ixodida	Ixodidae	Tick	NA	NA
Mammalia	Rodentia	Cricetidae	Hamster	NA	NA
Mammalia	Rodentia	Muridae	Mouse	NA	NA

Hosts are organized by taxonomic class, order, and family. The “Accession counts” column indicates the number of available GenBank sequences for each specific host-location combination. The dataset encompasses both invertebrate vectors and vertebrate hosts, reflecting the broad host tropism of SFTSV. NA indicates not available. Data were compiled from Cui et al. [1] and GenBank annotations.

References

1. Cui H, Shen S, Chen L, Fan Z, Wen Q, Xing Y, et al. Global epidemiology of severe fever with thrombocytopenia syndrome virus in human and animals: A systematic review and meta-analysis. Lancet Reg Health West Pac. 2024;48:101133.
